# Supplementary material for: Prevalence of hepatitis B virus infection among general population of Armenia in 2021 and factors associated with it: a cross-sectional study
Source: BMJ Open. 2024 Feb 7;14(2):e080281. doi: 10.1136/bmjopen-2023-080281 (PMC10860008; doi:10.1136/bmjopen-2023-080281)
Supplement: Supplementary data [file bmjopen-2023-080281supp001.pdf]

Knowledge, Attitude, and Practices Questionnaire on Infectious Diseases, Chronic Hepatitis B and C Virus Infections and Associated Risk Factors

Interviewer

_ _ _ _ _ _ _

Participant ID

_ _/_ _/_ _ _/

Blood sampling for Hepatitis

Do not read out loud. Mark as it is mentioned in Excel file.

() Yes → *Show Section 17*

() No → *Skip Section 17*

Date

dd/mm/yy

_____ / _____ / __________

Start of the interview

___:___ (hour:min)

1. Socio-demographic characteristics

The following questions are about your demographic characteristics

1.1 Your nationality

( ) Armenian

( ) Russian

( ) Yazidi

( ) Other, please specify ____________________________

( ) [prefer not to answer]

1.2 Place of residence

( ) City

( ) Village

( ) [prefer not to answer]

Demirchyan A, *et al. BMJ Open* 2024; 14:e080281. doi: 10.1136/bmjopen-2023-080281

1.3 Your gender

Do not read out loud

( ) Male

( ) Female

1.4 How old are you in the number of years completed? (How old were you on your last birthday)?

dd/mm/yy

_____ / _______ / ____

( ) [prefer not to answer]

1.5 Your highest level of education

Read out all of the options

( ) Incomplete secondary (less than 10 years)
( ) Secondary (10-12 years: high school)
( ) Vocational (12-13 years)
( ) University
( ) Post-graduate
( ) [prefer not to answer]

1.6 How many members are in your family, including yourself?

________________ members

( ) [prefer not to answer]

1.7 Are you currently employed?

Read out all of the options

() Yes, please specify what is your occupation?_________________

() No, I am not employed
() No, I am retired
() No, I am a student
() I work from home/ have my own business
() I am a seasonal worker in foreign countries
() I am a farmer
() Other (specify) ______________________
( ) [prefer not to answer]

Demirchyan A, *et al. BMJ Open* 2024; 14:e080281. doi: 10.1136/bmjopen-2023-080281

1.8 On average, how much money does your family spend monthly? (Choose one option)

( ) Less than 100,000 AMD
( ) From 101,000 to 200,000 AMD
( ) From 201,000 to 300,000 AMD
( ) From 301,000 to 400,000 AMD
( ) Above 401,000 AMD
( ) Don't know/ Refusal

2. The following questions refer to health and infectious disease literacy.

2.1 How often do you need to have someone help you when you read instructions, pamphlets, or other written material from your doctor or pharmacy? (R*ead the response options, except [prefer not to answer] option)*

1. Never
2. Rarely
3. Sometimes
4. Often
5. Always
6. [prefer not to answer]

On a scale from 1 to 7, how easy or difficult would you say it is to…:

Note to interviewers: Repeat the full question as needed

2.2…find the information you need related to infectious diseases (for example, hepatitis, measles, influenza, COVID-19, HIV, etc.)?

Very difficult [1] [2] [3] [4] [5] [6] [7] Very easy

( ) [prefer not to answer]

2.3…understand information about what to do if you think you have an infectious disease (for example, measles, influenza, COVID-19, HIV, etc.)?

Very difficult [1] [2] [3] [4] [5] [6] [7] Very easy

( ) [prefer not to answer]

2.4…judge if the information about infectious diseases in the media is reliable?

Very difficult [1] [2] [3] [4] [5] [6] [7] Very easy

( ) [prefer not to answer]

Demirchyan A, *et al. BMJ Open* 2024; 14:e080281. doi: 10.1136/bmjopen-2023-080281

2.5 …understand restrictions and recommendations of authorities regarding infectious diseases?

Very difficult [1] [2] [3] [4] [5] [6] [7] Very easy

( ) [prefer not to answer]

2.6 …follow the recommendations on how to protect yourself from infectious diseases?

Very difficult [1] [2] [3] [4] [5] [6] [7] Very easy

( ) [prefer not to answer]

2.7 …understand recommendations about when to stay at home from work/school, and when not to?

Very difficult [1] [2] [3] [4] [5] [6] [7] Very easy

( ) [prefer not to answer]

2.8 …follow recommendations about when to stay at home from work/school, and when not to?

Very difficult [1] [2] [3] [4] [5] [6] [7] Very easy

( ) [prefer not to answer]

2.9 …understand recommendations about when to engage in social activities, and when not to?

Very difficult [1] [2] [3] [4] [5] [6] [7] Very easy

( ) [prefer not to answer]

2.10…follow recommendations about when to engage in social activities, and when not to?

Very difficult [1] [2] [3] [4] [5] [6] [7] Very easy

( ) [prefer not to answer]

3. The following questions are about infectious disease-related knowledge and behaviors

3.1 What is the most effective measure for preventing measles? *(Do not read, check all that apply)*

1. Vaccination
2. Using medical masks
3. Avoiding contact with those who have measles
4. Other (specify)__________________________
5. Don't know
6. [prefer not to answer]

3.2 Please indicate whether you agree or disagree with the following statement:

- Antibiotics are effective in treating influenza.
  1. Agree
  2. Disagree
  3. Don't know
  4. [prefer not to answer]

3.3 Please indicate whether you agree or disagree with the following statement:

- Influenza is the same as common cold.
  1. Agree
  2. Disagree
  3. Don't know
  4. [prefer not to answer]

3.4 Please indicate whether you agree or disagree with the following statement:

- Influenza is caused by a virus.
  1. Agree
  2. Disagree
  3. Don't know
  4. [prefer not to answer]

3.5 Do you usually cover your mouth when coughing or sneezing in public places?

1. Yes
2. No
3. [prefer not to answer]

3.6 Do you usually wash your hands before eating?

1. Yes
2. No
3. [prefer not to answer]

4. The following questions are about the symptoms you might have experienced in the past.

4.1 Have you had fever (≥ 37) within the **last six months**?

( ) Yes→ Date (mm/yy) and the highest temperature, (C°):

____

( ) No

( ) Do not remember

( ) [prefer not to answer]

Demirchyan A, *et al. BMJ Open* 2024; 14:e080281. doi: 10.1136/bmjopen-2023-080281

4.2 Have you had any respiratory symptoms (sore throat, cough, runny nose, shortness of breath, etc.) in the last 3 months?

( ) If yes, specify the date of the last episode → mm/yy: ____

( ) No → Move to section 12.4

( ) [prefer not to answer] → Move to section 12.4

4.3 Please indicate, if you have had any of the below-mentioned symptoms in the last 6 months?

Respiratory symptoms

	**Yes**	**No**	**[prefer not to answer]**
4.3.a. Sore throat	( )	( )	( )
4.3.b.Cough	( )	( )	( )
4.3.c. Runny nose	( )	( )	( )
4.3.d. Shortness of breath	( )	( )	( )

4.4. Other symptoms

	**Yes**	**No**	**[prefer not to answer]**
4.4.a. Muscle ache	( )	( )	( )
4.4.b. Joint ache	( )	( )	( )
4.4.c. Chills	( )	( )	( )
4.4.d. Rash	( )	( )	( )
4.4.e. Chest pain	( )	( )	( )
4.4.f Vomiting	( )	( )	( )
4.4.g Headache	( )	( )	( )
4.4.h Nausea	( )	( )	( )

Demirchyan A, *et al. BMJ Open* 2024; 14:e080281. doi: 10.1136/bmjopen-2023-080281

	**Yes**	**No**	**[prefer not to answer]**
4.4.i Loss of taste	( )	( )	( )
4.4.g Loss of smell	( )	( )	( )
4.4.k. Loss of appetite	( )	( )	( )
4.4.l Abdominal pain	( )	( )	( )
4.4.m Diarrhea	( )	( )	( )
4.4.n Conjunctivitis	( )	( )	( )
4.4.o Nose bleed	( )	( )	( )
4.4.p Fatigue	( )	( )	( )
4.4.q Seizures	( )	( )	( )
4.4.r Altered consciousness	( )	( )	( )

4.5.a Other neurological signs

( ) Yes (specify): ____

( ) no

( ) [prefer not to answer]

4.5.b Other symptoms

( ) Yes (specify): ____

( ) No

( ) [prefer not to answer]

5. The following questions are about accompanying diseases and conditions.

Please indicate if you have had any of the below mentioned diseases/conditions.

5.1. Pregnancy
(Do not ask this question to males)

( ) Yes (specify the trimester: first, second, third, unknown): ______________________________

( ) No

( ) [prefer not to answer]

*Read all of the options, marking **yes** or **no** in front of each. Do not read [prefer not to answer] option.*

	**Yes**	**No**	**Don't know/Refuse to answer**
5.2. Obesity	( )	( )	
5.3. Cancer	( )	( )	
5.4. Diabetes	( )	( )	
5.5. HIV/ other immune deficiency	( )	( )	
5.6. Heart disease	( )	( )	
5.7. Asthma (requiring medication)	( )	( )	
5.8. Chronic lung disease (non-asthma)	( )	( )	
5.9. Chronic liver disease	( )	( )	
5.10. Chronic hematological disorder	( )	( )	
5.11. Chronic kidney disease	( )	( )	
5.12. Chronic neurological impairment/disease	( )	( )	
5.13. Organ or bone marrow recipient	( )	( )	

Demirchyan A, *et al. BMJ Open* 2024; 14:e080281. doi: 10.1136/bmjopen-2023-080281

5.14. Other accompanying disease(s)

( ) Yes (specify) ____

( ) No

( ) [prefer not to answer]

6. Have you smoked more than 100 cigarettes during your life?

( ) Yes

( ) No

( ) [prefer not to answer]

6.a How would you describe your current smoking status?

Read out all of the options

( ) I smoke everyday – go to question 16.b

( ) I smoke less than everyday

( ) I quit smoking less than a year ago

( ) I quit smoking more than a year ago

( ) [prefer not to answer]

6.b How many cigarettes do you smoke per day? ___________

7. The following questions are about factors that might be associated with Hepatitis C and B infection in the population

7.1 How often do you visit the dentist to have your teeth cleaned, tooth extracted, braces put on, or root canals?

( ) Twice per year

( ) Once a year

( ) Once every 2 years

( ) Once every 3-5 years

( ) It has been more than 5 years

( ) Never

( ) Don’t know/remember/[prefer not to answer]

Demirchyan A, *et al. BMJ Open* 2024; 14:e080281. doi: 10.1136/bmjopen-2023-080281

7.2. Have you received any blood transfusions in your lifetime?

( ) Yes, before 199X
( ) Yes, in or after 199X
( ) No
( ) [prefer not to answer]

7.3 Do you have any tattoos?

( ) Yes
( ) No
( ) [prefer not to answer]

7.4 Have you ever been told by a doctor or other health worker that you had hepatitis B or C? *(multiple answers possible)*

( ) Yes hepatitis B
( ) Yes hepatitis C
( ) Yes, but don't know the type
( ) Neither hepatitis B nor hepatitis C
( ) I am vaccinated against hepatitis B
( ) Don’t know/remember/refuse to answer

7.5 Have you ever been in prison (translation: long-term detainment) or jail (translation: short-term detainment)?

( ) Yes
( ) No
( ) [prefer not to answer]

End of interview

___:___ (hour:min)

Notes

____

Thank you!

Demirchyan A, *et al. BMJ Open* 2024; 14:e080281. doi: 10.1136/bmjopen-2023-080281
