# Supplementary material for: Prevalence of hepatitis B virus infection among general population of Armenia in 2021 and factors associated with it: a cross-sectional study
Source: BMJ Open. 2024 Feb 7;14(2):e080281. doi: 10.1136/bmjopen-2023-080281 (PMC10860008; doi:10.1136/bmjopen-2023-080281)
Supplement: Author's manuscript [file bmjopen-2023-080281.draft_revisions.pdf]

BMJ Open is committed to open peer review. As part of this commitment we make the peer review history of every article we publish publicly available.

When an article is published we post the peer reviewers' comments and the authors' responses online. We also post the versions of the paper that were used during peer review. These are the versions that the peer review comments apply to.

The versions of the paper that follow are the versions that were submitted during the peer review process. They are not the versions of record or the final published versions. They should not be cited or distributed as the published version of this manuscript.

BMJ Open is an open access journal and the full, final, typeset and author-corrected version of record of the manuscript is available on our site with no access controls, subscription charges or pay-per-view fees (http://bmjopen.bmj.com).

If you have any questions on BMJ Open's open peer review process please email info.bmjopen@bmj.com

BMJ Open

Prevalence of hepatitis B virus infection among general population of Armenia in 2021 and factors associated with it: a cross-sectional study

Journal:	*BMJ Open*
Manuscript ID	bmjopen-2023-080281
Article Type:	Original research
Date Submitted by the Author:	26-Sep-2023
Complete List of Authors:	Demirchyan , A; American University of Armenia, College of Health Sciences<br>Dudareva, S.; Robert Koch Institut, Department for Infectious Disease Epidemiology<br>Sahakyan, Serine; American University of Armenia, College of Health Sciences<br>Aslanyan, Lusine; American University of Armenia, College of Health Sciences<br>Muradyan, Diana; American University of Armenia, College of Health Sciences<br>Musheghyan, Lusine; American University of Armenia, College of Health Sciences<br>Mozalevskis, Antons; World Health Organization, Global HIV, Hepatitis and Sexually Transmitted Infections Programmes<br>Sargsyants, Narina; National Institute of Health named after academician Suren Avdalbekyan<br>Ghukasyan, Gayane; World Health Organization Country Office in Armenia<br>Petrosyan, V; American University of Armenia, College of Health Sciences
Keywords:	Epidemiology < INFECTIOUS DISEASES, Infection control < INFECTIOUS DISEASES, Public health < INFECTIOUS DISEASES

SCHOLARONE™
Manuscripts

I, the Submitting Author has the right to grant and does grant on behalf of all authors of the Work (as defined in the below author licence), an exclusive licence and/or a non-exclusive licence for contributions from authors who are: i) UK Crown employees; ii) where BMJ has agreed a CC-BY licence shall apply, and/or iii) in accordance with the terms applicable for US Federal Government officers or employees acting as part of their official duties; on a worldwide, perpetual, irrevocable, royalty-free basis to BMJ Publishing Group Ltd ("BMJ") its licensees and where the relevant Journal is co-owned by BMJ to the co-owners of the Journal, to publish the Work in this journal and any other BMJ products and to exploit all rights, as set out in our licence.

The Submitting Author accepts and understands that any supply made under these terms is made by BMJ to the Submitting Author unless you are acting as an employee on behalf of your employer or a postgraduate student of an affiliated institution which is paying any applicable article publishing charge ("APC") for Open Access articles. Where the Submitting Author wishes to make the Work available on an Open Access basis (and intends to pay the relevant APC), the terms of reuse of such Open Access shall be governed by a Creative Commons licence – details of these licences and which Creative Commons licence will apply to this Work are set out in our licence referred to above.

Other than as permitted in any relevant BMJ Author's Self Archiving Policies, I confirm this Work has not been accepted for publication elsewhere, is not being considered for publication elsewhere and does not duplicate material already published. I confirm all authors consent to publication of this Work and authorise the granting of this licence.

For peer review only

Prevalence of hepatitis B virus infection among general population of Armenia in 2021 and factors associated with it: a cross-sectional study

ABSTRACT

Background Hepatitis B virus (HBV) continues to be a threat to public health, despite the availability of effective anti-HBV vaccination. Knowledge of HBV prevalence is essential for planning disease control initiatives. Yet, no nationally representative studies of HBV markers were conducted in Armenia so far. This study sought to determine the prevalence and associated factors of HBV infection ever in life and chronic HBV infection in Armenia.

Methods A representative cross-sectional seroprevalence study of anti-HBV core antibodies (anti-HBc) and HBV surface antigen (HBsAg) was conducted throughout Armenia using third-generation enzyme immunoassays. In case of HBsAg positivity, HBV DNA and hepatitis D virus (HDV) RNA PCR tests were performed. All tested individuals participated in a phone survey. Risk factors of HBV infection ever in life (anti-HBc positivity) and chronic HBV infection (HBsAg positivity) were identified through fitting logistic regression models.

Results The seroprevalence study included 3,838 individuals 18 years and older. Of them, 90.7% responded to the phone survey. The prevalence of anti-HBc positivity was 14.1% (95% CI 13.1%-15.2%) and HBsAg positivity 0.8% (95% CI 0.5%-1.1%). The viral load was over 10,000 IU/ml for 7.9% of HBsAg-positive individuals. None of the participants was positive for HDV. Risk factors for HBsAg positivity included less than secondary education (95% CI 2.2-19.1), current smoking (95% CI 1.2-5.6), and chronic liver disease (95% CI 3.0-23.7). In addition to these, risk factors for anti-HBc positivity included age (95% CI 1.04-1.05), imprisonment ever in life (95% CI 1.41-4.56), and poor knowledge on infectious diseases (95% CI 1.05-1.67), while living in Yerevan (vs. *marzes*) was protective (95% CI 0.59-0.93).

Conclusion This study provided robust estimates of HBV markers among general population of Armenia. Its findings delineated the need to revise HBV testing and treatment strategies considering higher risk population groups, and improve population knowledge on HBV prevention.

For peer review only - http://bmjopen.bmj.com/site/about/guidelines.xhtml

STRENGTHS AND LIMITATIONS OF THIS STUDY

- ⇒ This is the first nation-wide representative study in Armenia providing robust estimates of prevalence of Hepatitis B infection ever in life and chronic infection among general adult population.
- ⇒ The limited length of the questionnaire did not allow to include all relevant questions to determine all the factors associated with higher odds of being infected with HBV.
- ⇒ As the sample size was calculated for the whole population, hence, we were limited in estimating the prevalence of HBV infection among different groups of population.
- ⇒ Similarly, we might lack power to detect differences among population groups and when performing regression analyses.
- ⇒ At the same time, the sample size was appropriate to measure nationwide robust estimates with narrow confidence intervals and revealed important risk factors to guide the policy decisions to fight the virus.

For peer review only

For peer review only - http://bmjopen.bmj.com/site/about/guidelines.xhtml

INTRODUCTION

Despite the availability of effective vaccination against hepatitis B virus (HBV), it continues to be a global threat to public health, particularly in low- and middle-income countries (LMIC).(1) According to World Health Organization (WHO) 2019 data, 296 million people in the globe live with chronic hepatitis B, and 1.5 million new infections occur annually. The Western Pacific region and the African region account for the highest percentage of the cases.(1) More than a million fatalities globally are attributed to HBV, which is the primary cause of morbidity and mortality from chronic hepatitis, cirrhosis, and hepatocellular carcinoma (HCC), in addition to the acute infection itself.(2, 3)

A common transmission route for the virus is from mother to child during birth and delivery.(4) The virus can also be transmitted during sexual intercourse and through contacting the blood or other body fluids of an infected person.(4) The commonly known risk factors for HBV are being an infant born to an infected mother, injecting drugs or sharing needles, having a partner who is infected with the virus, household contact with someone who has the virus, men having sex with men (MSM), female sex workers, living with HIV, and having occupational exposure to blood and blood products.(5-7) Additionally, studies have shown that sociodemographic factors including older age, male sex, low socio-economic status, and living in poor areas could be risk factors for chronic HBV infection.(8, 9) Other social variables that have been shown to impact chronic HBV prevalence were poor housing, low educational level, and lack of knowledge on HBV prevention.(9)

In regions with high endemicity, where at least 8% of people are chronically infected, hepatitis B is primarily acquired around birth time or during early childhood.(10) Age at the time of infection has an adverse relationship with the chance of progression to chronic infection. If not immunized at birth, up to 90% of infants who are infected prenatally develop chronic HBV infection.(11) About 30% of those infected during infancy and early childhood, and 6% of

For peer review only - http://bmjopen.bmj.com/site/about/guidelines.xhtml

children infected after the age five develop chronic infection. The risk of developing chronic HBV infection is less than 5% if being infected during adulthood.(11)

The global epidemiology of HBV infection has undergone notable changes since a reliable vaccine became widely accessible more than 20 years ago.(12) There has been a decline in the prevalence of chronic HBV infection in many parts of the world, including Europe, the Americas, and Australia, primarily as a result of the vaccination against hepatitis B and improved medical practices, such as screening of blood and blood products, applying safe injection techniques, and implementing infection control policies and procedures.(13)

A noticeable decrease in the incidence of acute viral hepatitis infections was observed in Armenia over the last three decades. The incidence of acute HBV infection steadily decreased from 22.30 in 1990 to 0.17 in 2021 per 100 000 population.(14) Although these data could underestimate the real incidence because of incomplete registration of cases by the healthcare system, it is very likely reflecting true decreasing trend. One of the explanations of this decrease is the high (over 96%) coverage of hepatitis B newborn and infant vaccination introduced in Armenia in1999. Before 1999, only healthcare providers working in high-risk settings were vaccinated against hepatitis B.(15)

Concerning the prevalence of chronic HBV infection, no nationally representative seroprevalence studies of HBV markers were conducted among general population of Armenia before 2021. Different estimates were made at different time points. Based on synthesis of available screening data on blood donors, pregnant women, healthcare providers, and patients, the best guess was that chronic HBV infection was around 2% among general population of Armenia in 2000.(15) In 2013, based on retrospective testing of frozen blood samples of over 10,000 healthy individuals aged from 1 to 60 years for the presence of HBV surface antigen, the median prevalence of chronic HBV infection among general population of Armenia was reported

For peer review only

to be 1.9-2.5%.(16, 17) In the meantime, the prevalence was reported as 1.4% among blood donors and 1.5% among pregnant women.(17)

A recent review study that explored the groups of population at higher risk for chronic HBV infection in the countries of Caucasus and Central Asia, stated that chronic HBV infection was higher (3.7%) among prisoners in Armenia.(18) Concerning other vulnerable groups, the estimates varied across different small-scale studies from 0% to 1.6% for MSM and from 0.3% to 0.9% for migrants (mainly seasonal workers returning from Russia).(18) Yet, the study published in 2013 reported considerably higher prevalence of chronic HBV infection among some population groups in Armenia, including prisoners (4.3%), hemodialysis patients (6.6%), medical workers (7.3%), people who inject drugs (8.8%), and patients with sexually transmitted diseases (16.0%).(17)

For the purpose of assessing national disease prevention and control initiatives including immunization programs, knowledge of the region- and age-specific prevalence of hepatitis B infection is essential. The study presented in this paper was the first attempt to identify the seroprevalence of HBV infection among general adult population of Armenia. The study aimed to determine the prevalence of HBV infection ever in life and chronic HBV infection in Armenia and identify the risk factors of each of these outcomes among the general adult population.

METHODS

Study design, sampling and sample size

This study was a cross-sectional population-based sero-epidemiological study conducted throughout Armenia in May-September, 2021. The study included laboratory testing of blood serum samples of the participants for biomarkers of hepatitis B, hepatitis C, and SARS-CoV-2 viruses, which was shortly followed with a telephone survey among them. The study was primarily designed to measure the biomarkers for SARS-CoV-2 and the hepatitis markers were

For peer review only - http://bmjopen.bmj.com/site/about/guidelines.xhtml

For peer review only

added to it. The current paper reflects the analytical approaches and findings on HBV infection. The methods and results for hepatitis C and SARS-CoV-2 virus infections will be reported elsewhere.

The study frame was general adult population of Armenia aged 18 years and over. We used a proportionate-to-the-population-size cluster sampling design, which included all 11 administrative units of Armenia (the ten *marzes* and Yerevan city). The study participants were recruited from primary healthcare (PHC) facilities, as the coverage of population with PHC is quite high in Armenia (about 97%). We selected one-third of the polyclinics in each administrative unit via proportionate to the served population size random sampling. Simple random sampling was then applied within the national e-health operator "ARMED" to select the study participants from the lists of 18 years old and older individuals served by each chosen polyclinic.

The formula for estimating a proportion ($n = N*X / (X + N – 1)$, where $X = Z_{\alpha/2}^2 *p*(1-p) / MOE$) of the population was used to calculate the sample size for this study. When assuming 30% prevalence of anti-SARS-CoV-2 antibodies, 1.45% margin of error (MOE), type 1 error of 0.05, and confidence level of 95%, the calculated sample size was 3,832.

Data collection

The "ARMED" e-health system provided the list of unique ID numbers selected through simple random sampling to the respective polyclinic's personnel trained for contacting the study participants. The personnel contacted each potential participant by phone, following the script for the first contact, introduced the study and invited her/him to the polyclinic to take part in the study. When the participants came to the PHC facility, the research team obtained a written informed consent and collected blood samples. The participant recruitment in each polyclinic continued until the needed sample size was reached. Blood sampling was done by a trained nurse. Telephone interviews were carried out by trained interviewers within two weeks after the

For peer review only - http://bmjopen.bmj.com/site/about/guidelines.xhtml

blood sampling. The structured questionnaire was administered using electronic tablets where Alchemer online tool was installed.

The study protocol was reviewed by the Ethics Review Committee of the World Health Organization and the Institutional Review Board of the American University of Armenia, and was approved by both as conforming to the principles embodied in the Declaration of Helsinki. If the test result was positive, a trained hepatologist communicated the result to the individual and provided counseling. Negative results were sent to the participants via SMS messages.

Serum tests and definitions

Via venipuncture, 5-10 ml venous blood was collected from each participant and centrifuged immediately for the serum extraction. The serums were kept at 2-8°C for seven days at most before being tested at the laboratory of the NCID. This laboratory was chosen because of having the needed technical and human resources to perform third generation serological and molecular testing with the required quality for all three infections included in the study. The testing procedure for HBV included the following steps: (1) all samples were tested for total anti-HBc antibodies using third-generation enzyme immunoassay (Elecsys® Anti-HBc II-Roche Diagnostics with over 99.3% clinical specificity and 100% sensitivity (19)); (2) if anti-HBc antibodies were detected, HBsAg test was performed (using Elecsys HBsAg-Roche Diagnostics with over 99.8% clinical specificity and 99.9% sensitivity (20)), followed by a confirmatory test if the result was questionable (within 0.9-1.0 COI); (3) if HBsAg test was positive, HBV DNA test was performed to test for the presence of HBV DNA and the viral load (using Amplisens HBV Monitor FL); (4) all samples positive for HBsAg were also tested for hepatitis D virus (HDV) RNA (PCR test using Amplisens). All the applied tests were WHO pre-qualified. The presence of antibodies against HBV core antigen (anti-HBc antibodies) was viewed as

For peer review only - http://bmjopen.bmj.com/site/about/guidelines.xhtml

serological evidence of HBV infection ever in life, while the presence of HBV surface antigen (HBsAg[1]) served as serological evidence of chronic HBV infection.

Survey instrument and study variables

The survey tool was a structured questionnaire which included the following domains related to the current study: (1) respondent's sociodemographic characteristics, (2) their self-reported chronic health conditions, (3) symptoms they experienced during the last six months, (4) respondent's knowledge and behavior on prevention of infectious diseases, and (5) some common risk factors of exposure to hepatitis B or C viruses.

The two outcome variables of this study were anti-HBc antibody status and HBsAg status measuring HBV infection ever in life and chronic HBV infection, respectively. The independent variables were grouped into socioeconomic, health status, health literacy, and health behavioral domains. The sociodemographic domain included three continuous (age, family size, and socioeconomic status (SES) score) and four categorical (sex, education, employment, and residence) variables. The health status domain included only categorical (dichotomous) variables: hepatitis C exposure and chronic infection, several other self-reported chronic conditions (diabetes, chronic liver disease, obesity, heart disease, cancer, chronic kidney disease, and asthma requiring medication), as well as symptoms experienced during the six months prior to the survey (vomiting, fatigue, loss of appetite, joint ache, abdominal pain, fever, and nose bleeding). The health literacy domain included a communicable diseases knowledge variable measured by four questions and dichotomized at its lower third, and a score measuring the difficulty of understanding health-related information generated from responses to ten items. The health behavioral domain included the following categorical variables: ever and current regular

[1] The WHO surveillance case definition for chronic HBV infection assumes the presence of biomarkers of the infection combined with the absence of signs and symptoms of acute hepatitis. We applied this definition as coming across a recent infection in a cross-sectional population study is unlikely.

For peer review only - http://bmjopen.bmj.com/site/about/guidelines.xhtml

smoking, having tattoos, ever having been imprisoned, ever receiving blood transfusion, frequency of visiting a dentist, and self-reported anti-HBV vaccination status. A variable on ever having been told by a doctor of being infected with HBV was also included in the analysis.

Patient and Public Involvement Statement

The survey questionnaire was pretested among eligible people and refined based on their feedback. This was the only involvement of public in this research.

Data analysis

We used IBM SPSS Statistics for Windows, version 21 (IBM Corp., Armonk, N.Y., USA) for data management and analysis. All the analysis was weighted by sex and 5-year age groups to closely mimic age and sex distribution of the population of Armenia, as age and sex distribution in the study sample differed from that in the general population due to differences in response rates of different age and sex groups. To estimate the prevalence of HBV infection ever in life and chronic HBV infection, proportions and 95% confidence intervals were calculated for positivity to anti-HBc and HBsAg, respectively. To descriptively analyze independent variables, frequencies and proportions were calculated for categorical variables, and means and standard deviations for continuous variables in the total sample and in each of the outcome groups differentiated based on positivity/negativity to anti-HBc and positivity/negativity to HBsAg. Then univariate logistic regression analysis was done. For the two categorical variables: education and employment, dummies were created to be included in the regression analysis. The linearity of the association of continuous variables with each outcome variable was checked on the logit scale before entering them into logistic regression analysis.(21) The final step was fitting logistic regression models to identify factors independently associated to each of the outcomes. A series of multivariable logistic regression analyses were performed with inclusion of all those variables associated with the outcome at the $p < 0.25$ level in the univariate

For peer review only - http://bmjopen.bmj.com/site/about/guidelines.xhtml

analysis.(21) The variables were entered into logistic regression analysis in different combinations using e*nter* method. The variables not related to the outcome ($p \geq 0.10$) when controlling for the remaining variables in the model were excluded from the model, if their removal did not change the associations between the remaining variables and the outcome. The final models were checked for model fit and collinearity. Cases with any missing values were excluded from the fitted models.

RESULTS

Trained personnel from the selected polyclinics contacted a total of 9 009 eligible individuals throughout the country to invite them for blood testing. Of them, 3 831 (42.5%) individuals agreed and underwent blood testing. Of those tested, 3 476 individuals (90.7%) participated in the phone survey.

Laboratory testing results

The weighted percentage of those positive for anti-HBc antibodies indicating past or present HBV infection was 14.1% (95% CI 13.1%-15.2%). This percentage significantly increased with age (Figure 1) and was slightly higher among men than women (15.0% versus 12.2%, p=0.017). The weighted percentage of those carrying HBsAg – the prevalence of chronic HBV infection – was 0.8% (95% CI 0.5%-1.1%). This estimate was the highest in the age group of 30-39 years old (1.5%, 95% CI 0.8%-2.6%) (Figure 1) and was higher among men compared to women (1.1% versus 0.5%, p=0.048). The measurement of the viral load among those positive for HBsAg showed that for 62% of them, HBV DNA was below 150 IU/ml, for 30% it was between 150 IU/ml and 2,000 IU/ml, and for 8% (n=2) – over 2,000 IU/ml (in the weighted analysis). None of the HBsAg-positive samples were positive for HDV RNA.

For peer review only - http://bmjopen.bmj.com/site/about/guidelines.xhtml

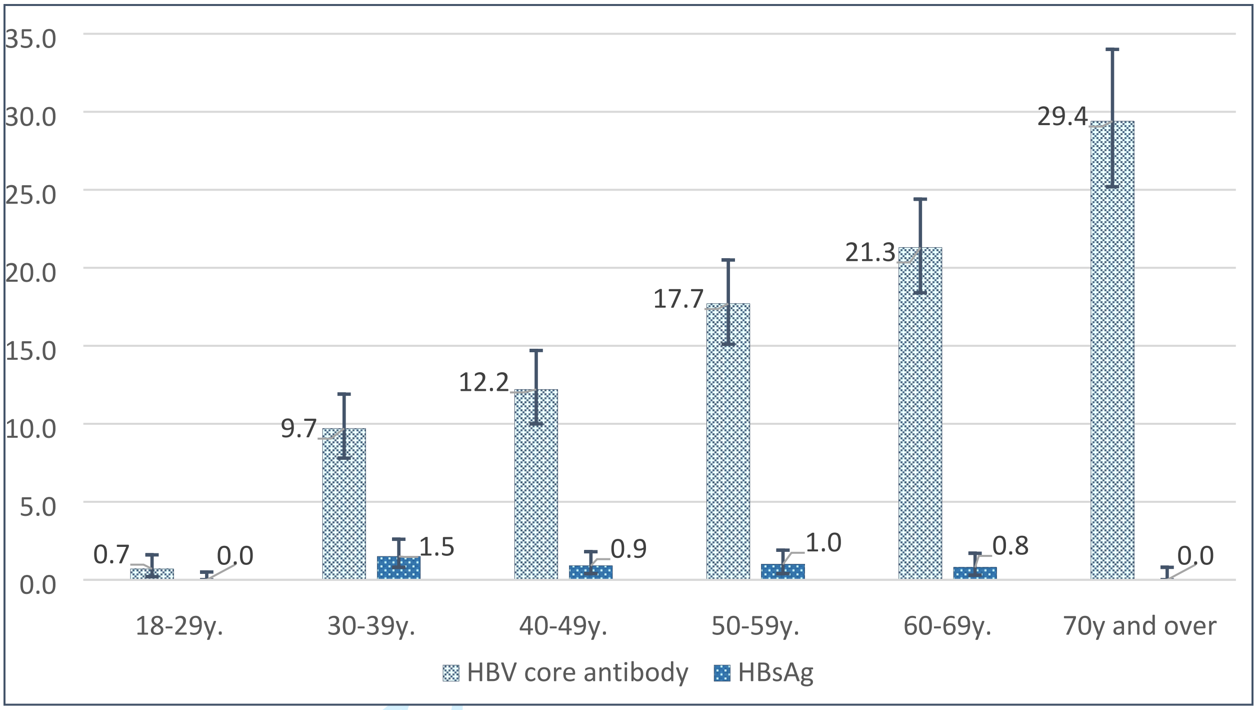

Figure 1 Weighted prevalence estimates of HBV core antibody and HBV surface antigen by 10-year age groups among the study participants, Armenia, 2021.

Results of descriptive comparisons

Table 1 demonstrates the results of weighted crude comparisons of the study variables between the groups infected with HBV ever in life and those never infected with HBV.

Table 1 Distribution of selected variables by participants' anti-HBV core antibody status, Armenia, 2021

			Anti-HBV core antibody			
		N*	Positive (n=469)	Negative (n=3005)	*p-value*	Total (n=3474)
Socioeconomic variables:						
Age, mean (SD)		3473	57.1 (15.7)	44.0 (16.9)	*<0.001*	45.8 (17.3)
Socio-economic status score, mean (SD)		2696	46.8 (34.8)	50.3 (35.3)	*0.075*	49.9 (35.2)
Family size, mean (SD)		3451	4.2 (2.1)	4.4 (1.7)	*0.004*	4.4 (1.8)
Sex:	Female, %	3473	49.4	55.3	*0.017*	54.5
	Male, %		50.6	44.7		45.5
Education:	Below secondary, %	3456	7.7	4.0	*<0.001*	4.5
	Secondary/vocational, %		61.5	52.0		53.3
	University/higher, %		30.8	44.0		42.3
Employment:	Employed/student, %	3438	41.8	58.6	*<0.001*	56.4
	Unemployed/seasonal migrant, %		27.2	28.9		28.7
	Retired/disabled, %		30.9	12.5		15.0

For peer review only - http://bmjopen.bmj.com/site/about/guidelines.xhtml

		N*	Anti-HBV core antibody Positive (n=469)	Negative (n=3005)	*p-value*	Total (n=3474)
Residence:	Yerevan city, %		28.6	37.4	*<0.001*	36.3
	Marzes (provinces), %		71.4	62.6		63.7
Health status variables:						
Anti-Hepatitis C antibodies positive, %		3474	3.6	1.7	*0.011*	2.0
Hepatitis C PCR test positive, %		3474	1.5	0.6	*0.034*	0.7
Diabetes, %		3395	12.4	7.1	*<0.001*	7.8
Chronic liver disease, %		3391	8.4	2.1	*<0.001*	2.9
Obesity, %		3383	13.8	9.5	*0.007*	10.1
Heart disease, %		3390	29.0	12.6	*<0.001*	14.8
Cancer, %		3394	2.0	1.5	*0.408*	1.5
Chronic kidney disease, %		3395	4.6	3.6	*0.286*	3.7
Asthma requiring medication, %		3391	2.0	2.3	*0.864*	2.2
Symptoms experienced during the last six months:						
Vomiting, %		3387	3.3	4.6	*0.268*	4.5
Fatigue, %		3391	41.8	46.0	*0.104*	45.4
Loss of appetite, %		3388	8.9	14.4	*0.001*	13.6
Joint ache, %		3393	37.6	33.4	*0.078*	33.9
Abdominal pain, %		3382	9.4	11.2	*0.257*	11.0
Fever, %		3395	16.5	25.4	*<0.001*	24.2
Nose bleed, %		3387	2.9	4.6	*0.108*	4.4
Health literacy variables:						
Poor knowledge on infectious diseases, %		3474	32.8	24.3	*<0.001*	25.5
Health information understanding score, mean (SD)		3005	52.2 (11.5)	54.4 (9.9)	*<0.001*	54.1 (10.1)
Health behavioral variables:						
Current smoking, %		3389	25.7	20.7	*0.016*	21.4
Ever smoking, %		3395	39.5	34.0	*0.026*	34.7
Having tattoos, %		3380	13.0	10.0	*0.055*	10.4
Undergoing blood transfusion, %		3380	4.0	3.4	*0.487*	3.5
Ever having been imprisoned, %		3387	4.9	1.2	*<0.001*	1.7
Visiting a dentist:	Once in 3-5 years/more, %	3329	52.5	74.3	*<0.001*	71.4
	Less frequently/never, %		47.5	25.7		28.6
Vaccinated against Hepatitis B (self-reported), %		3397	0.2	0.2	*1.000*	0.2
Hepatitis B told by doctor, %		3397	2.7	0.2	*<0.001*	0.5

N, Number of valid responses; HBV, Hepatitis B virus; PCR, Polymerase chain reaction; SD, Standard deviation.
* Weighted estimates; *p-value:* two-sided.

The ever-infected group was older, with lower level of education, from a smaller-size family, and more often – residing in marzes (provinces). The proportion of employed was lower among ever infected group and the proportion of retired/disabled was higher. Anti-hepatitis C antibodies were found among the ever-infected group more frequently. The ever-infected group more

For peer review only

frequently reported several chronic health conditions – diabetes, chronic liver disease, obesity, and heart disease. However, some symptoms experienced during the last six months, including loss of appetite and abdominal pain, were less frequently reported by the ever-infected group. The ever-infected group demonstrated poor knowledge on infectious diseases. The mean score of understanding health related information was also lower among them compared to the never infected group. There were significant differences between the groups in the health behavioral domain as well. Both current and ever smokers were more prevalent among the ever infected. Higher proportion of them were ever imprisoned, and lower proportion used dental services at least once in 3-5 years. A similar negligible proportion of respondents (0.2%) in both groups reported having been vaccinated against hepatitis B.

The characteristics of those participants with and without chronic HBV infection were compared in Table 2.

Table 2 Distribution of selected variables by Hepatitis B surface antigen (HBsAg) status, Armenia, 2021

			HBsAg			
		N*	Positive (n=27)	Negative (n=3447)	*p-value*	Total (n=3474)
Socioeconomic variables:						
Age, mean (SD)		3473	44.7 (11.9)	45.8 (17.4)	*0.734*	45.8 (17.3)
Socio-economic status score, mean (SD)		2696	43.7 (24.3)	49.9 (35.3)	*0.471*	49.9 (35.2)
Family size, mean (SD)		3451	4.2 (1.3)	3.4 (1.8)	*0.595*	4.4 (1.8)
Sex:	Female, %	3473	40.7	54.7	*0.048*	54.5
	Male, %		65.4	45.3		45.5
Education:	Below secondary, %	3455	22.2	4.3	*<0.001*	4.5
	Secondary/vocational, %		44.4	53.3		53.3
	University/higher, %		33.3	42.3		42.3
Employment:	Employed/student, %	3439	48.1	56.4	*0.607*	56.4
	Unemployed/seasonal migrant, %		37.0	28.6		28.7
	Retired/disabled, %		14.8	15.0		15.0
Residence:	Yerevan city, %		29.6	36.3	*0.551*	36.3
	Marzes (provinces), %		70.4	63.7		63.7
Health status variables:						
Anti-Hepatitis C antibodies positive, %		3474	7.4	1.9	*0.097*	2.0
Hepatitis C PCR test positive, %		3474	3.7	0.7	*0.171*	0.7
Diabetes, %		3396	11.1	7.8	*0.464*	7.8
Chronic liver disease, %		3390	19.2	2.8	*0.001*	2.9

For peer review only - http://bmjopen.bmj.com/site/about/guidelines.xhtml

	N*	HBsAg Positive (n=27)	HBsAg Negative (n=3447)	*p-value*	Total (n=3474)
Obesity, %	3384	14.8	10.0	*0.343*	10.1
Heart disease, %	3389	14.8	14.8	*1.000*	14.8
Cancer, %	3395	0.0	1.5	*1.000*	1.5
Chronic kidney disease, %	3395	3.7	3.7	*1.000*	3.7
Asthma requiring medication, %	3391	3.7	2.2	*0.459*	2.2
Symptoms experienced during the last six months:					
Vomiting, %	3388	0.0	4.5	*0.632*	4.5
Fatigue, %	3392	40.7	45.5	*0.700*	45.4
Loss of appetite, %	3388	0.0	13.7	*0.042*	13.6
Joint ache, %	3394	48.1	33.8	*0.151*	33.9
Abdominal pain, %	3382	4.2	11.0	*0.508*	11.0
Fever, %	3395	11.1	24.3	*0.173*	24.2
Nose bleed, %	3387	0.0	4.4	*0.630*	4.4
Health literacy variables:					
Poor knowledge on infectious diseases, %	3474	37.0	25.4	*0.183*	25.5
Health information understanding score, mean (SD)	3005	48.7 (14.4)	54.2 (10.1)	*0.016*	54.1 (10.1)
Health behavioral variables:					
Current smoking, %	3390	44.4	21.2	*0.007*	21.4
Ever smoking, %	3394	53.8	34.6	*0.060*	34.7
Having tattoos, %	3379	19.2	10.3	*0.182*	10.4
Undergoing blood transfusion, %	3380	3.7	3.5	*0.615*	3.5
Ever having been imprisoned, %	3387	11.6	1.6	*0.010*	1.7
Visiting a dentist: Once in 3-5 years/more, %	3330	74.1	71.4	*0.834*	71.4
Less frequently/never, %		25.9	28.6		28.6
Vaccinated against Hepatitis B (self-reported), %	3398	0.0	0.2	*1.000*	0.2
Hepatitis B told by doctor, %	3398	22.2	0.4	*<0.001*	0.5

N, Number of valid responses; PCR, Polymerase chain reaction; SD, Standard deviation.
* Weighted estimates; *p-value:* two-sided.

Those chronically infected had lower education and higher composition of male. They reported chronic liver disease much more frequently. The proportion of those demonstrating poor knowledge on infectious diseases was higher among them and the mean score of understanding health-related information was lower. Smoking was more frequent among them, and they have been imprisoned much more frequently than those free from the virus.

Among those testing positive for anti-HBc, 12 (2.7%) reported that they have ever been told by a doctor that they were infected with hepatitis B. Six of these 12 persons were also positive for HBsAg, thus only 22.2% of those HBsAg positive were aware of their status (Tables 1, 2).

For peer review only

Findings of regression analysis

The fitted logistic regression model of factors independently associated with being ever infected with HBV included the following characteristics – age, education, Yerevan vs. *marz* residence, chronic liver disease, current smoking, imprisonment ever in life, and knowledge on infectious diseases (Table 3).

Table 3 Multivariable logistic regression model of determinants of HBV infection ever in life (positivity for anti-HBV core antibody) among adult population in Armenia, 2021 (valid N=3349)

Characteristics		OR	95% CI	*p- value*
Age, years		1.04	1.04 - 1.05	< 0.001
Education:	Less than secondary	1.61	1.02 - 2.56	0.042
	Secondary/vocational	1.26	1.00 - 1.59	0.052
	University/higher	1.00	ref.	ref.
Living in Yerevan vs. marzes		0.74	0.59 - 0.93	0.011
Chronic liver disease		2.93	1.88 - 4.56	<0.001
Current smoking		1.60	1.25 - 2.06	<0.001
Ever imprisoned		2.53	1.41 - 4.56	0.002
Poor knowledge on infectious diseases		1.32	1.05 - 1.67	0.018

HBV, hepatitis B virus.
N, number of cases.
OR, odds ratio.
CI, confidence interval.

When holding all the other variables constant, each year increase in age increased the likelihood of ever being infected with HBV by 4%. Compared to having university/higher education, having less than secondary education was associated with 1.61 times higher odds, and having secondary/vocational education with 1.26 times higher odds of ever being infected with HBV. Residing in Yerevan was protective – 0.74 times decreasing the odds of HBV infection ever in life. Suffering from chronic liver disease was associated with 2.93 times higher odds of HBV infection ever in life. Ever being imprisoned increased the odds of HBV infection ever in life 2.53 times. Current smoking was also a risk factor for ever having HBV infection, increasing its odds 1.60 times. Poor knowledge of infectious diseases was associated with 1.32 times higher

For peer review only - http://bmjopen.bmj.com/site/about/guidelines.xhtml

odds of having HBV infection ever in life. The model had no collinearity issues with all Variance Inflation Factor (VIF) values being below 1.13.

Several factors associated with having HBV infection ever in life, including education, chronic liver disease, and current smoking, were also significant in the fitted model of determinants of chronic HBV infection (Table 4).

Table 4 Multivariable logistic regression model of determinants of chronic HBV infection (HBsAg positivity) among adult population of Armenia, 2021 (valid N=3349)

Characteristics		OR	95% CI	*p- value*
Education:	Less than secondary	6.44	2.17 - 19.12	<0.001
	Secondary/vocational	1.01	0.42 - 2.45	0.984
	University/higher	1.00	ref.	ref.
Chronic liver disease		8.44	3.00 - 23.72	<0.001
Current smoking		2.56	1.16 - 5.65	0.019
Ever imprisoned		3.33	0.81 - 13.74	0.096

HBV, hepatitis B virus.
HBsAg, Hepatitis B surface antigen.
N, number of cases.
OR, odds ratio.
CI, confidence interval.

Compared to secondary or higher education, less than secondary education increased the odds of having chronic HBV infection 6.4 times. Chronic liver disease was associated with 8.4 times higher odds of having chronic HBV infection. Current smoking increased the likelihood of chronic HBV infection 2.6 times. Ever having been imprisoned was also potentially associated with having chronic HBV infection, as the observed effect range between these variables was suggestive of a weak negative to a strong positive association (OR 3.33; 95% CI 0.81 - 13.74). Again, no collinearity issues were identified in this model with all VIF values being below 1.06.

DISCUSSION

This is the first population-based seroprevalence study to estimate the nationwide prevalence of HBV infection in Armenia. In our study, which included 3,831 participants over 18 years of age

For peer review only - http://bmjopen.bmj.com/site/about/guidelines.xhtml

from Armenia, we found that the weighted prevalence of chronic hepatitis B infection was 0.8% (95% CI 0.5%-1.1%), and 14.1% (95% CI 13.1%-15.2%) had been ever infected with hepatitis B. From those with chronic infection, 7.9% had viral load above 2000 IU/ml. Odds for ever being infected with hepatitis B increased by age. None of the HBsAg-positive participants were positive for HDV RNA.

While the proportion of adult population ever infected with hepatitis B was considerably high in Armenia, the prevalence of chronic infection was low. In comparison to most up-to-date population-based seroprevalence survey results from countries with intermediate hepatitis B endemicity in European Region before introduction of universal hepatitis B vaccination, the prevalence was lower than in a study carried out in Georgia in 2021: the prevalence of chronic hepatitis B among adults in Georgia was 2.7% (95% CI 2.3%-3.4%) and the proportion of persons ever infected with hepatitis B was 21.7% (95% CI 20.4%-23.2%).(22). The results in Armenia are comparable to a recent study done in Ukraine, however, these data are currently not published yet.

Previous data for Armenia based on modeling studies show a higher prevalence of chronic infection. According to a modeling study from 2016, the HBsAg prevalence in Armenia was estimated at 1.9%,(23) and based on the Coalition for Global Hepatitis Elimination estimates, it was gradually decreasing from 3.4% in 1994/95.(24) Surveillance data confirm that there has been a continuous reduction of new cases mainly explained by introduction of hepatitis B vaccination among children in 1999.(15, 17) This will likely contribute to further decrease in prevalence of chronic hepatitis B virus infection in Armenia.(25) The prevalence in 2016 is likely an overestimation as it was relying on proxy data and not a measurement attributable to general population in Armenia. Data from screening of pregnant women in Armenia support the current finding of low prevalence. For the years 2017 – 2021, from over 97,000 pregnant women that underwent screening, 0.66% were HBsAg positive.(26)

For peer review only - http://bmjopen.bmj.com/site/about/guidelines.xhtml

In crude weighted comparisons, the prevalence of both HBsAg and anti-HBc was higher in males than in females, which is similar to other studies.(22, 27, 28) Generally, difference in the prevalence between men and women may be related to either behavioral factors like higher sexual risk behaviors, intravenous drug use and imprisonment in men, or to biological factors.(29) Based on studies, it has been discussed that females usually develop more intense innate, humoral and cellular responses to viral infections, including hepatitis virus, than males.(29, 30) After controlling for other variables, this association, however, disappeared in the model.

While for ever being infected with HBV, it was possible to detect a statistically significant increase in prevalence with increasing age, there were no detectable differences with increasing age in the prevalence of chronic hepatitis B. However, the analysis of HBsAg prevalence by different groups was limited by low number of positive cases and, correspondingly, lack of power with the given sample size. Descriptive results, however, show that there were no chronic hepatitis B cases among those 18-29 years of age. Individuals up to age of 22 have been born after the introduction of birth dose and childhood hepatitis B vaccination in Armenia. This is potentially leading to less cases in this age group. According to the reports submitted to WHO, the impact of hepatitis B vaccination on control of mother to child transmission has been demonstrated in several countries of the WHO European Region with historically intermediate and high hepatitis B endemicity, like Republic of Moldova, Georgia, Kyrgyzstan, Uzbekistan, Tajikistan and Turkmenistan.(25, 31) We also did not find any HBsAg positive cases among those 70 years and older, while the percentage positive for anti-HBc was highest in this group. On one hand, those with persistent chronic infection might have already had a lethal outcome at that age, on other hand, the sample size in this age group (n=274) might have been a limiting factor to detect prevalent cases. Furthermore, the HBsAg clearance rate with and without therapy might also influence this figure.(32) Several other factors associated with higher odds for current

For peer review only

or past infection were identified in the multivariable regression models. For both being ever infected and chronic infection, odds were higher among people with less than secondary education and current smokers. Odds for being ever infected were higher among persons living in *marzes* (versus Yerevan), those demonstrating poorer knowledge on infectious diseases, and those ever imprisoned. Individuals with lower levels of education, smoking, and individuals who have been imprisoned might be more likely to engage in riskier behavior such as drug use and unprotected sexual activity leading to increased exposure to HBV.(33-35) Furthermore, lower education levels and socioeconomic disadvantages can result in reduced access to healthcare services and prevention.(36, 37) Higher odds for ever being infected with hepatitis B in *marzes* compared to Yerevan could be as well related to historically poorer access to health care or lower level of sanitary-epidemiological measures in regional heath care centers. Though, there is no documented evidence supporting this assumption. We could also demonstrate that poorer knowledge about infectious diseases was associated with higher odds for detection of anti-HBc, possibly explained by poorer knowledge about transmission routes, limited understanding of preventive measures, misconceptions and stigma and poorer access to health related information.(35, 38, 39) Findings on associated factors suggest that information campaigns and prevention, especially among groups having higher risk for infection due to behavioral or socio-economic reasons, are needed to further decrease hepatitis B incidence.

In 7.9% of chronic infections (n=2 in the weighted analysis), the viral load was above 10,000 IU/ml, indicating the need for treatment.(40) A viral load above 10,000 IU/ml is a strong risk predictor of HCC, independent of HBeAg status, ALT level and liver cirrhosis.(41-45) The results on viral load suggest that there is a tangible proportion of chronic hepatitis B patients in Armenia that are currently not receiving antiviral treatment or were not aware of their infection. This is supported by the fact that only 22% of those testing positive for HBsAg reported that they have ever been told by a doctor that they were infected with hepatitis B, thus, were not diagnosed

For peer review only - http://bmjopen.bmj.com/site/about/guidelines.xhtml

previously. As the testing and treatment costs in case of a chronic hepatitis B infection are currently mainly related to out-of-pocket payments in Armenia, the detection of cases and sustaining them in care is hindered.(26). Improvement of state-funded preventive and testing strategies and warranting access to state-funded hepatitis B treatment is needed in order to find and timely treat cases to prevent sequel like cirrhosis and HCC, and decrease mortality.

Based on our prevalence findings, the estimated number of people living with chronic HBV infection in Armenia could be around 23,000, and 1,700 of them could be at high risk of HCC and need immediate treatment. Yet, roughly, only one-fifth of chronically infected are aware of their infection. The WHO target for 2030 is 90% of people living with hepatitis B diagnosed – calling for improved access to testing to reach the global and regional goals.(46) Hence, there is a need to revise testing and treatment strategies in Armenia in order to find and timely treat chronic hepatitis B cases to prevent sequel and reduce mortality.

Limitations: Due to limited length of the questionnaire we were not able to include all relevant questions to determine other factors associated with higher odds of being infected with HBV. Furthermore, as the sample size was calculated for the Sars-CoV-2 study needs, our possibilities for calculation of prevalence of HBV infection for different groups was limited and we might lack power to detect differences among groups and when performing regression analyses. At the same time, the sample size was appropriate to measure nationwide robust estimates with narrow confidence intervals.

CONCLUSIONS

This is the first nation-wide representative study in Armenia providing a robust estimate of anti-HBc and HBsAg prevalence among general population. While the prevalence of chronic hepatitis B is rather low in Armenia, the proportion of persons ever been infected with hepatitis B is considerable. Age distribution of HBV infection ever in life suggests that there has been

For peer review only - http://bmjopen.bmj.com/site/about/guidelines.xhtml

historically high incidence and that new infections possibly still occur among adults. Meanwhile, the undetectable prevalence among younger age groups suggests the positive impact of the hepatitis B vaccination.

In order to find and treat HBV infected persons, testing should be scaled up in first line considering population groups that are likely more affected. Furthermore, to reach elimination of HBV, prevention of mother-to-child transmission should be maintained at high level.(47) Validation of reaching hepatitis B control targets with a study targeting younger children (from the vaccinated cohort) could be considered.

Furthermore, prevention among other indication groups, like, for example, trough vaccination of risk groups and access to needle-syringe programs, should be in place and at sufficient coverage. Actions to improve knowledge on hepatitis B transmission routs, prevention and testing are needed for all population groups.

Acknowledgements This work was done with partial support from WHO Europe and technical assistance from Robert Koch Institute. The authors alone are responsible for the views expressed in this article and they do not necessarily represent the views, decisions, or policies of the institutions with which they are affiliated.

Contributors AD, SS, LM, AM, NS, GG and VP conceptualized this study, AD performed the analysis. AD, SD, LA, and DM drafted the manuscript. All authors supported the interpretation of the study results and critically reviewed and revised the manuscript.

Competing interests None declared.

Funding The data collection was partially funded by WHO Europe (no grant number). No funding was received for developing this manuscript.

Data availability statement Data are available on reasonable request.

For peer review only - http://bmjopen.bmj.com/site/about/guidelines.xhtml

REFERENCES

1. G. B. D. Hepatitis B Collaborators. Global, regional, and national burden of hepatitis B, 1990-2019: a systematic analysis for the Global Burden of Disease Study 2019. Lancet Gastroenterol Hepatol. 2022;7(9):796-829. doi: 10.1016/S2468-1253(22)00124-8

2. Purcell RH. The discovery of the hepatitis viruses. Gastroenterology. 1993;104(4):955-63. doi: 10.1016/0016-5085(93)90261-a

3. Tseng TC, Liu CJ, Yang HC, et al. High levels of hepatitis B surface antigen increase risk of hepatocellular carcinoma in patients with low HBV load. Gastroenterology. 2012;142(5):1140-9 e3; quiz e13-4. doi: 10.1053/j.gastro.2012.02.007

4. Hou J, Liu Z, Gu F. Epidemiology and Prevention of Hepatitis B Virus Infection. Int J Med Sci. 2005;2(1):50-7. doi: 10.7150/ijms.2.50

5. Zhao X, Shi X, Lv M, et al. Prevalence and factors associated with hepatitis B virus infection among household members: a cross-sectional study in Beijing. Hum Vaccin Immunother. 2021;17(6):1818-24. doi: 10.1080/21645515.2020.1847951

6. Karoney MJ, Some FF, Dip HIL, et al. Burden of Hepatitis B Infection among High Risk Populations in Western Kenya. J Infect Dis Epidemiol 2020;6:132. doi: 10.23937/2474-3658/1510132

7. Muljono DH, Wijayadi T, Sjahril R. Hepatitis B Virus Infection among Health Care Workers in Indonesia. Euroasian J Hepatogastroenterol. 2018;8(1):88-92. doi: 10.5005/jp-journals-10018-1269

8. Gnyawali B, Pusateri A, Nickerson A, et al. Epidemiologic and socioeconomic factors impacting hepatitis B virus and related hepatocellular carcinoma. World J Gastroenterol. 2022;28(29):3793-802. doi: 10.3748/wjg.v28.i29.3793

For peer review only - http://bmjopen.bmj.com/site/about/guidelines.xhtml

9. Tosun S, Aygun O, Ozdemir HO, et al. The impact of economic and social factors on the prevalence of hepatitis B in Turkey. BMC Public Health. 2018;18(1):649. doi: 10.1186/s12889-018-5575-6

10. Lok AS, McMahon BJ. Chronic hepatitis B. Hepatology. 2007;45(2):507-39. doi: 10.1002/hep.21513

11. Hyams KC. Risks of Chronicity following Acute Hepatitis B Virus Infection: A Review. Clin Infect Dis. 1995;20(4):992-1000. doi: 10.1093/clinids/20.4.992

12. Pattyn J, Hendrickx G, Vorsters A, et al. Hepatitis B Vaccines. J Infect Dis. 2021;224(12 Suppl 2):S343-S51. doi: 10.1093/infdis/jiaa668

13. Ott JJ, Stevens GA, Groeger J, et al. Global epidemiology of hepatitis B virus infection: new estimates of age-specific HBsAg seroprevalence and endemicity. Vaccine. 2012;30(12):2212-9. doi: 10.1016/j.vaccine.2011.12.116

14. Andreasyan D, Bazarchyan A. "Health and Health Care" Yearbook. Yerevan: National Institute of Health Named after Academician S. Avdalbekyan; 2022. 299 p.

15. Demirchyan A, Mirzoyan L, Thompson ME. Synthesis of the existing data on Hepatitis B in Armenia. Yerevan: American University of Armenia, Center for Health Services Research; 2000. https://aua.am/chsr/PDF/2000/HepatitB_FINAL.pdf

16. Ghazinyan H, Mkhitaryan A, Melik-Andreasyan G, et al. Updating HBV status in Armenia Optimal Management of Hepatitis B Virus Infection; 25–27 September Athens, Greece: Proceedings of EASL Special Conference; 2014. p. 159.

17. Melik-Andreasyan GG, Markosyan IE, Voskanyan LS, et al. Epidemiological Characteristics of Viral Hepatitis in Armenia (in Russian). В мире вирусных гепатитов. 2013;4:20-6.

For peer review only - http://bmjopen.bmj.com/site/about/guidelines.xhtml

18. Davlidova S, Haley-Johnson Z, Nyhan K, et al. Prevalence of HIV, HCV and HBV in Central Asia and the Caucasus: A systematic review. Int J Infect Dis. 2021;104:510-25. doi: 10.1016/j.ijid.2020.12.068

19. Roche Diagnostics. Elecsys® Anti-HBc II. Immunoassay for the qualitative determination of total antibodies against hepatitis B core antigen (HBcAg) [Available from: https://diagnostics.roche.com/global/en/products/params/elecsys-anti-hbc-ii.html].

20. Roche Diagnostics. Elecsys® HBsAg II. Immunoassay for the qualitative determination of hepatitis B surface antigen (HBsAg) [Available from: https://diagnostics.roche.com/global/en/products/params/elecsys-hbsag-ii.html].

21. Hosmer DW, Lemeshow S. Applied logistic regression. 2nd ed. New York: A Wiley-Interscience Publication; 2000: 92-116.

22. Khetsuriani N, Gamkrelidze A, Shadaker S, et al. Hepatitis B prevalence and the impact of vaccination in Georgia: results from a nationwide serosurvey International Liver Congress TM 2022, London22-26 June 2022 [Available from: https://www.postersessiononline.eu/173580348_eu/congresos/ILC2022/aula/-THU_283_ILC2022.pdf].

23. Polaris Observatory C. Global prevalence, treatment, and prevention of hepatitis B virus infection in 2016: a modelling study. Lancet Gastroenterol Hepatol. 2018;3(6):383-403. doi: 10.1016/S2468-1253(18)30056-6

24. Coalition for Global Hepatitis Elimination. Data Dashboard Armenia. [Available from: https://www.globalhep.org/country-progress/armenia] (accessed 27 July 2023).

25. Khetsuriani N, Tishkova F, Jabirov S, et al. Substantial decline in hepatitis B virus infections following vaccine introduction in Tajikistan. Vaccine. 2015;33(32):4019-24. doi: 10.1016/j.vaccine.2015.05.092

For peer review only - http://bmjopen.bmj.com/site/about/guidelines.xhtml

26. Robert Koch Institute. Report: Technical Assessment of Viral Hepatitis Response in Armenia. Berlin; 2023: 16-19.

27. Khan NU, Zalan A, Petruzziello A, et al. Determining the Actual Prevalence of Hepatitis B in Khyber Pakhtunkhwa-Pakistan: A Meta-Analysis. Open Virol J. 2018;12:33-41. doi: 10.2174/1874357901812010033

28. Wang H, Men P, Xiao Y, et al. Hepatitis B infection in the general population of China: a systematic review and meta-analysis. BMC Infect Dis. 2019;19(1):811. doi: 10.1186/s12879-019-4428-y

29. Brown R, Goulder P, Matthews PC. Sexual Dimorphism in Chronic Hepatitis B Virus (HBV) Infection: Evidence to Inform Elimination Efforts. Wellcome Open Res. 2022;7:32. doi: 10.12688/wellcomeopenres.17601.2

30. Ruggieri A, Gagliardi MC, Anticoli S. Sex-Dependent Outcome of Hepatitis B and C Viruses Infections: Synergy of Sex Hormones and Immune Responses? Front Immunol. 2018;9:2302. doi: 10.3389/fimmu.2018.02302

31. More countries reaching hepatitis B control targets brings the WHO European Region closer to eliminating viral hepatitis as a public health threat [press release]. https://www.who.int/europe/news/item/24-04-2023-more-countries-reaching-hepatitis-b-control-targets-brings-the-who-european-region-closer-to-eliminating-viral-hepatitis-as-a-public-health-threat2023 (accessed 25 April 2023).

32. Terrault NA, Wahed AS, Feld JJ, et al. Incidence and prediction of HBsAg seroclearance in a prospective multi-ethnic HBeAg-negative chronic hepatitis B cohort. Hepatology. 2022;75(3):709-23. doi: 10.1002/hep.32231

33. Galea S, Vlahov D. Social determinants and the health of drug users: socioeconomic status, homelessness, and incarceration. Public Health Rep. 2002;117 Suppl 1(Suppl 1):S135-45.

For peer review only

For peer review only - http://bmjopen.bmj.com/site/about/guidelines.xhtml

34. MacLachlan JH, Cowie BC. Hepatitis B virus epidemiology. Cold Spring Harb Perspect Med. 2015;5(5):a021410. doi: 10.1101/cshperspect.a021410

35. Corona R, Caprilli F, Giglio A, et al. Risk factors for hepatitis B virus infection among heterosexuals attending a sexually transmitted diseases clinic in Italy: role of genital ulcerative diseases. J Med Virol. 1996;48(3):262-6. doi: 10.1002/(SICI)1096-9071(199603)48:3<262::AID-JMV8>3.0.CO;2-6

36. McMaughan DJ, Oloruntoba O, Smith ML. Socioeconomic Status and Access to Healthcare: Interrelated Drivers for Healthy Aging. Front Public Health. 2020;8:231. doi: 10.3389/fpubh.2020.00231

37. Brodzinski A, Neumeyer-Gromen A, Dudareva S, et al. [Hepatitis B virus infection and vaccine-induced immunity: the role of sociodemographic determinants: Results of the study "German Health Interview and Examination Survey for Adults" (DEGS1, 2008-2011)]. Bundesgesundheitsblatt Gesundheitsforschung Gesundheitsschutz. 2022;65(2):159-69. doi: 10.1007/s00103-021-03473-z

38. Machmud PB, Glasauer S, Gottschick C, et al. Knowledge, Vaccination Status, and Reasons for Avoiding Vaccinations against Hepatitis B in Developing Countries: A Systematic Review. Vaccines (Basel). 2021;9(6). doi: 10.3390/vaccines9060625

39. Pereira LM, Martelli CM, Merchan-Hamann E, et al. Population-based multicentric survey of hepatitis B infection and risk factor differences among three regions in Brazil. Am J Trop Med Hyg. 2009;81(2):240-7.

40. European Association for the Study of the Liver. EASL 2017 Clinical Practice Guidelines on the management of hepatitis B virus infection. J Hepatol. 2017;67(2):370-98. doi: 10.1016/j.jhep.2017.03.021

For peer review only

41. Chen CJ, Yang HI, Su J, et al. Risk of hepatocellular carcinoma across a biological gradient of serum hepatitis B virus DNA level. JAMA. 2006;295(1):65-73. doi: 10.1001/jama.295.1.65

42. Liu CJ, Chen BF, Chen PJ, et al. Role of hepatitis B viral load and basal core promoter mutation in hepatocellular carcinoma in hepatitis B carriers. J Infect Dis. 2006;193(9):1258-65. doi: 10.1086/502978

43. Barcena Marugan R, Garcia Garzon S. DNA-guided hepatitis B treatment, viral load is essential, but not sufficient. World J Gastroenterol. 2009;15(4):423-30. doi: 10.3748/wjg.15.423

44. Mendy ME, Welzel T, Lesi OA, et al. Hepatitis B viral load and risk for liver cirrhosis and hepatocellular carcinoma in The Gambia, West Africa. J Viral Hepat. 2010;17(2):115-22. doi: 10.1111/j.1365-2893.2009.01168.x

45. Kim GA, Han S, Choi GH, et al. Moderate levels of serum hepatitis B virus DNA are associated with the highest risk of hepatocellular carcinoma in chronic hepatitis B patients. Aliment Pharmacol Ther. 2020;51(11):1169-79. doi: 10.1111/apt.15725

46. World Health Organization. Global Hepatitis Report. Geneva: Licence: CC BY-NC-SA 3.0 IGO.; 2017. https://apps.who.int/iris/bitstream/handle/10665/255016/9789241565455-eng.pdf?sequence=1&isAllowed=y

47. Polaris Observatory, C., Global prevalence, cascade of care, and prophylaxis coverage of hepatitis B in 2022: a modelling study. Lancet Gastroenterol Hepatol, 2023. Doi: 10.1016/S2468-1253(23)00197-8

For peer review only - http://bmjopen.bmj.com/site/about/guidelines.xhtml

STROBE Statement—Checklist of items that should be included in reports of ***cross-sectional studies***

	Item No	Recommendation	Page No
**Title and abstract**	1	(*a*) Indicate the study's design with a commonly used term in the title or the abstract	1
		(*b*) Provide in the abstract an informative and balanced summary of what was done and what was found	1
**Introduction**			
Background/rationale	2	Explain the scientific background and rationale for the investigation being reported	3-4
Objectives	3	State specific objectives, including any prespecified hypotheses	5
**Methods**			
Study design	4	Present key elements of study design early in the paper	5-6
Setting	5	Describe the setting, locations, and relevant dates, including periods of recruitment, exposure, follow-up, and data collection	5-6
Participants	6	(*a*) Give the eligibility criteria, and the sources and methods of selection of participants	6
Variables	7	Clearly define all outcomes, exposures, predictors, potential confounders, and effect modifiers. Give diagnostic criteria, if applicable	7-8
Data sources/ measurement	8*	For each variable of interest, give sources of data and details of methods of assessment (measurement). Describe comparability of assessment methods if there is more than one group	*7-8*
Bias	9	Describe any efforts to address potential sources of bias	6-7
Study size	10	Explain how the study size was arrived at	6
Quantitative variables	11	Explain how quantitative variables were handled in the analyses. If applicable, describe which groupings were chosen and why	9
Statistical methods	12	(*a*) Describe all statistical methods, including those used to control for confounding	9-10
		(*b*) Describe any methods used to examine subgroups and interactions	NA
		(*c*) Explain how missing data were addressed	10
		(*d*) If applicable, describe analytical methods taking account of sampling strategy	9
		(*e*) Describe any sensitivity analyses	NA
**Results**			
Participants	13*	(a) Report numbers of individuals at each stage of study—eg numbers potentially eligible, examined for eligibility, confirmed eligible, included in the study, completing follow-up, and analysed	10
		(b) Give reasons for non-participation at each stage	NA
		(c) Consider use of a flow diagram	NA
Descriptive data	14*	(a) Give characteristics of study participants (eg demographic, clinical, social) and information on exposures and potential confounders	11-14
		(b) Indicate number of participants with missing data for each variable of interest	11-14
Outcome data	15*	Report numbers of outcome events or summary measures	10-11

For peer review only - http://bmjopen.bmj.com/site/about/guidelines.xhtml

Main results	16	(*a*) Give unadjusted estimates and, if applicable, confounder-adjusted estimates and their precision (eg, 95% confidence interval). Make clear which confounders were adjusted for and why they were included	10
		(*b*) Report category boundaries when continuous variables were categorized	8
		(*c*) If relevant, consider translating estimates of relative risk into absolute risk for a meaningful time period	NA
Other analyses	17	Report other analyses done—eg analyses of subgroups and interactions, and sensitivity analyses	NA
**Discussion**			
Key results	18	Summarise key results with reference to study objectives	17
Limitations	19	Discuss limitations of the study, taking into account sources of potential bias or imprecision. Discuss both direction and magnitude of any potential bias	20
Interpretation	20	Give a cautious overall interpretation of results considering objectives, limitations, multiplicity of analyses, results from similar studies, and other relevant evidence	20-21
Generalisability	21	Discuss the generalisability (external validity) of the study results	18
**Other information**			
Funding	22	Give the source of funding and the role of the funders for the present study and, if applicable, for the original study on which the present article is based	21

*Give information separately for exposed and unexposed groups.

Note: An Explanation and Elaboration article discusses each checklist item and gives methodological background and published examples of transparent reporting. The STROBE checklist is best used in conjunction with this article (freely available on the Web sites of PLoS Medicine at http://www.plosmedicine.org/, Annals of Internal Medicine at http://www.annals.org/, and Epidemiology at http://www.epidem.com/). Information on the STROBE Initiative is available at www.strobe-statement.org.

For peer review only

For peer review only - http://bmjopen.bmj.com/site/about/guidelines.xhtml

BMJ Open

Prevalence of hepatitis B virus infection among general population of Armenia in 2021 and factors associated with it: a cross-sectional study

Journal:	*BMJ Open*
Manuscript ID	bmjopen-2023-080281.R1
Article Type:	Original research
Date Submitted by the Author:	11-Dec-2023
Complete List of Authors:	Demirchyan, Anahit; American University of Armenia, Turpanjian College of Health Sciences<br>Dudareva, S.; Robert Koch Institut, Department for Infectious Disease Epidemiology<br>Sahakyan, Serine; American University of Armenia, Turpanjian College of Health Sciences<br>Aslanyan, Lusine; American University of Armenia, Turpanjian College of Health Sciences<br>Muradyan, Diana; American University of Armenia, Turpanjian College of Health Sciences<br>Musheghyan, Lusine; American University of Armenia, Turpanjian College of Health Sciences<br>Mozalevskis, Antons; World Health Organization, Global HIV, Hepatitis and Sexually Transmitted Infections Programmes<br>Sargsyants, Narina; National Institute of Health named after academician Suren Avdalbekyan<br>Ghukasyan, Gayane; World Health Organization Country Office in Armenia<br>Petrosyan, V; American University of Armenia, Turpanjian College of Health Sciences
**Primary Subject Heading**:	Epidemiology
Secondary Subject Heading:	Infectious diseases
Keywords:	Epidemiology < INFECTIOUS DISEASES, Infection control < INFECTIOUS DISEASES, Public health < INFECTIOUS DISEASES

SCHOLARONE™
Manuscripts

I, the Submitting Author has the right to grant and does grant on behalf of all authors of the Work (as defined in the below author licence), an exclusive licence and/or a non-exclusive licence for contributions from authors who are: i) UK Crown employees; ii) where BMJ has agreed a CC-BY licence shall apply, and/or iii) in accordance with the terms applicable for US Federal Government officers or employees acting as part of their official duties; on a worldwide, perpetual, irrevocable, royalty-free basis to BMJ Publishing Group Ltd ("BMJ") its licensees and where the relevant Journal is co-owned by BMJ to the co-owners of the Journal, to publish the Work in this journal and any other BMJ products and to exploit all rights, as set out in our licence.

The Submitting Author accepts and understands that any supply made under these terms is made by BMJ to the Submitting Author unless you are acting as an employee on behalf of your employer or a postgraduate student of an affiliated institution which is paying any applicable article publishing charge ("APC") for Open Access articles. Where the Submitting Author wishes to make the Work available on an Open Access basis (and intends to pay the relevant APC), the terms of reuse of such Open Access shall be governed by a Creative Commons licence – details of these licences and which Creative Commons licence will apply to this Work are set out in our licence referred to above.

Other than as permitted in any relevant BMJ Author's Self Archiving Policies, I confirm this Work has not been accepted for publication elsewhere, is not being considered for publication elsewhere and does not duplicate material already published. I confirm all authors consent to publication of this Work and authorise the granting of this licence.

For peer review only

Prevalence of hepatitis B virus infection among general population of Armenia in 2021 and factors associated with it: a cross-sectional study

Demirchyan, Anahit[1*]; Dudareva, Sandra[2]; Sahakyan, Serine[1]; Aslanyan, Lusine[1]; Muradyan, Diana[1]; Musheghyan, Lusine[1]; Mozalevskis, Antons[3]; Sargsyants, Narina[4]; Ghukasyan, Gayane[5]; Petrosyan, Varduhi[1]

[1] Turpanjian College of Health Sciences, American University of Armenia, Yerevan, Armenia

[2] Department for Infectious Disease Epidemiology, Robert Koch Institute, Berlin, Germany

[3] Global HIV, Hepatitis and Sexually Transmitted Infections Programmes, World Health Organization, Geneva, Switzerland

[4] National Institute of Health, Ministry of Health, Republic of Armenia, Yerevan, Armenia

[5] World Health Organization Country Office in Armenia, Yerevan, Armenia

[*] Corresponding author: Demirchyan, Anahit. Address: 40 Marshal Baghramyan ave., Yerevan 0019, Armenia. e-mail: ademirch@aua.am, telephone: +374 60 612562, Fax: +374 60 612512

Keywords: Hepatitis B virus infection; seroprevalence; Armenia; general adult population.

Word count: 4,500

For peer review only

For peer review only - http://bmjopen.bmj.com/site/about/guidelines.xhtml

Abstract

Objectives This study sought to determine the prevalence and associated factors of Hepatitis B virus (HBV) infection ever in life and chronic HBV infection in Armenia.

Design A population-based cross-sectional seroprevalence study combined with a phone survey of tested individuals.

Setting All administrative units of Armenia including ten provinces and capital city Yerevan.

Participants The study frame was the general adult population of Armenia aged ≥18 years.

Primary and secondary outcome measures The participants were tested for anti-HBV core antibodies (anti-HBc) and HBV surface antigen (HBsAg) using third-generation enzyme immunoassays. In case of HBsAg positivity, HBV DNA and hepatitis D virus (HDV) RNA PCR tests were performed. Risk factors of HBV infection ever in life (anti-HBc positivity) and chronic HBV infection (HBsAg positivity) were identified through fitting logistic regression models.

Results The seroprevalence study included 3838 individuals 18 years and older. Of them, 90.7% (3476 individuals) responded to the phone survey. The prevalence of anti-HBc positivity was 14.1% (95% CI 13.1%-15.2%) and HBsAg positivity 0.8% (95% CI 0.5%-1.1%). The viral load was over 10,000 IU/ml for 7.9% of HBsAg-positive individuals. None of the participants was positive for HDV. Risk factors for HBsAg positivity included less than secondary education (aOR=6.44; 95% CI 2.2-19.1), current smoking (aOR=2.56; 95% CI 1.2-5.6), and chronic liver disease (aOR=8.44; 95% CI 3.0-23.7). In addition to these, risk factors for anti-HBc positivity included age (aOR=1.04; 95% CI 1.04-1.05), imprisonment ever in life (aOR=2.53; 95% CI 1.41-4.56), and poor knowledge on infectious diseases (aOR=1.32; 95% CI 1.05-1.67), while living in Yerevan (vs. provinces) was protective (aOR=0.74; 95% CI 0.59-0.93).

Conclusion This study provided robust estimates of HBV markers among general population of Armenia. Its findings delineated the need to revise HBV testing and treatment strategies considering higher risk population groups, and improve population knowledge on HBV prevention.

For peer review only - http://bmjopen.bmj.com/site/about/guidelines.xhtml

STRENGTHS AND LIMITATIONS OF THIS STUDY

⇒ Rigorous sampling methodology applied throughout Armenia insured the generalisability of the findings.

⇒ Large sample size allowed to obtain robust prevalence estimates with narrow confidence intervals.

⇒ Items on some important behavioral risk factors of HBV infection were not included in the questionnaire because of their sensitive nature.

⇒ The study methodology did not allow targeting specific population groups at high risk for HBV infection.

For peer review only

For peer review only - http://bmjopen.bmj.com/site/about/guidelines.xhtml

INTRODUCTION

Despite the availability of effective vaccination against hepatitis B virus (HBV), it continues to be a global threat to public health, particularly in low- and middle-income countries (LMIC).(1) According to World Health Organization (WHO) 2019 data, 296 million people in the globe live with chronic hepatitis B, and 1.5 million new infections occur annually.(1) More than a million fatalities globally are attributed to HBV, which is the primary cause of morbidity and mortality from chronic hepatitis, cirrhosis, and hepatocellular carcinoma (HCC), in addition to the acute infection itself.(2, 3)

The commonly known risk factors for HBV are being born to an infected mother, sharing injection syringes and needles, men having sex with men (MSM), female sex workers, living with HIV, and having occupational exposure to blood and blood products.(4-7) Additionally, studies have shown that sociodemographic factors including older age, male sex, low socio-economic status, poor housing, and low education could be risk factors for chronic HBV infection.(8, 9)

There has been a decline in the prevalence of chronic HBV infection in many parts of the world since a reliable vaccine became widely accessible more than 20 years ago.(10) Improved medical practices, such as screening of blood and blood products, applying safe injection techniques, and implementing infection control policies further contributed to this decline.(11)

A noticeable decrease in the incidence of acute viral hepatitis infections was observed in Armenia over the last three decades. The incidence of acute HBV infection steadily decreased from 22.30 in 1990 to 0.17 in 2021 per 100,000 population.(12) One of the explanations of this decrease is the high (over 96%) coverage of hepatitis B newborn and infant vaccination introduced in Armenia in 1999.(13)

Concerning the prevalence of chronic HBV infection, no nationally representative seroprevalence studies of HBV markers were conducted among general population of Armenia

For peer review only - http://bmjopen.bmj.com/site/about/guidelines.xhtml

before 2021. Based on synthesis of available screening data on blood donors, pregnant women, healthcare providers, and patients, the best guess was that chronic HBV infection was around 2% among general population of Armenia in 2000.(13) In 2013, based on retrospective testing of frozen blood samples of over 10,000 healthy individuals aged from 1 to 60 years, the median prevalence of HBV surface antigen (HBsAg) was 1.9-2.5%.(14, 15) In the meantime, the prevalence was reported as 1.4% among blood donors, 1.5% among pregnant women, 4.3% among prisoners, 6.6% among hemodialysis patients, 7.3% among healthcare workers, 8.8% among people who inject drugs, and 16.0% among patients with sexually transmitted infections.(15)

A recent review study that explored the groups of population at higher risk for chronic HBV infection in the countries of Caucasus and Central Asia, stated that chronic HBV infection was higher (3.7%) among prisoners in Armenia.(16) Concerning other vulnerable groups, the estimates varied across different small-scale studies from 0% to 1.6% for MSM and from 0.3% to 0.9% for migrants (mainly seasonal workers returning from Russia).(16)

For the purpose of assessing national disease prevention and control initiatives including immunization programs, knowledge of the region- and age-specific prevalence of hepatitis B infection is essential. The study presented in this paper was the first attempt to identify the seroprevalence of HBV infection among general adult population of Armenia. The study aimed to determine the prevalence of HBV infection ever in life and chronic HBV infection in Armenia and identify the risk factors of each of these outcomes among the general adult population.

METHODS

Study design

This study was a cross-sectional population-based sero-epidemiological study conducted throughout Armenia in May-September, 2021. The study included laboratory testing of blood

For peer review only - http://bmjopen.bmj.com/site/about/guidelines.xhtml

serum samples of the participants for biomarkers of hepatitis B, hepatitis C, and SARS-CoV-2 viruses, which was shortly followed with a telephone survey among them. The study was primarily designed to measure the biomarkers for SARS-CoV-2 and the hepatitis markers were added to it. The current paper reflects the analytical approaches and findings on HBV infection.

Sampling and sample size

The study frame was the general adult population of Armenia aged 18 years and over. We used a proportionate-to-the-population-size cluster sampling design, which included all 11 administrative units of Armenia (the ten provinces and Yerevan city). The study participants were recruited from primary healthcare (PHC) facilities, as the coverage of population with PHC is quite high in Armenia (about 97%). We selected one-third of the polyclinics in each administrative unit via proportionate to the served population size random sampling. Simple random sampling was then applied in the national e-health operator "ARMED" database to select the study participants from the lists of 18 years old and older individuals served by each chosen polyclinic.

The formula for estimating a proportion ($n = N*X / (X + N – 1)$, where $X = Z_{\alpha/2}^2 *p*(1-p) / (MOE)^2$) of the population was used to calculate the sample size for this study. When assuming 30% prevalence of anti-SARS-CoV-2 antibodies, 1.45% margin of error (MOE), type 1 error of 0.05, and confidence level of 95%, the calculated sample size was 3832. Assuming expected HBsAg prevalence of 2% and HBV core antibody (Anti-HBc) prevalence of 15%, this sample size allowed calculation of HBsAg prevalence estimate with a MOE of 0.44% and Anti-HBc prevalence estimate with a MOE of 1.13%.

Data collection

The "ARMED" e-health system provided the list of unique ID numbers selected through simple random sampling to the respective polyclinic's personnel trained for contacting the study

For peer review only - http://bmjopen.bmj.com/site/about/guidelines.xhtml

participants. The personnel contacted each potential participant by phone, following the script for the first contact, introduced the study and invited her/him to the polyclinic to take part in the study. When the participants came to the PHC facility, the research team obtained a written informed consent and collected blood samples. The participant recruitment in each polyclinic continued until the needed sample size was reached. Blood sampling was done by a trained nurse. Telephone interviews were carried out by trained interviewers within two weeks after the blood sampling. The structured questionnaire was administered using electronic tablets where Alchemer online tool was installed.

The study protocol was reviewed and approved by the Ethics Review Committee of the World Health Organization (protocol ID: CERC.0013C) and the Institutional Review Board of the American University of Armenia (protocol ID: AUA-2021-005), as conforming to the principles embodied in the Declaration of Helsinki. If the test result was positive, a trained hepatologist communicated the result to the individual and provided counseling. Negative results were sent to the participants via SMS messages.

Serum tests and definitions

Via venipuncture, 5-10 ml venous blood was collected from each participant and centrifuged immediately for the serum extraction. The serums were kept at 2-8°C for seven days at most before being tested at the laboratory of the National Center for Infectious Diseases of the Republic of Armenia. This laboratory was chosen because of having the needed technical and human resources to perform third generation serological and molecular testing with the required quality for all three infections included in the study. The testing procedure for HBV included the following steps: (1) all samples were tested for total anti-HBc antibodies using third-generation enzyme immunoassay (Elecsys® Anti-HBc II-Roche Diagnostics with over 99.3% clinical specificity and 100% sensitivity (17)); (2) if anti-HBc antibodies were detected, HBsAg test was performed (using Elecsys HBsAg-Roche Diagnostics with over 99.8% clinical specificity and

For peer review only

99.9% sensitivity (18)), followed by a confirmatory test if the result was questionable (within 0.9-1.0 COI); (3) if HBsAg test was positive, HBV DNA test was performed to test for the presence of HBV DNA and the viral load (using Amplisens HBV Monitor FL (detection limit 150 IU/ml)); (4) all samples positive for HBsAg were also tested for hepatitis D virus (HDV) RNA (PCR test using Amplisens HDV-FL (detection limit 100 copies/ml)). All the applied tests were WHO pre-qualified. The presence of antibodies against HBV core antigen (anti-HBc antibodies) was viewed as serological evidence of HBV infection ever in life, while the presence of HBV surface antigen (HBsAg[1]) served as serological evidence of chronic HBV infection.

Survey instrument and study variables

The two outcome variables of this study were anti-HBc antibody status and HBsAg status measuring HBV infection ever in life and chronic HBV infection, respectively. The survey tool was a structured questionnaire which included items related to this study grouped in socioeconomic, health status, health literacy, and health behavioral domains. The sociodemographic domain included three continuous (age, family size, and socioeconomic status (SES) score) and four categorical (sex, education, employment, and residence) variables. The health status domain included only categorical (dichotomous) variables: hepatitis C exposure and chronic infection, several other self-reported chronic conditions (diabetes, chronic liver disease, obesity, heart disease, cancer, chronic kidney disease, and asthma requiring medication), as well as symptoms experienced during the six months prior to the survey (vomiting, fatigue, loss of appetite, joint ache, abdominal pain, fever, and nose bleeding). The health literacy domain included a communicable diseases knowledge variable measured by four questions and dichotomized at its lower third, and a score measuring the difficulty of understanding health-related information generated from responses to ten items. The health behavioral domain

[1] The WHO surveillance case definition for chronic HBV infection assumes the presence of biomarkers of the infection combined with the absence of signs and symptoms of acute hepatitis. We applied this definition as coming across a recent infection in a cross-sectional population study is unlikely.

For peer review only - http://bmjopen.bmj.com/site/about/guidelines.xhtml

included the following categorical variables: ever and current regular smoking, having tattoos, ever having been imprisoned, ever receiving blood transfusion, frequency of visiting a dentist, and self-reported hepatitis B vaccination status. A variable on ever having been told by a doctor of being infected with HBV was also included in the analysis. The questionnaire is provided as a supplementary file.

Patient and public involvement statement

The survey questionnaire was pretested among eligible people and refined based on their feedback. This was the only involvement of public in this research.

Data analysis

We used IBM SPSS Statistics for Windows, version 21 (IBM Corp., Armonk, N.Y., USA) for data management and analysis. All the analysis was weighted by sex and 5-year age groups to closely mimic age and sex distribution of the population of Armenia, as age and sex distribution in the study sample differed from that in the general population due to differences in response rates of different age and sex groups. To estimate the prevalence of HBV infection ever in life and chronic HBV infection, proportions and 95% confidence intervals were calculated for positivity to anti-HBc and HBsAg, respectively. To descriptively analyze independent variables, frequencies and proportions were calculated for categorical variables, and means and standard deviations for continuous variables in the total sample and in each of the outcome groups differentiated based on positivity/negativity to anti-HBc and positivity/negativity to HBsAg. Then univariate logistic regression analysis was done. The linearity of the association of continuous variables with each outcome variable was checked on the logit scale before entering them into logistic regression analysis.(19) The final step was fitting logistic regression models to identify factors independently associated to each of the outcomes. A series of multivariable logistic regression analyses were performed with inclusion of all those variables associated with

For peer review only - http://bmjopen.bmj.com/site/about/guidelines.xhtml

the outcome at the $p < 0.25$ level in the univariate analysis.(19) The variables were entered into logistic regression analysis in different combinations using e*nter* method. The variables not related to the outcome ($p \geq 0.10$) when controlling for the remaining variables in the model were excluded from the model, if their removal did not change the associations between the remaining variables and the outcome. The decision on keeping variables with a p-value between 0.05 and 0.10 in the model was made based on their potential relevance for interpretation of the data. The final models were checked for model fit and collinearity. Cases with any missing values were excluded from the fitted models.

RESULTS

Trained personnel from the selected polyclinics contacted a total of 9009 eligible individuals throughout the country to invite them for blood testing. Of them, 3831 (42.5%) individuals agreed and underwent blood testing. Of those tested, 3476 individuals (90.7%) participated in the phone survey. The prevalence numbers of HBV markers were generated from the sample that was tested (n=3831), but the variables needed for the rest of the analysis (descriptive comparisons and logistic regression) were available only for those 3476 individuals who participated in the phone survey and answered the survey questionnaire. Of them, 54.5% were women and 45.5% men. The mean age of women was 46.8 years (SD 17.4), and the mean age of men 44.6 years (SD 17.1). Of men, 43.9%, and of women, 41.0% had university or higher education, while 6.3% of men and 3.0% of women had lower than secondary education. Over one-third of the participants (36.3%) were from Yerevan and 63.7% from the provinces of Armenia. Further descriptive findings and comparisons between the groups with and without HBV markers are provided in Tables 1 and 2 and discussed later in the Results.

Prevalence of past and present HBV infection

For peer review only - http://bmjopen.bmj.com/site/about/guidelines.xhtml

The weighted percentage of those positive for anti-HBc antibodies indicating past or present HBV infection was 14.1% (95% CI 13.1%-15.2%). This percentage significantly increased with age (Figure 1) and was slightly higher among men than women (15.0% versus 12.2%, p=0.017). The weighted percentage of those carrying HBsAg – the prevalence of chronic HBV infection – was 0.8% (95% CI 0.5%-1.1%). This estimate was the highest in the age group of 30-39 years old (1.5%, 95% CI 0.8%-2.6%) (Figure 1) and was higher among men compared to women (1.1% versus 0.5%, p=0.048). The measurement of the viral load among those positive for HBsAg showed that for 61.9% of them, HBV DNA was below 150 IU/ml, for 30.2% it was between 150 IU/ml and 2,000 IU/ml, and for 7.9% (n=2) – over 2,000 IU/ml (in the weighted analysis). For both these cases, the viral load was 11,000 IU/ml. For those HBV DNA-positive individuals with measurable (≥150 IU/ml) viral load, the mean level of HBV DNA was 2787.6 copies/ml (SD 4441.0) and the interquartile range – 750 copies/ml. None of the HBsAg-positive samples were positive for HDV RNA.

Results of descriptive comparisons

Table 1 demonstrates the results of weighted crude comparisons of the study variables between the groups infected with HBV ever in life and those never infected with HBV.

Table 1 Distribution of selected variables by participants' anti-HBV core antibody status, Armenia, 2021

			Anti-HBV core antibody			
		N*	Positive (n=469)	Negative (n=3005)	*p-value*	Total (n=3474)
Socioeconomic variables:						
Age, mean (SD)		3473	57.1 (15.7)	44.0 (16.9)	*<0.001*	45.8 (17.3)
Socio-economic status score, mean (SD)		2696	46.8 (34.8)	50.3 (35.3)	*0.075*	49.9 (35.2)
Family size, mean (SD)		3451	4.2 (2.1)	4.4 (1.7)	*0.004*	4.4 (1.8)
Sex:	Female, %	3473	49.4	55.3	*0.017*	54.5
	Male, %		50.6	44.7		45.5
Education:	Below secondary, %	3456	7.7	4.0	*<0.001*	4.5
	Secondary/vocational, %		61.5	52.0		53.3
	University/higher, %		30.8	44.0		42.3

For peer review only - http://bmjopen.bmj.com/site/about/guidelines.xhtml

			Anti-HBV core antibody			
		N*	Positive (n=469)	Negative (n=3005)	*p-value*	Total (n=3474)
Employment:	Employed/student, %	3438	41.8	58.6	*<0.001*	56.4
	Unemployed/seasonal migrant, %		27.2	28.9		28.7
	Retired/disabled, %		30.9	12.5		15.0
Residence:	Yerevan city, %		28.6	37.4	*<0.001*	36.3
	Provinces, %		71.4	62.6		63.7
Health status variables:						
Anti-Hepatitis C antibodies positive, %		3474	3.6	1.7	*0.011*	2.0
Hepatitis C PCR test positive, %		3474	1.5	0.6	*0.034*	0.7
Diabetes, %		3395	12.4	7.1	*<0.001*	7.8
Chronic liver disease, %		3391	8.4	2.1	*<0.001*	2.9
Obesity, %		3383	13.8	9.5	*0.007*	10.1
Heart disease, %		3390	29.0	12.6	*<0.001*	14.8
Cancer, %		3394	2.0	1.5	*0.408*	1.5
Chronic kidney disease, %		3395	4.6	3.6	*0.286*	3.7
Asthma requiring medication, %		3391	2.0	2.3	*0.864*	2.2
Symptoms experienced during the last six months:						
Vomiting, %		3387	3.3	4.6	*0.268*	4.5
Fatigue, %		3391	41.8	46.0	*0.104*	45.4
Loss of appetite, %		3388	8.9	14.4	*0.001*	13.6
Joint ache, %		3393	37.6	33.4	*0.078*	33.9
Abdominal pain, %		3382	9.4	11.2	*0.257*	11.0
Fever, %		3395	16.5	25.4	*<0.001*	24.2
Nose bleed, %		3387	2.9	4.6	*0.108*	4.4
Health literacy variables:						
Poor knowledge on infectious diseases, %		3474	32.8	24.3	*<0.001*	25.5
Health information understanding score, mean (SD)		3005	52.2 (11.5)	54.4 (9.9)	*<0.001*	54.1 (10.1)
Health behavioral variables:						
Current smoking, %		3389	25.7	20.7	*0.016*	21.4
Ever smoking, %		3395	39.5	34.0	*0.026*	34.7
Having tattoos, %		3380	13.0	10.0	*0.055*	10.4
Undergoing blood transfusion, %		3380	4.0	3.4	*0.487*	3.5
Ever having been imprisoned, %		3387	4.9	1.2	*<0.001*	1.7
Visiting a dentist:	Once in 3-5 years/more, %	3329	52.5	74.3	*<0.001*	71.4
	Less frequently/never, %		47.5	25.7		28.6
Vaccinated against Hepatitis B (self-reported), %		3397	0.2	0.2	*1.000*	0.2
Hepatitis B told by doctor, %		3397	2.7	0.2	*<0.001*	0.5

N, Number of valid responses; HBV, Hepatitis B virus; PCR, Polymerase chain reaction; SD, Standard deviation.
* Weighted estimates; *p-value:* two-sided.

The ever-infected group was older, with lower level of education, from a smaller-size family, and more often – residing in provinces. The proportion of employed was lower among ever infected group and the proportion of retired/disabled was higher. Anti-hepatitis C antibodies

For peer review only

were found among the ever-infected group more frequently. The ever-infected group more frequently reported several chronic health conditions – diabetes, chronic liver disease, obesity, and heart disease. However, some symptoms experienced during the last six months, including loss of appetite and abdominal pain, were less frequently reported by the ever-infected group. The ever-infected group demonstrated poor knowledge on infectious diseases. The mean score of understanding health related information was also lower among them compared to the never infected group. There were significant differences between the groups in the health behavioral domain as well. Both current and ever smokers were more prevalent among the ever infected. Higher proportion of them were ever imprisoned, and lower proportion used dental services at least once in 3-5 years. A similar negligible proportion of respondents (0.2%) in both groups reported having been vaccinated against hepatitis B.

The characteristics of those participants with and without chronic HBV infection were compared in Table 2.

Table 2 Distribution of selected variables by Hepatitis B surface antigen (HBsAg) status, Armenia, 2021

			HBsAg			
		N*	Positive (n=27)	Negative (n=3447)	*p-value*	Total (n=3474)
Socioeconomic variables:						
Age, mean (SD)		3473	44.7 (11.9)	45.8 (17.4)	*0.734*	45.8 (17.3)
Socio-economic status score, mean (SD)		2696	43.7 (24.3)	49.9 (35.3)	*0.471*	49.9 (35.2)
Family size, mean (SD)		3451	4.2 (1.3)	3.4 (1.8)	*0.595*	4.4 (1.8)
Sex:	Female, %	3473	40.7	54.7	*0.048*	54.5
	Male, %		65.4	45.3		45.5
Education:	Below secondary, %	3455	22.2	4.3	*<0.001*	4.5
	Secondary/vocational, %		44.4	53.3		53.3
	University/higher, %		33.3	42.3		42.3
Employment:	Employed/student, %	3439	48.1	56.4	*0.607*	56.4
	Unemployed/seasonal migrant, %		37.0	28.6		28.7
	Retired/disabled, %		14.8	15.0		15.0
Residence:	Yerevan city, %		29.6	36.3	*0.551*	36.3
	Provinces, %		70.4	63.7		63.7
Health status variables:						
Anti-Hepatitis C antibodies positive, %		3474	7.4	1.9	*0.097*	2.0
Hepatitis C PCR test positive, %		3474	3.7	0.7	*0.171*	0.7
Diabetes, %		3396	11.1	7.8	*0.464*	7.8
Chronic liver disease, %		3390	19.2	2.8	*0.001*	2.9

For peer review only - http://bmjopen.bmj.com/site/about/guidelines.xhtml

	N*	HBsAg Positive (n=27)	HBsAg Negative (n=3447)	*p-value*	Total (n=3474)
Obesity, %	3384	14.8	10.0	*0.343*	10.1
Heart disease, %	3389	14.8	14.8	*1.000*	14.8
Cancer, %	3395	0.0	1.5	*1.000*	1.5
Chronic kidney disease, %	3395	3.7	3.7	*1.000*	3.7
Asthma requiring medication, %	3391	3.7	2.2	*0.459*	2.2
Symptoms experienced during the last six months:					
Vomiting, %	3388	0.0	4.5	*0.632*	4.5
Fatigue, %	3392	40.7	45.5	*0.700*	45.4
Loss of appetite, %	3388	0.0	13.7	*0.042*	13.6
Joint ache, %	3394	48.1	33.8	*0.151*	33.9
Abdominal pain, %	3382	4.2	11.0	*0.508*	11.0
Fever, %	3395	11.1	24.3	*0.173*	24.2
Nose bleed, %	3387	0.0	4.4	*0.630*	4.4
Health literacy variables:					
Poor knowledge on infectious diseases, %	3474	37.0	25.4	*0.183*	25.5
Health information understanding score, mean (SD)	3005	48.7 (14.4)	54.2 (10.1)	*0.016*	54.1 (10.1)
Health behavioral variables:					
Current smoking, %	3390	44.4	21.2	*0.007*	21.4
Ever smoking, %	3394	53.8	34.6	*0.060*	34.7
Having tattoos, %	3379	19.2	10.3	*0.182*	10.4
Undergoing blood transfusion, %	3380	3.7	3.5	*0.615*	3.5
Ever having been imprisoned, %	3387	11.6	1.6	*0.010*	1.7
Visiting a dentist: Once in 3-5 years/more, %	3330	74.1	71.4	*0.834*	71.4
Less frequently/never, %		25.9	28.6		28.6
Vaccinated against Hepatitis B (self-reported), %	3398	0.0	0.2	*1.000*	0.2
Hepatitis B told by doctor, %	3398	22.2	0.4	*<0.001*	0.5

N, Number of valid responses; PCR, Polymerase chain reaction; SD, Standard deviation.
* Weighted estimates; *p-value:* two-sided.

Those chronically infected had lower education and higher composition of male. They reported chronic liver disease much more frequently. The proportion of those demonstrating poor knowledge on infectious diseases was higher among them and the mean score of understanding health-related information was lower. Smoking was more frequent among them, and they have been imprisoned much more frequently than those free from the virus.

Among those testing positive for anti-HBc, 12 (2.7%) reported that they have ever been told by a doctor that they were infected with hepatitis B. Six of these 12 persons were also positive for HBsAg, thus only 22.2% of those HBsAg positive were aware of their status (Tables 1, 2).

For peer review only - http://bmjopen.bmj.com/site/about/guidelines.xhtml

For peer review only

Findings of regression analysis

The fitted logistic regression model of factors independently associated with being ever infected with HBV included the following characteristics – age, education, Yerevan vs. province residence, chronic liver disease, current smoking, imprisonment ever in life, and knowledge on infectious diseases (Table 3).

Table 3 Multivariable logistic regression model of determinants of HBV infection ever in life (positivity for anti-HBV core antibody) among adult population in Armenia, 2021 (valid N=3349)

Characteristics		aOR	95% CI	*p- value*
Age, years		1.04	1.04 - 1.05	< 0.001
Education:	Less than secondary	1.61	1.02 - 2.56	0.042
	Secondary/vocational	1.26	1.00 - 1.59	0.052
	University/higher	1.00	ref.	ref.
Living in Yerevan vs. provinces		0.74	0.59 - 0.93	0.011
Chronic liver disease		2.93	1.88 - 4.56	<0.001
Current smoking		1.60	1.25 - 2.06	<0.001
Ever imprisoned		2.53	1.41 - 4.56	0.002
Poor knowledge on infectious diseases		1.32	1.05 - 1.67	0.018

HBV, hepatitis B virus.
N, number of cases.
aOR, adjusted odds ratio.
CI, confidence interval.

When holding all the other variables constant, each year increase in age increased the likelihood of ever being infected with HBV by 4%. Compared to having university/higher education, having less than secondary education was associated with 1.61 times higher odds, and having secondary/vocational education with 1.26 times higher odds of ever being infected with HBV. Residing in Yerevan was protective – 0.74 times decreasing the odds of HBV infection ever in life. Suffering from chronic liver disease was associated with 2.93 times higher odds of HBV infection ever in life. Ever being imprisoned increased the odds of HBV infection ever in life 2.53 times. Current smoking was also a risk factor for ever having HBV infection, increasing its odds 1.60 times. Poor knowledge of infectious diseases was associated with 1.32 times higher

For peer review only - http://bmjopen.bmj.com/site/about/guidelines.xhtml

odds of having HBV infection ever in life. The model had no collinearity issues with all Variance Inflation Factor (VIF) values being below 1.13.

Several factors associated with having HBV infection ever in life, including education, chronic liver disease, and current smoking, were also significant in the fitted model of determinants of chronic HBV infection (Table 4).

Table 4 Multivariable logistic regression model of determinants of chronic HBV infection (HBsAg positivity) among adult population of Armenia, 2021 (valid N=3349)

Characteristics		aOR	95% CI	*p- value*
Education:	Less than secondary	6.44	2.17 - 19.12	<0.001
	Secondary/vocational	1.01	0.42 - 2.45	0.984
	University/higher	1.00	ref.	ref.
Chronic liver disease		8.44	3.00 - 23.72	<0.001
Current smoking		2.56	1.16 - 5.65	0.019
Ever imprisoned		3.33	0.81 - 13.74	0.096

HBV, hepatitis B virus.
HBsAg, Hepatitis B surface antigen.
N, number of cases.
aOR, adjusted odds ratio.
CI, confidence interval.

For peer review only

Compared to secondary or higher education, less than secondary education increased the odds of having chronic HBV infection 6.4 times. Chronic liver disease was associated with 8.4 times higher odds of having chronic HBV infection. Current smoking increased the likelihood of chronic HBV infection 2.6 times. Ever having been imprisoned was also potentially associated with having chronic HBV infection, as the observed effect range between these variables was suggestive of a weak negative to a strong positive association (aOR 3.33; 95% CI 0.81 - 13.74). Again, no collinearity issues were identified in this model with all VIF values being below 1.06.

DISCUSSION

This is the first population-based seroprevalence study to estimate the nationwide prevalence of HBV infection in Armenia. In our study, which included 3,831 participants over 18 years of age

For peer review only - http://bmjopen.bmj.com/site/about/guidelines.xhtml

from Armenia, we found that the weighted prevalence of chronic HBV infection was 0.8% (95% CI 0.5%-1.1%), and 14.1% (95% CI 13.1%-15.2%) had been ever infected with hepatitis B. Odds for ever being infected with hepatitis B increased by age. None of the HBsAg-positive participants were positive for HDV RNA.

While the proportion of adult population ever infected with hepatitis B was considerably high in Armenia, the prevalence of chronic infection was low. In comparison to most up-to-date population-based seroprevalence survey results from countries with intermediate hepatitis B endemicity in European Region before introduction of universal hepatitis B vaccination, the prevalence was lower than in a study carried out in Georgia in 2021: the prevalence of chronic hepatitis B among adults in Georgia was 2.7% (95% CI 2.3%-3.4%) and the proportion of persons ever infected with hepatitis B was 21.7% (95% CI 20.4%-23.2%).(20).

Previous data for Armenia based on modeling studies show a higher prevalence of chronic infection. According to a modeling study from 2016, the HBsAg prevalence in Armenia was estimated at 1.9%,(21) and based on the Coalition for Global Hepatitis Elimination estimates, it was gradually decreasing from 3.4% in 1994/95.(22) Surveillance data confirm that there has been a continuous reduction of new cases mainly explained by introduction of hepatitis B vaccination among children in 1999.(13, 15) This will likely contribute to further decrease in prevalence of chronic HBV infection in Armenia.(23) The prevalence in 2016 is likely an overestimation as it was relying on proxy data and not a measurement attributable to general population in Armenia. Data from screening of pregnant women in Armenia support the current finding of low prevalence. For the years 2017 – 2021, from over 97,000 pregnant women that underwent screening, 0.66% were HBsAg positive.(24)

In crude weighted comparisons, the prevalence of both HBsAg and anti-HBc was higher in males than in females, which is similar to other studies.(20, 25, 26) Generally, difference in the prevalence between men and women may be related to either behavioral factors like higher

For peer review only - http://bmjopen.bmj.com/site/about/guidelines.xhtml

sexual risk behaviors, intravenous drug use and imprisonment in men, or to biological factors.(27) Based on studies, it has been discussed that females usually develop more intense innate, humoral and cellular responses to viral infections, including hepatitis virus, than males.(27, 28) After controlling for other variables, this association, however, disappeared in the model.

While for ever being infected with HBV, it was possible to detect a statistically significant increase in prevalence with increasing age, there were no detectable differences with increasing age in the prevalence of chronic hepatitis B. However, the analysis of HBsAg prevalence by different groups was limited by low number of positive cases and, correspondingly, lack of power with the given sample size. Descriptive results, however, show that there were no chronic hepatitis B cases among those 18-29 years of age. Individuals up to age of 22 have been born after the introduction of birth dose and childhood hepatitis B vaccination in Armenia. This is potentially leading to less cases in this age group. According to the reports submitted to WHO, the impact of hepatitis B vaccination on control of mother to child transmission has been demonstrated in several countries of the WHO European Region with historically intermediate and high hepatitis B endemicity, like Republic of Moldova, Georgia, Kyrgyzstan, Uzbekistan, Tajikistan and Turkmenistan.(23, 29) We also did not find any HBsAg positive cases among those 70 years and older, while the percentage positive for anti-HBc was highest in this group. On one hand, those with persistent chronic infection might have already had a lethal outcome at that age, on other hand, the sample size in this age group (n=274) might have been a limiting factor to detect prevalent cases. Furthermore, the HBsAg clearance rate with and without therapy might also influence this figure.(30) Several other factors associated with higher odds for current or past infection were identified in the multivariable regression models. For both being ever infected and chronic infection, odds were higher among people with less than secondary education and current smokers. Odds for being ever infected were higher among persons living

For peer review only - http://bmjopen.bmj.com/site/about/guidelines.xhtml

in provinces (versus Yerevan), those demonstrating poorer knowledge on infectious diseases, and those ever imprisoned. Individuals with lower levels of education, smoking, and individuals who have been imprisoned might be more likely to engage in riskier behavior such as drug use and unprotected sexual activity leading to increased exposure to HBV.(31-33) Furthermore, lower education levels and socioeconomic disadvantages can result in reduced access to healthcare services and prevention.(34, 35) Higher odds for ever being infected with HBV in provinces compared to Yerevan could be as well related to historically poorer access to health care or lower level of infection prevention and control measures in regional heath care centers. Though, there is no documented evidence supporting this assumption. We could also demonstrate that poorer knowledge about infectious diseases was associated with higher odds for detection of anti-HBc, possibly explained by poorer knowledge about transmission routes, limited understanding of preventive measures, misconceptions and stigma and poorer access to health related information.(33, 36, 37) Findings on associated factors suggest that information campaigns and prevention, especially among groups having higher risk for infection due to behavioral or socio-economic reasons, are needed to further decrease hepatitis B incidence.

In 7.9% of chronic infections (n=2 in the weighted analysis), the viral load was above 10,000 IU/ml, indicating the need for treatment.(38) A viral load above 10,000 IU/ml is a strong risk predictor of HCC, independent of HBeAg status, ALT level and liver cirrhosis.(39-43) The results on viral load suggest that there is a tangible proportion of chronic hepatitis B patients in Armenia that are currently not receiving antiviral treatment or were not aware of their infection. This is supported by the fact that only 22% of those testing positive for HBsAg reported that they have ever been told by a doctor that they were infected with HBV, thus, were not diagnosed previously. As the testing and treatment costs in case of a chronic HBV infection are currently mainly related to out-of-pocket payments in Armenia, the detection of cases and sustaining them in care is hindered.(24). Improvement of state-funded preventive and testing strategies and

For peer review only - http://bmjopen.bmj.com/site/about/guidelines.xhtml

warranting access to state-funded hepatitis B treatment is needed in order to find and timely treat cases to prevent sequel like cirrhosis and HCC, and decrease mortality.

Based on our prevalence findings (0.8%) and given the adult population size of Armenia (~2,500,000), the estimated number of people living with chronic HBV infection in Armenia could be around 20,000, and 1,600 of them (7.9% with a viral load above 10,000 IU/ml) could be at high risk of HCC and need immediate treatment. Yet, roughly, only one-fifth of chronically infected are aware of their infection. The WHO target for 2030 is 90% of people living with hepatitis B diagnosed – calling for improved access to testing to reach the global and regional goals.(44) Hence, there is a need to revise testing and treatment strategies in Armenia in order to find and timely treat chronic hepatitis B cases to prevent sequel and reduce mortality.

Limitations: Due to limited length of the questionnaire we were not able to include all relevant questions to determine other factors associated with higher odds of being infected with HBV. Furthermore, as the sample size was calculated for the Sars-CoV-2 study needs, our possibilities for calculation of prevalence of HBV infection for different groups were limited and we might lack power to detect differences among groups and when performing regression analyses. At the same time, the sample size was appropriate to measure nationwide robust estimates with narrow confidence intervals.

CONCLUSIONS

This is the first nation-wide representative study in Armenia providing a robust estimate of anti-HBc and HBsAg prevalence among general population. While the prevalence of chronic hepatitis B is rather low in Armenia, the proportion of persons ever been infected with HBV is considerable. Age distribution of HBV infection ever in life suggests that there has been historically high incidence and that new infections possibly still occur among adults. Meanwhile,

For peer review only - http://bmjopen.bmj.com/site/about/guidelines.xhtml

the undetectable prevalence among younger age groups suggests the positive impact of the hepatitis B vaccination.

RECOMMENDATIONS

In order to find and treat persons living with HBV, testing should be scaled up in the first line considering population groups that are likely more affected. Furthermore, to reach elimination of hepatitis B, prevention of mother-to-child transmission should be maintained at high level.(45) Validation of reaching hepatitis B control targets with a study targeting younger children (from the vaccinated cohort) could be considered in Armenia.

Furthermore, prevention among population groups at high risk for HBV infection trough vaccination and access to needle-syringe programs should be in place and at sufficient coverage to further decrease HBV prevalence in Armenia. Actions to improve knowledge on hepatitis B transmission routes, prevention and testing are needed for all population groups.

Acknowledgements This work was done with partial support from WHO Regional Office for Europe and technical assistance from Robert Koch Institute. The authors alone are responsible for the views expressed in this article and they do not necessarily represent the views, decisions, or policies of the institutions with which they are affiliated.

Contributors AD, SS, LM, AM, NS, GG and VP conceptualized this study, AD performed the analysis. AD, SD, LA, and DM drafted the manuscript. All authors supported the interpretation of the study results and critically reviewed and revised the manuscript.

Competing interests None declared.

For peer review only

For peer review only - http://bmjopen.bmj.com/site/about/guidelines.xhtml

Funding The data collection was partially funded by WHO Regional Office for Europe (no grant number). No funding was received for developing this manuscript.

Data availability statement Data are available on reasonable request.

Ethics Approval Statement The study protocol was reviewed and approved by the Ethics Review Committee of the World Health Organization (protocol ID: CERC.0013C) and the Institutional Review Board of the American University of Armenia (protocol ID: AUA-2021-005), as conforming to the principles embodied in the Declaration of Helsinki.

For peer review only

REFERENCES

1. G. B. D. Hepatitis B Collaborators. Global, regional, and national burden of hepatitis B, 1990-2019: a systematic analysis for the Global Burden of Disease Study 2019. Lancet Gastroenterol Hepatol. 2022;7(9):796-829. doi: 10.1016/S2468-1253(22)00124-8

2. Purcell RH. The discovery of the hepatitis viruses. Gastroenterology. 1993;104(4):955-63. doi: 10.1016/0016-5085(93)90261-a

3. Tseng TC, Liu CJ, Yang HC, et al. High levels of hepatitis B surface antigen increase risk of hepatocellular carcinoma in patients with low HBV load. Gastroenterology. 2012;142(5):1140-9 e3; quiz e13-4. doi: 10.1053/j.gastro.2012.02.007

4. Zhao X, Shi X, Lv M, et al. Prevalence and factors associated with hepatitis B virus infection among household members: a cross-sectional study in Beijing. Hum Vaccin Immunother. 2021;17(6):1818-24. doi: 10.1080/21645515.2020.1847951

5. Karoney MJ, Some FF, Dip HIL, et al. Burden of Hepatitis B Infection among High Risk Populations in Western Kenya. J Infect Dis Epidemiol 2020;6:132. doi: 10.23937/2474-3658/1510132

6. Muljono DH, Wijayadi T, Sjahril R. Hepatitis B Virus Infection among Health Care Workers in Indonesia. Euroasian J Hepatogastroenterol. 2018;8(1):88-92. doi: 10.5005/jp-journals-10018-1269

7. Hou J, Liu Z, Gu F. Epidemiology and Prevention of Hepatitis B Virus Infection. Int J Med Sci. 2005;2(1):50-7. doi: 10.7150/ijms.2.50

8. Gnyawali B, Pusateri A, Nickerson A, et al. Epidemiologic and socioeconomic factors impacting hepatitis B virus and related hepatocellular carcinoma. World J Gastroenterol. 2022;28(29):3793-802. doi: 10.3748/wjg.v28.i29.37934.

For peer review only - http://bmjopen.bmj.com/site/about/guidelines.xhtml

9. Tosun S, Aygun O, Ozdemir HO, et al. The impact of economic and social factors on the prevalence of hepatitis B in Turkey. BMC Public Health. 2018;18(1):649. doi: 10.1186/s12889-018-5575-6

10. Pattyn J, Hendrickx G, Vorsters A, et al. Hepatitis B Vaccines. J Infect Dis. 2021;224(12 Suppl 2):S343-S51. doi: 10.1093/infdis/jiaa668

11. Ott JJ, Stevens GA, Groeger J, et al. Global epidemiology of hepatitis B virus infection: new estimates of age-specific HBsAg seroprevalence and endemicity. Vaccine. 2012;30(12):2212-9. doi: 10.1016/j.vaccine.2011.12.116

12. Andreasyan D, Bazarchyan A. "Health and Health Care" Yearbook. Yerevan: National Institute of Health Named after Academician S. Avdalbekyan; 2022. 299 p.

13. Demirchyan A, Mirzoyan L, Thompson ME. Synthesis of the existing data on Hepatitis B in Armenia. Yerevan: American University of Armenia, Center for Health Services Research; 2000. https://aua.am/chsr/PDF/2000/HepatitB_FINAL.pdf

14. Ghazinyan H, Mkhitaryan A, Melik-Andreasyan G, et al. Updating HBV status in Armenia. Optimal Management of Hepatitis B Virus Infection; 25–27 September Athens, Greece: Proceedings of EASL Special Conference; 2014. p. 159.

15. Melik-Andreasyan GG, Markosyan IE, Voskanyan LS, et al. Epidemiological Characteristics of Viral Hepatitis in Armenia (in Russian). В мире вирусных гепатитов. 2013;4:20-6.

16. Davlidova S, Haley-Johnson Z, Nyhan K, et al. Prevalence of HIV, HCV and HBV in Central Asia and the Caucasus: A systematic review. Int J Infect Dis. 2021;104:510-25. doi: 10.1016/j.ijid.2020.12.068

17. Roche Diagnostics. Elecsys® Anti-HBc II. Immunoassay for the qualitative determination of total antibodies against hepatitis B core antigen (HBcAg) [Available from: https://diagnostics.roche.com/global/en/products/params/elecsys-anti-hbc-ii.html].

For peer review only - http://bmjopen.bmj.com/site/about/guidelines.xhtml

18. Roche Diagnostics. Elecsys® HBsAg II. Immunoassay for the qualitative determination of hepatitis B surface antigen (HBsAg) [Available from: https://diagnostics.roche.com/global/en/products/params/elecsys-hbsag-ii.html].

19. Hosmer DW, Lemeshow S. Applied logistic regression. 2nd ed. New York: A Wiley-Interscience Publication; 2000: 92-116.

20. Khetsuriani N, Gamkrelidze A, Shadaker S, et al. Hepatitis B prevalence and the impact of vaccination in Georgia: results from a nationwide serosurvey International Liver Congress TM 2022, London22-26 June 2022 [Available from: https://www.postersessiononline.eu/173580348_eu/congresos/ILC2022/aula/-THU_283_ILC2022.pdf].

21. Polaris Observatory C. Global prevalence, treatment, and prevention of hepatitis B virus infection in 2016: a modelling study. Lancet Gastroenterol Hepatol. 2018;3(6):383-403. doi: 10.1016/S2468-1253(18)30056-6

22. Coalition for Global Hepatitis Elimination. Data Dashboard Armenia. [Available from: https://www.globalhep.org/country-progress/armenia] (accessed 27 July 2023).

23. Khetsuriani N, Tishkova F, Jabirov S, et al. Substantial decline in hepatitis B virus infections following vaccine introduction in Tajikistan. Vaccine. 2015;33(32):4019-24. doi: 10.1016/j.vaccine.2015.05.092

24. Robert Koch Institute. Report: Technical Assessment of Viral Hepatitis Response in Armenia. Berlin; 2023: 16-19.

25. Khan NU, Zalan A, Petruzziello A, et al. Determining the Actual Prevalence of Hepatitis B in Khyber Pakhtunkhwa-Pakistan: A Meta-Analysis. Open Virol J. 2018;12:33-41. doi: 10.2174/1874357901812010033

For peer review only - http://bmjopen.bmj.com/site/about/guidelines.xhtml

26. Wang H, Men P, Xiao Y, et al. Hepatitis B infection in the general population of China: a systematic review and meta-analysis. BMC Infect Dis. 2019;19(1):811. doi: 10.1186/s12879-019-4428-y

27. Brown R, Goulder P, Matthews PC. Sexual Dimorphism in Chronic Hepatitis B Virus (HBV) Infection: Evidence to Inform Elimination Efforts. Wellcome Open Res. 2022;7:32. doi: 10.12688/wellcomeopenres.17601.2

28. Ruggieri A, Gagliardi MC, Anticoli S. Sex-Dependent Outcome of Hepatitis B and C Viruses Infections: Synergy of Sex Hormones and Immune Responses? Front Immunol. 2018;9:2302. doi: 10.3389/fimmu.2018.02302

29. More countries reaching hepatitis B control targets brings the WHO European Region closer to eliminating viral hepatitis as a public health threat [press release]. https://www.who.int/europe/news/item/24-04-2023-more-countries-reaching-hepatitis-b-control-targets-brings-the-who-european-region-closer-to-eliminating-viral-hepatitis-as-a-public-health-threat2023 (accessed 25 April 2023).

30. Terrault NA, Wahed AS, Feld JJ, et al. Incidence and prediction of HBsAg seroclearance in a prospective multi-ethnic HBeAg-negative chronic hepatitis B cohort. Hepatology. 2022;75(3):709-23. doi: 10.1002/hep.32231

31. Galea S, Vlahov D. Social determinants and the health of drug users: socioeconomic status, homelessness, and incarceration. Public Health Rep. 2002;117 Suppl 1(Suppl 1):S135-45.

32. MacLachlan JH, Cowie BC. Hepatitis B virus epidemiology. Cold Spring Harb Perspect Med. 2015;5(5):a021410. doi: 10.1101/cshperspect.a021410

33. Corona R, Caprilli F, Giglio A, et al. Risk factors for hepatitis B virus infection among heterosexuals attending a sexually transmitted diseases clinic in Italy: role of genital ulcerative diseases. J Med Virol. 1996;48(3):262-6. doi: 10.1002/(SICI)1096-9071(199603)48:3<262::AID-JMV8>3.0.CO;2-6

For peer review only

For peer review only - http://bmjopen.bmj.com/site/about/guidelines.xhtml

34. McMaughan DJ, Oloruntoba O, Smith ML. Socioeconomic Status and Access to Healthcare: Interrelated Drivers for Healthy Aging. Front Public Health. 2020;8:231. doi: 10.3389/fpubh.2020.00231

35. Brodzinski A, Neumeyer-Gromen A, Dudareva S, et al. [Hepatitis B virus infection and vaccine-induced immunity: the role of sociodemographic determinants: Results of the study "German Health Interview and Examination Survey for Adults" (DEGS1, 2008-2011)]. Bundesgesundheitsblatt Gesundheitsforschung Gesundheitsschutz. 2022;65(2):159-69. doi: 10.1007/s00103-021-03473-z

36. Machmud PB, Glasauer S, Gottschick C, et al. Knowledge, Vaccination Status, and Reasons for Avoiding Vaccinations against Hepatitis B in Developing Countries: A Systematic Review. Vaccines (Basel). 2021;9(6). doi: 10.3390/vaccines9060625

37. Pereira LM, Martelli CM, Merchan-Hamann E, et al. Population-based multicentric survey of hepatitis B infection and risk factor differences among three regions in Brazil. Am J Trop Med Hyg. 2009;81(2):240-7.

38. European Association for the Study of the Liver. EASL 2017 Clinical Practice Guidelines on the management of hepatitis B virus infection. J Hepatol. 2017;67(2):370-98. doi: 10.1016/j.jhep.2017.03.021

39. Chen CJ, Yang HI, Su J, et al. Risk of hepatocellular carcinoma across a biological gradient of serum hepatitis B virus DNA level. JAMA. 2006;295(1):65-73. doi: 10.1001/jama.295.1.65

40. Liu CJ, Chen BF, Chen PJ, et al. Role of hepatitis B viral load and basal core promoter mutation in hepatocellular carcinoma in hepatitis B carriers. J Infect Dis. 2006;193(9):1258-65. doi: 10.1086/502978

41. Barcena Marugan R, Garcia Garzon S. DNA-guided hepatitis B treatment, viral load is essential, but not sufficient. World J Gastroenterol. 2009;15(4):423-30. doi: 10.3748/wjg.15.423

For peer review only - http://bmjopen.bmj.com/site/about/guidelines.xhtml

42. Mendy ME, Welzel T, Lesi OA, et al. Hepatitis B viral load and risk for liver cirrhosis and hepatocellular carcinoma in The Gambia, West Africa. J Viral Hepat. 2010;17(2):115-22. doi: 10.1111/j.1365-2893.2009.01168.x

43. Kim GA, Han S, Choi GH, et al. Moderate levels of serum hepatitis B virus DNA are associated with the highest risk of hepatocellular carcinoma in chronic hepatitis B patients. Aliment Pharmacol Ther. 2020;51(11):1169-79. doi: 10.1111/apt.15725

44. World Health Organization. Global Hepatitis Report. Geneva: Licence: CC BY-NC-SA 3.0 IGO.; 2017. https://apps.who.int/iris/bitstream/handle/10665/255016/9789241565455-eng.pdf?sequence=1&isAllowed=y

45. Polaris Observatory, C., Global prevalence, cascade of care, and prophylaxis coverage of hepatitis B in 2022: a modelling study. Lancet Gastroenterol Hepatol, 2023. Doi: 10.1016/S2468-1253(23)00197-8

Figure Legend:

Figure 1 Weighted prevalence (%) estimates of HBV core antibody and HBV surface antigen by 10-year age groups among the study participants, Armenia, 2021.

For peer review only

For peer review only - http://bmjopen.bmj.com/site/about/guidelines.xhtml

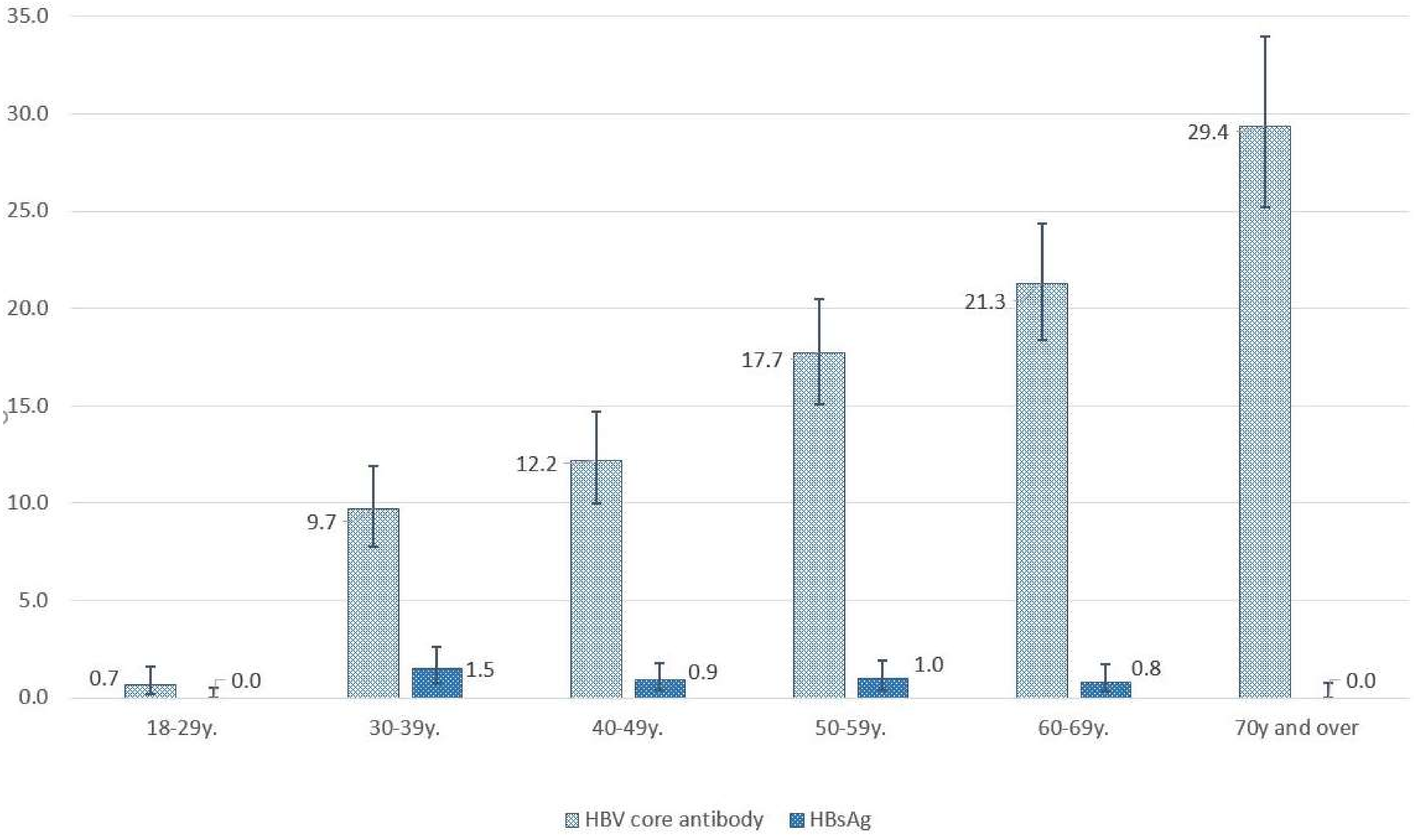
35.0
30.0
25.0
20.0
15.0
10.0
5.0
0.0
0.7
0.0
9.7
1.5
12.2
0.9
17.7
1.0
21.3
0.8
29.4
0.0
18-29y.
30-39y.
40-49y.
50-59y.
60-69y.
70y and over
HBV core antibody
HBsAg

Knowledge, Attitude, and Practices Questionnaire on Infectious Diseases, Chronic Hepatitis B and C Virus Infections and Associated Risk Factors

Interviewer

_ _ _ _ _ _ _ _

Participant ID

_ _/_ _/_ _ _/

Blood sampling for Hepatitis

Do not read out loud. Mark as it is mentioned in Excel file.

() Yes → *Show Section 17*

() No → *Skip Section 17*

Date

dd/mm/yy

______ / ______ / ___________

Start of the interview

___:___ (hour:min)

1. Socio-demographic characteristics

The following questions are about your demographic characteristics

1.1 Your nationality

( ) Armenian

( ) Russian

( ) Yazidi

( ) Other, please specify ______________________________

( ) [prefer not to answer]

1.2 Place of residence

( ) City

( ) Village

( ) [prefer not to answer]

For peer review only - http://bmjopen.bmj.com/site/about/guidelines.xhtml

1.3 Your gender

Do not read out loud

( ) Male

( ) Female

1.4 How old are you in the number of years completed? (How old were you on your last birthday)?

dd/mm/yy

_____ / _______ / ____

( ) [prefer not to answer]

1.5 Your highest level of education

Read out all of the options

( ) Incomplete secondary (less than 10 years)
( ) Secondary (10-12 years: high school)
( ) Vocational (12-13 years)
( ) University
( ) Post-graduate
( ) [prefer not to answer]

1.6 How many members are in your family, including yourself?

________________ members

( ) [prefer not to answer]

1.7 Are you currently employed?

Read out all of the options

() Yes, please specify what is your occupation?_________________

() No, I am not employed
() No, I am retired
() No, I am a student
() I work from home/ have my own business
() I am a seasonal worker in foreign countries
() I am a farmer
() Other (specify) ______________________

( ) [prefer not to answer]

For peer review only - http://bmjopen.bmj.com/site/about/guidelines.xhtml

For peer review only

1.8 On average, how much money does your family spend monthly? (Choose one option)

( ) Less than 100,000 AMD
( ) From 101,000 to 200,000 AMD
( ) From 201,000 to 300,000 AMD
( ) From 301,000 to 400,000 AMD
( ) Above 401,000 AMD
( ) Don't know/ Refusal

2. The following questions refer to health and infectious disease literacy.

2.1 How often do you need to have someone help you when you read instructions, pamphlets, or other written material from your doctor or pharmacy? (R*ead the response options, except [prefer not to answer] option)*

1. Never
2. Rarely
3. Sometimes
4. Often
5. Always
6. [prefer not to answer]

On a scale from 1 to 7, how easy or difficult would you say it is to…:

Note to interviewers: Repeat the full question as needed

2.2…find the information you need related to infectious diseases (for example, hepatitis, measles, influenza, COVID-19, HIV, etc.)?

Very difficult [1] [2] [3] [4] [5] [6] [7] Very easy

( ) [prefer not to answer]

2.3…understand information about what to do if you think you have an infectious disease (for example, measles, influenza, COVID-19, HIV, etc.)?

Very difficult [1] [2] [3] [4] [5] [6] [7] Very easy

( ) [prefer not to answer]

2.4…judge if the information about infectious diseases in the media is reliable?

Very difficult [1] [2] [3] [4] [5] [6] [7] Very easy

( ) [prefer not to answer]

For peer review only - http://bmjopen.bmj.com/site/about/guidelines.xhtml

For peer review only

2.5 …understand restrictions and recommendations of authorities regarding infectious diseases?

Very difficult [1] [2] [3] [4] [5] [6] [7] Very easy

( ) [prefer not to answer]

2.6 …follow the recommendations on how to protect yourself from infectious diseases?

Very difficult [1] [2] [3] [4] [5] [6] [7] Very easy

( ) [prefer not to answer]

2.7 …understand recommendations about when to stay at home from work/school, and when not to?

Very difficult [1] [2] [3] [4] [5] [6] [7] Very easy

( ) [prefer not to answer]

2.8 …follow recommendations about when to stay at home from work/school, and when not to?

Very difficult [1] [2] [3] [4] [5] [6] [7] Very easy

( ) [prefer not to answer]

2.9 …understand recommendations about when to engage in social activities, and when not to?

Very difficult [1] [2] [3] [4] [5] [6] [7] Very easy

( ) [prefer not to answer]

2.10…follow recommendations about when to engage in social activities, and when not to?

Very difficult [1] [2] [3] [4] [5] [6] [7] Very easy

( ) [prefer not to answer]

3. The following questions are about infectious disease-related knowledge and behaviors

3.1 What is the most effective measure for preventing measles? *(Do not read, check all that apply)*

1. Vaccination
2. Using medical masks
3. Avoiding contact with those who have measles
4. Other (specify)__________________________
5. Don't know
6. [prefer not to answer]

For peer review only - http://bmjopen.bmj.com/site/about/guidelines.xhtml

3.2 Please indicate whether you agree or disagree with the following statement:

- Antibiotics are effective in treating influenza.
  1. Agree
  2. Disagree
  3. Don't know
  4. [prefer not to answer]

3.3 Please indicate whether you agree or disagree with the following statement:

- Influenza is the same as common cold.
  1. Agree
  2. Disagree
  3. Don't know
  4. [prefer not to answer]

3.4 Please indicate whether you agree or disagree with the following statement:

- Influenza is caused by a virus.
  1. Agree
  2. Disagree
  3. Don't know
  4. [prefer not to answer]

3.5 Do you usually cover your mouth when coughing or sneezing in public places?

1. Yes
2. No
3. [prefer not to answer]

3.6 Do you usually wash your hands before eating?

1. Yes
2. No
3. [prefer not to answer]

4. The following questions are about the symptoms you might have experienced in the past.

4.1 Have you had fever (≥ 37) within the **last six months**?

( ) Yes→ Date (mm/yy) and the highest temperature, (C°):

____

( ) No

( ) Do not remember

( ) [prefer not to answer]

For peer review only - http://bmjopen.bmj.com/site/about/guidelines.xhtml

4.2 Have you had any respiratory symptoms (sore throat, cough, runny nose, shortness of breath, etc.) in the last 3 months?

( ) If yes, specify the date of the last episode → mm/yy: ____

( ) No → Move to section 12.4

( ) [prefer not to answer] → Move to section 12.4

4.3 Please indicate, if you have had any of the below-mentioned symptoms in the last 6 months?

Respiratory symptoms

	**Yes**	**No**	**[prefer not to answer]**
4.3.a. Sore throat	( )	( )	( )
4.3.b.Cough	( )	( )	( )
4.3.c. Runny nose	( )	( )	( )
4.3.d. Shortness of breath	( )	( )	( )

4.4. Other symptoms

	**Yes**	**No**	**[prefer not to answer]**
4.4.a. Muscle ache	( )	( )	( )
4.4.b. Joint ache	( )	( )	( )
4.4.c. Chills	( )	( )	( )
4.4.d. Rash	( )	( )	( )
4.4.e. Chest pain	( )	( )	( )
4.4.f Vomiting	( )	( )	( )
4.4.g Headache	( )	( )	( )
4.4.h Nausea	( )	( )	( )

For peer review only - http://bmjopen.bmj.com/site/about/guidelines.xhtml

	Yes	No	[prefer not to answer]
4.4.i Loss of taste	( )	( )	( )
4.4.g Loss of smell	( )	( )	( )
4.4.k. Loss of appetite	( )	( )	( )
4.4.l Abdominal pain	( )	( )	( )
4.4.m Diarrhea	( )	( )	( )
4.4.n Conjunctivitis	( )	( )	( )
4.4.o Nose bleed	( )	( )	( )
4.4.p Fatigue	( )	( )	( )
4.4.q Seizures	( )	( )	( )
4.4.r Altered consciousness	( )	( )	( )

4.5.a Other neurological signs

( ) Yes (specify): ____

( ) no

( ) [prefer not to answer]

4.5.b Other symptoms

( ) Yes (specify): ____

( ) No

( ) [prefer not to answer]

For peer review only

5. The following questions are about accompanying diseases and conditions.

Please indicate if you have had any of the below mentioned diseases/conditions.

5.1. Pregnancy

(Do not ask this question to males)

( ) Yes (specify the trimester: first, second, third, unknown):

____

( ) No

( ) [prefer not to answer]

*Read all of the options, marking **yes** or **no** in front of each. Do not read [prefer not to answer] option.*

	**Yes**	**No**	**Don't know/Refuse to answer**
5.2. Obesity	( )	( )	
5.3. Cancer	( )	( )	
5.4. Diabetes	( )	( )	
5.5. HIV/ other immune deficiency	( )	( )	
5.6. Heart disease	( )	( )	
5.7. Asthma (requiring medication)	( )	( )	
5.8. Chronic lung disease (non-asthma)	( )	( )	
5.9. Chronic liver disease	( )	( )	
5.10. Chronic hematological disorder	( )	( )	
5.11. Chronic kidney disease	( )	( )	
5.12. Chronic neurological impairment/disease	( )	( )	
5.13. Organ or bone marrow recipient	( )	( )	

For peer review only - http://bmjopen.bmj.com/site/about/guidelines.xhtml

For peer review only

5.14. Other accompanying disease(s)

( ) Yes (specify) ____

( ) No

( ) [prefer not to answer]

6. Have you smoked more than 100 cigarettes during your life?

( ) Yes

( ) No

( ) [prefer not to answer]

6.a How would you describe your current smoking status?

Read out all of the options

( ) I smoke everyday – go to question 16.b

( ) I smoke less than everyday

( ) I quit smoking less than a year ago

( ) I quit smoking more than a year ago

( ) [prefer not to answer]

6.b How many cigarettes do you smoke per day? __________

7. The following questions are about factors that might be associated with Hepatitis C and B infection in the population

7.1 How often do you visit the dentist to have your teeth cleaned, tooth extracted, braces put on, or root canals?

( ) Twice per year

( ) Once a year

( ) Once every 2 years

( ) Once every 3-5 years

( ) It has been more than 5 years

( ) Never

( ) Don't know/remember/[prefer not to answer]

For peer review only

For peer review only - http://bmjopen.bmj.com/site/about/guidelines.xhtml

7.2. Have you received any blood transfusions in your lifetime?

( ) Yes, before 199X
( ) Yes, in or after 199X
( ) No
( ) [prefer not to answer]

7.3 Do you have any tattoos?

( ) Yes
( ) No
( ) [prefer not to answer]

For peer review only

7.4 Have you ever been told by a doctor or other health worker that you had hepatitis B or C? *(multiple answers possible)*

( ) Yes hepatitis B
( ) Yes hepatitis C
( ) Yes, but don't know the type
( ) Neither hepatitis B nor hepatitis C
( ) I am vaccinated against hepatitis B
( ) Don't know/remember/refuse to answer

7.5 Have you ever been in prison (translation: long-term detainment) or jail (translation: short-term detainment)?

( ) Yes
( ) No
( ) [prefer not to answer]

End of interview

___:___ (hour:min)

Notes

____

Thank you!

For peer review only - http://bmjopen.bmj.com/site/about/guidelines.xhtml

STROBE Statement—Checklist of items that should be included in reports of ***cross-sectional studies***

	Item No	Recommendation	Page No
**Title and abstract**	1	(*a*) Indicate the study's design with a commonly used term in the title or the abstract	1
		(*b*) Provide in the abstract an informative and balanced summary of what was done and what was found	1
**Introduction**			
Background/rationale	2	Explain the scientific background and rationale for the investigation being reported	3-4
Objectives	3	State specific objectives, including any prespecified hypotheses	5
**Methods**			
Study design	4	Present key elements of study design early in the paper	5-6
Setting	5	Describe the setting, locations, and relevant dates, including periods of recruitment, exposure, follow-up, and data collection	5-6
Participants	6	(*a*) Give the eligibility criteria, and the sources and methods of selection of participants	6
Variables	7	Clearly define all outcomes, exposures, predictors, potential confounders, and effect modifiers. Give diagnostic criteria, if applicable	7-8
Data sources/ measurement	8*	For each variable of interest, give sources of data and details of methods of assessment (measurement). Describe comparability of assessment methods if there is more than one group	*7-8*
Bias	9	Describe any efforts to address potential sources of bias	6-7
Study size	10	Explain how the study size was arrived at	6
Quantitative variables	11	Explain how quantitative variables were handled in the analyses. If applicable, describe which groupings were chosen and why	9
Statistical methods	12	(*a*) Describe all statistical methods, including those used to control for confounding	9-10
		(*b*) Describe any methods used to examine subgroups and interactions	NA
		(*c*) Explain how missing data were addressed	10
		(*d*) If applicable, describe analytical methods taking account of sampling strategy	9
		(*e*) Describe any sensitivity analyses	NA
**Results**			
Participants	13*	(a) Report numbers of individuals at each stage of study—eg numbers potentially eligible, examined for eligibility, confirmed eligible, included in the study, completing follow-up, and analysed	10
		(b) Give reasons for non-participation at each stage	NA
		(c) Consider use of a flow diagram	NA
Descriptive data	14*	(a) Give characteristics of study participants (eg demographic, clinical, social) and information on exposures and potential confounders	11-14
		(b) Indicate number of participants with missing data for each variable of interest	11-14
Outcome data	15*	Report numbers of outcome events or summary measures	10-11

For peer review only

Main results	16	(*a*) Give unadjusted estimates and, if applicable, confounder-adjusted estimates and their precision (eg, 95% confidence interval). Make clear which confounders were adjusted for and why they were included	10
		(*b*) Report category boundaries when continuous variables were categorized	8
		(*c*) If relevant, consider translating estimates of relative risk into absolute risk for a meaningful time period	NA
Other analyses	17	Report other analyses done—eg analyses of subgroups and interactions, and sensitivity analyses	NA
**Discussion**			
Key results	18	Summarise key results with reference to study objectives	17
Limitations	19	Discuss limitations of the study, taking into account sources of potential bias or imprecision. Discuss both direction and magnitude of any potential bias	20
Interpretation	20	Give a cautious overall interpretation of results considering objectives, limitations, multiplicity of analyses, results from similar studies, and other relevant evidence	20-21
Generalisability	21	Discuss the generalisability (external validity) of the study results	18
**Other information**			
Funding	22	Give the source of funding and the role of the funders for the present study and, if applicable, for the original study on which the present article is based	21

*Give information separately for exposed and unexposed groups.

Note: An Explanation and Elaboration article discusses each checklist item and gives methodological background and published examples of transparent reporting. The STROBE checklist is best used in conjunction with this article (freely available on the Web sites of PLoS Medicine at http://www.plosmedicine.org/, Annals of Internal Medicine at http://www.annals.org/, and Epidemiology at http://www.epidem.com/). Information on the STROBE Initiative is available at www.strobe-statement.org.

For peer review only

BMJ Open

Prevalence of hepatitis B virus infection among general population of Armenia in 2021 and factors associated with it: a cross-sectional study

Journal:	*BMJ Open*
Manuscript ID	bmjopen-2023-080281.R2
Article Type:	Original research
Date Submitted by the Author:	22-Dec-2023

Complete List of Authors:	Demirchyan, Anahit; American University of Armenia, Turpanjian College of Health Sciences<br>Dudareva, S.; Robert Koch Institut, Department for Infectious Disease Epidemiology<br>Sahakyan, Serine; American University of Armenia, Turpanjian College of Health Sciences<br>Aslanyan, Lusine; American University of Armenia, Turpanjian College of Health Sciences<br>Muradyan, Diana; American University of Armenia, Turpanjian College of Health Sciences<br>Musheghyan, Lusine; American University of Armenia, Turpanjian College of Health Sciences<br>Mozalevskis, Antons; World Health Organization, Global HIV, Hepatitis and Sexually Transmitted Infections Programmes<br>Sargsyants, Narina; National Institute of Health named after academician Suren Avdalbekyan<br>Ghukasyan, Gayane; World Health Organization Country Office in Armenia<br>Petrosyan, V; American University of Armenia, Turpanjian College of Health Sciences

**Primary Subject Heading**:	Epidemiology
Secondary Subject Heading:	Infectious diseases
Keywords:	Epidemiology < INFECTIOUS DISEASES, Infection control < INFECTIOUS DISEASES, Public health < INFECTIOUS DISEASES

SCHOLARONE™
Manuscripts

BMJ

I, the Submitting Author has the right to grant and does grant on behalf of all authors of the Work (as defined in the below author licence), an exclusive licence and/or a non-exclusive licence for contributions from authors who are: i) UK Crown employees; ii) where BMJ has agreed a CC-BY licence shall apply, and/or iii) in accordance with the terms applicable for US Federal Government officers or employees acting as part of their official duties; on a worldwide, perpetual, irrevocable, royalty-free basis to BMJ Publishing Group Ltd ("BMJ") its licensees and where the relevant Journal is co-owned by BMJ to the co-owners of the Journal, to publish the Work in this journal and any other BMJ products and to exploit all rights, as set out in our licence.

The Submitting Author accepts and understands that any supply made under these terms is made by BMJ to the Submitting Author unless you are acting as an employee on behalf of your employer or a postgraduate student of an affiliated institution which is paying any applicable article publishing charge ("APC") for Open Access articles. Where the Submitting Author wishes to make the Work available on an Open Access basis (and intends to pay the relevant APC), the terms of reuse of such Open Access shall be governed by a Creative Commons licence – details of these licences and which Creative Commons licence will apply to this Work are set out in our licence referred to above.

Other than as permitted in any relevant BMJ Author's Self Archiving Policies, I confirm this Work has not been accepted for publication elsewhere, is not being considered for publication elsewhere and does not duplicate material already published. I confirm all authors consent to publication of this Work and authorise the granting of this licence.

For peer review only

Prevalence of hepatitis B virus infection among general population of Armenia in 2021 and factors associated with it: a cross-sectional study

Demirchyan, Anahit[1*]; Dudareva, Sandra[2]; Sahakyan, Serine[1]; Aslanyan, Lusine[1]; Muradyan, Diana[1]; Musheghyan, Lusine[1]; Mozalevskis, Antons[3]; Sargsyants, Narina[4]; Ghukasyan, Gayane[5]; Petrosyan, Varduhi[1]

[1] Turpanjian College of Health Sciences, American University of Armenia, Yerevan, Armenia

[2] Department for Infectious Disease Epidemiology, Robert Koch Institute, Berlin, Germany

[3] Global HIV, Hepatitis and Sexually Transmitted Infections Programmes, World Health Organization, Geneva, Switzerland

[4] National Institute of Health, Ministry of Health, Republic of Armenia, Yerevan, Armenia

[5] World Health Organization Country Office in Armenia, Yerevan, Armenia

[*] Corresponding author: Demirchyan, Anahit. Address: 40 Marshal Baghramyan ave., Yerevan 0019, Armenia. e-mail: ademirch@aua.am, telephone: +374 60 612562, Fax: +374 60 612512

Keywords: Hepatitis B virus infection; seroprevalence; Armenia; general adult population.

Word count: 4,389

For peer review only

For peer review only - http://bmjopen.bmj.com/site/about/guidelines.xhtml

Abstract

Objectives This study sought to determine the prevalence and associated factors of Hepatitis B virus (HBV) infection ever in life and chronic HBV infection in Armenia.

Design A population-based cross-sectional seroprevalence study combined with a phone survey of tested individuals.

Setting All administrative units of Armenia including ten provinces and capital city Yerevan.

Participants The study frame was the general adult population of Armenia aged ≥18 years.

Primary and secondary outcome measures The participants were tested for anti-HBV core antibodies (anti-HBc) and HBV surface antigen (HBsAg) using third-generation enzyme immunoassays. In case of HBsAg positivity, HBV DNA and hepatitis D virus (HDV) RNA PCR tests were performed. Risk factors of HBV infection ever in life (anti-HBc positivity) and chronic HBV infection (HBsAg positivity) were identified through fitting logistic regression models.

Results The seroprevalence study included 3838 individuals 18 years and older. Of them, 90.7% (3476 individuals) responded to the phone survey. The prevalence of anti-HBc positivity was 14.1% (95% CI 13.1%-15.2%) and HBsAg positivity 0.8% (95% CI 0.5%-1.1%). The viral load was over 10,000 IU/ml for 7.9% of HBsAg-positive individuals. None of the participants was positive for HDV. Risk factors for HBsAg positivity included less than secondary education (aOR=6.44; 95% CI 2.2-19.1), current smoking (aOR=2.56; 95% CI 1.2-5.6), and chronic liver disease (aOR=8.44; 95% CI 3.0-23.7). In addition to these, risk factors for anti-HBc positivity included age (aOR=1.04; 95% CI 1.04-1.05), imprisonment ever in life (aOR=2.53; 95% CI 1.41-4.56), and poor knowledge on infectious diseases (aOR=1.32; 95% CI 1.05-1.67), while living in Yerevan (vs. provinces) was protective (aOR=0.74; 95% CI 0.59-0.93).

Conclusion This study provided robust estimates of HBV markers among general population of Armenia. Its findings delineated the need to revise HBV testing and treatment strategies considering higher risk population groups, and improve population knowledge on HBV prevention.

For peer review only - http://bmjopen.bmj.com/site/about/guidelines.xhtml

STRENGTHS AND LIMITATIONS OF THIS STUDY

- ⇒ Rigorous sampling methodology applied among general adult population of Armenia insured the generalisability of the study findings.
- ⇒ Large sample size allowed to obtain robust prevalence estimates with narrow confidence intervals for HBV infection ever in life and chronic HBV infection.
- ⇒ Items on some important behavioral risk factors including injection drug use and risky sexual behaviors were not included in the questionnaire because of their sensitive nature.
- ⇒ The study methodology did not allow targeting specific population groups at high risk for HBV infection.

For peer review only

For peer review only - http://bmjopen.bmj.com/site/about/guidelines.xhtml

INTRODUCTION

Despite the availability of effective vaccination against hepatitis B virus (HBV), it continues to be a global threat to public health, particularly in low- and middle-income countries (LMIC).(1) According to World Health Organization (WHO) 2019 data, 296 million people in the globe live with chronic hepatitis B, and 1.5 million new infections occur annually.(1) More than a million fatalities globally are attributed to HBV, which is the primary cause of morbidity and mortality from chronic hepatitis, cirrhosis, and hepatocellular carcinoma (HCC), in addition to the acute infection itself.(2, 3)

The commonly known risk factors for HBV are being born to an infected mother, sharing injection syringes and needles, men having sex with men (MSM), female sex workers, living with HIV, and having occupational exposure to blood and blood products.(4-7) Additionally, studies have shown that sociodemographic factors including older age, male sex, low socio-economic status, poor housing, and low education could be risk factors for chronic HBV infection.(8, 9)

There has been a decline in the prevalence of chronic HBV infection in many parts of the world since a reliable vaccine became widely accessible more than 20 years ago.(10) Improved medical practices, such as screening of blood and blood products, applying safe injection techniques, and implementing infection control policies further contributed to this decline.(11)

A noticeable decrease in the incidence of acute viral hepatitis infections was observed in Armenia over the last three decades. The incidence of acute HBV infection steadily decreased from 22.30 in 1990 to 0.17 in 2021 per 100,000 population.(12) One of the explanations of this decrease is the high (over 96%) coverage of hepatitis B newborn and infant vaccination introduced in Armenia in 1999.(13)

Concerning the prevalence of chronic HBV infection, no nationally representative seroprevalence studies of HBV markers were conducted among general population of Armenia

For peer review only

before 2021. Based on synthesis of available screening data on blood donors, pregnant women, healthcare providers, and patients, the best guess was that chronic HBV infection was around 2% among general population of Armenia in 2000.(13) In 2013, based on retrospective testing of frozen blood samples of over 10,000 healthy individuals aged from 1 to 60 years, the median prevalence of HBV surface antigen (HBsAg) was 1.9-2.5%.(14, 15) In the meantime, the prevalence was reported as 1.4% among blood donors, 1.5% among pregnant women, 4.3% among prisoners, 6.6% among hemodialysis patients, 7.3% among healthcare workers, 8.8% among people who inject drugs, and 16.0% among patients with sexually transmitted infections.(15)

A recent review study that explored the groups of population at higher risk for chronic HBV infection in the countries of Caucasus and Central Asia, stated that chronic HBV infection was higher (3.7%) among prisoners in Armenia.(16) Concerning other vulnerable groups, the estimates varied across different small-scale studies from 0% to 1.6% for MSM and from 0.3% to 0.9% for migrants (mainly seasonal workers returning from Russia).(16)

For the purpose of assessing national disease prevention and control initiatives including immunization programs, knowledge of the region- and age-specific prevalence of hepatitis B infection is essential. The study presented in this paper was the first attempt to identify the seroprevalence of HBV infection among general adult population of Armenia. The study aimed to determine the prevalence of HBV infection ever in life and chronic HBV infection in Armenia and identify the risk factors of each of these outcomes among the general adult population.

METHODS

Study design

This study was a cross-sectional population-based sero-epidemiological study conducted throughout Armenia in May-September, 2021. The study included laboratory testing of blood

For peer review only - http://bmjopen.bmj.com/site/about/guidelines.xhtml

serum samples of the participants for biomarkers of hepatitis B, hepatitis C, and SARS-CoV-2 viruses, which was shortly followed with a telephone survey among them. The study was primarily designed to measure the biomarkers for SARS-CoV-2 and the hepatitis markers were added to it. The current paper reflects the analytical approaches and findings on HBV infection.

Sampling and sample size

The study frame was the general adult population of Armenia aged 18 years and over. We used a proportionate-to-the-population-size cluster sampling design, which included all 11 administrative units of Armenia (the ten provinces and Yerevan city). The study participants were recruited from primary healthcare (PHC) facilities, as the coverage of population with PHC is quite high in Armenia (about 97%). We selected one-third of the polyclinics in each administrative unit using random sampling – proportionate to the served population size. Simple random sampling was then applied in the national e-health operator "Armed" database to select the study participants from the lists of 18 years old and older individuals served by each chosen polyclinic.

The formula for estimating a proportion ($n = N*X / (X + N – 1)$, where $X = Z_{\alpha/2}{}^2 *p*(1-p) / (MOE)^2$) of the population was used to calculate the sample size for this study. When assuming 30% prevalence of anti-SARS-CoV-2 antibodies, 1.45% margin of error (MOE), type 1 error of 0.05, and confidence level of 95%, the calculated sample size was 3832. Assuming expected HBsAg prevalence of 2% and HBV core antibody (Anti-HBc) prevalence of 15%, this sample size allowed calculation of HBsAg prevalence estimate with a MOE of 0.44% and Anti-HBc prevalence estimate with a MOE of 1.13%.

Data collection

The "Armed" e-health system provided the list of selected unique ID numbers to the respective polyclinic's personnel trained for contacting the study participants. The personnel contacted each

For peer review only - http://bmjopen.bmj.com/site/about/guidelines.xhtml

potential participant by phone, following the script for the first contact, introduced the study and invited her/him to the polyclinic to take part in the study. When the participants came to the PHC facility, the research team obtained a written informed consent and collected blood samples. The participant recruitment in each polyclinic continued until the needed sample size was reached. Blood sampling was done by a trained nurse. Telephone interviews were carried out by trained interviewers within two weeks after the blood sampling. The structured questionnaire was administered using electronic tablets where Alchemer online tool was installed.

The study protocol was reviewed and approved by the Ethics Review Committee of the World Health Organization (protocol ID: CERC.0013C) and the Institutional Review Board of the American University of Armenia (protocol ID: AUA-2021-005), as conforming to the principles embodied in the Declaration of Helsinki. If the test result was positive, a trained hepatologist communicated the result to the individual and provided counseling. Negative results were sent to the participants via SMS messages.

Serum tests and definitions

Via venipuncture, 5-10 ml venous blood was collected from each participant and centrifuged immediately for the serum extraction. The serums were kept at 2-8°C for seven days at most before being tested at the laboratory of the National Center for Infectious Diseases of the Republic of Armenia. This laboratory was chosen because of having the needed technical and human resources to perform third generation serological and molecular testing with the required quality for all three infections included in the study. The testing procedure for HBV included the following steps: (1) all samples were tested for total anti-HBc antibodies using third-generation enzyme immunoassay (Elecsys® Anti-HBc II-Roche Diagnostics (17)); (2) if anti-HBc antibodies were detected, HBsAg test was performed (using Elecsys HBsAg-Roche Diagnostics (18)), followed by a confirmatory test if the result was questionable (within 0.9-1.0 COI); (3) if HBsAg test was positive, HBV DNA test was performed to test for the presence of HBV DNA

For peer review only - http://bmjopen.bmj.com/site/about/guidelines.xhtml

and the viral load (using Amplisens HBV Monitor FL (detection limit 150 IU/ml)); (4) all samples positive for HBsAg were also tested for hepatitis D virus (HDV) RNA (PCR test using Amplisens HDV-FL (detection limit 100 copies/ml)). All the applied tests were WHO pre-qualified. The presence of antibodies against HBV core antigen (anti-HBc antibodies) was viewed as serological evidence of HBV infection ever in life, while the presence of HBV surface antigen (HBsAg[1]) served as serological evidence of chronic HBV infection.

Survey instrument and study variables

The two outcome variables of this study were anti-HBc antibody status and HBsAg status measuring HBV infection ever in life and chronic HBV infection, respectively. The survey tool was a structured questionnaire which included items related to this study grouped in socioeconomic, health status, health literacy, and health behavioral domains. The sociodemographic domain included three continuous (age, family size, and socioeconomic status (SES) score) and four categorical (sex, education, employment, and residence) variables. The health status domain included only categorical (dichotomous) variables: hepatitis C exposure and chronic infection, several other self-reported chronic conditions (diabetes, chronic liver disease, obesity, heart disease, cancer, chronic kidney disease, and asthma requiring medication), as well as symptoms experienced during the six months prior to the survey (vomiting, fatigue, loss of appetite, joint ache, abdominal pain, fever, and nose bleeding). The health literacy domain included a communicable diseases knowledge variable measured by four questions and dichotomized at its lower third, and a score measuring the difficulty of understanding health-related information generated from responses to ten items. The health behavioral domain included the following categorical variables: ever and current regular smoking, having tattoos, ever having been imprisoned, ever receiving blood transfusion, frequency of visiting a dentist,

[1] The WHO surveillance case definition for chronic HBV infection assumes the presence of biomarkers of the infection combined with the absence of signs and symptoms of acute hepatitis. We applied this definition as coming across a recent infection in a cross-sectional population study is unlikely.

For peer review only - http://bmjopen.bmj.com/site/about/guidelines.xhtml

and self-reported hepatitis B vaccination status. A variable on ever having been told by a doctor of being infected with HBV was also included in the analysis. The questionnaire is provided as a supplementary file.

Patient and public involvement statement

The survey questionnaire was pretested among eligible people and refined based on their feedback. This was the only involvement of public in this research.

Data analysis

We used IBM SPSS Statistics for Windows, version 21 (IBM Corp., Armonk, N.Y., USA) for data management and analysis. All the analysis was weighted by sex and 5-year age groups to closely mimic age and sex distribution of the population of Armenia, as age and sex distribution in the study sample differed from that in the general population due to differences in response rates of different age and sex groups. To estimate the prevalence of HBV infection ever in life and chronic HBV infection, proportions and 95% confidence intervals were calculated for positivity to anti-HBc and HBsAg, respectively. To descriptively analyze independent variables, frequencies and proportions were calculated for categorical variables, and means and standard deviations for continuous variables in the total sample and in each of the outcome groups differentiated based on positivity/negativity to anti-HBc and positivity/negativity to HBsAg. Then univariate logistic regression analysis was done. The linearity of the association of continuous variables with each outcome variable was checked on the logit scale before entering them into logistic regression analysis.(19) The final step was fitting logistic regression models to identify factors independently associated to each of the outcomes. A series of multivariable logistic regression analyses were performed with inclusion of all those variables associated with the outcome at the $p < 0.25$ level in the univariate analysis.(19) The variables were entered into logistic regression analysis in different combinations using e*nter* method. The variables not

For peer review only - http://bmjopen.bmj.com/site/about/guidelines.xhtml

related to the outcome ($p \geq 0.10$) when controlling for the remaining variables in the model were excluded from the model, if their removal did not change the associations between the remaining variables and the outcome. The decision on keeping variables with a p-value between 0.05 and 0.10 in the model was made based on their potential relevance for interpretation of the data. The final models were checked for model fit and collinearity. Cases with any missing values were excluded from the fitted models.

RESULTS

Trained personnel from the selected polyclinics contacted a total of 9009 eligible individuals throughout the country to invite them for blood testing. Of them, 3831 (42.5%) individuals agreed and underwent blood testing. Of those tested, 3476 individuals (90.7%) participated in the phone survey. The prevalence numbers of HBV markers were generated from the sample that was tested (n=3831), but the variables needed for the rest of the analysis (descriptive comparisons and logistic regression) were available only for those 3476 individuals who participated in the phone survey and answered the survey questionnaire. Of them, 54.5% were women and 45.5% men. The mean age of women was 46.8 years (95% CI 46.0; 47.6), and the mean age of men 44.5 years (95% CI 43.6; 45.3). Of men, 43.9%, and of women, 41.0% had university or higher education, while 6.3% of men and 3.0% of women had lower than secondary education. Over one-third of the participants (36.3%) were from Yerevan and 63.7% from the provinces of Armenia. Further descriptive findings and comparisons between the groups with and without HBV markers are provided in Tables 1 and 2 and discussed later in the Results.

Prevalence of past and present HBV infection

The weighted percentage of those positive for anti-HBc antibodies indicating past or present HBV infection was 14.1% (95% CI 13.1%-15.2%). This percentage significantly increased with age (Figure 1) and was slightly higher among men than women (15.0% versus 12.2%, p=0.017).

For peer review only - http://bmjopen.bmj.com/site/about/guidelines.xhtml

The weighted percentage of those carrying HBsAg – the prevalence of chronic HBV infection – was 0.8% (95% CI 0.5%-1.1%). This estimate was the highest in the age group of 30-39 years old (1.5%, 95% CI 0.8%-2.6%) (Figure 1) and was higher among men compared to women (1.1% versus 0.5%, p=0.048). The measurement of the viral load among those positive for HBsAg showed that for 61.9% of them, HBV DNA was below 150 IU/ml, for 30.2% it was between 150 IU/ml and 2,000 IU/ml, and for 7.9% (n=2) – over 2,000 IU/ml (in the weighted analysis). For both these cases, the viral load was 11,000 IU/ml. For those HBV DNA-positive individuals with measurable (≥150 IU/ml) viral load, the mean level of HBV DNA was 2,787.6 IU/ml (95% CI 0.0; 5,925.6) and the interquartile range – 750 IU/ml. None of the HBsAg-positive samples were positive for HDV RNA.

Results of descriptive comparisons

Table 1 demonstrates the results of weighted crude comparisons of the study variables between the groups infected with HBV ever in life and those never infected with HBV.

Table 1 Distribution of selected variables by participants' anti-HBV core antibody status, Armenia, 2021

			Anti-HBV core antibody			
		N*	Positive (n=469)	Negative (n=3005)	*p-value*	Total (n=3474)
Socioeconomic variables:						
Age, mean (SD)		3473	57.1 (15.7)	44.0 (16.9)	*<0.001*	45.8 (17.3)
Socio-economic status score, mean (SD)		2696	46.8 (34.8)	50.3 (35.3)	*0.075*	49.9 (35.2)
Family size, mean (SD)		3451	4.2 (2.1)	4.4 (1.7)	*0.004*	4.4 (1.8)
Sex:	Female, %	3473	49.4	55.3	*0.017*	54.5
	Male, %		50.6	44.7		45.5
Education:	Below secondary, %	3456	7.7	4.0	*<0.001*	4.5
	Secondary/vocational, %		61.5	52.0		53.3
	University/higher, %		30.8	44.0		42.3
Employment:	Employed/student, %	3438	41.8	58.6	*<0.001*	56.4
	Unemployed/seasonal migrant, %		27.2	28.9		28.7
	Retired/disabled, %		30.9	12.5		15.0
Residence:	Yerevan city, %		28.6	37.4	*<0.001*	36.3
	Provinces, %		71.4	62.6		63.7
Health status variables:						
Anti-Hepatitis C antibodies positive, %		3474	3.6	1.7	*0.011*	2.0

For peer review only - http://bmjopen.bmj.com/site/about/guidelines.xhtml

	N*	Anti-HBV core antibody Positive (n=469)	Negative (n=3005)	*p-value*	Total (n=3474)
Hepatitis C PCR test positive, %	3474	1.5	0.6	*0.034*	0.7
Diabetes, %	3395	12.4	7.1	*<0.001*	7.8
Chronic liver disease, %	3391	8.4	2.1	*<0.001*	2.9
Obesity, %	3383	13.8	9.5	*0.007*	10.1
Heart disease, %	3390	29.0	12.6	*<0.001*	14.8
Cancer, %	3394	2.0	1.5	*0.408*	1.5
Chronic kidney disease, %	3395	4.6	3.6	*0.286*	3.7
Asthma requiring medication, %	3391	2.0	2.3	*0.864*	2.2
Symptoms experienced during the last six months:					
Vomiting, %	3387	3.3	4.6	*0.268*	4.5
Fatigue, %	3391	41.8	46.0	*0.104*	45.4
Loss of appetite, %	3388	8.9	14.4	*0.001*	13.6
Joint ache, %	3393	37.6	33.4	*0.078*	33.9
Abdominal pain, %	3382	9.4	11.2	*0.257*	11.0
Fever, %	3395	16.5	25.4	*<0.001*	24.2
Nose bleed, %	3387	2.9	4.6	*0.108*	4.4
Health literacy variables:					
Poor knowledge on infectious diseases, %	3474	32.8	24.3	*<0.001*	25.5
Health information understanding score, mean (SD)	3005	52.2 (11.5)	54.4 (9.9)	*<0.001*	54.1 (10.1)
Health behavioral variables:					
Current smoking, %	3389	25.7	20.7	*0.016*	21.4
Ever smoking, %	3395	39.5	34.0	*0.026*	34.7
Having tattoos, %	3380	13.0	10.0	*0.055*	10.4
Undergoing blood transfusion, %	3380	4.0	3.4	*0.487*	3.5
Ever having been imprisoned, %	3387	4.9	1.2	*<0.001*	1.7
Visiting a dentist: Once in 3-5 years/more, %	3329	52.5	74.3	*<0.001*	71.4
Less frequently/never, %		47.5	25.7		28.6
Vaccinated against Hepatitis B (self-reported), %	3397	0.2	0.2	*1.000*	0.2
Hepatitis B told by doctor, %	3397	2.7	0.2	*<0.001*	0.5

N, Number of valid responses; HBV, Hepatitis B virus; PCR, Polymerase chain reaction; SD, Standard deviation.
* Weighted estimates; *p-value:* two-sided.

For peer review only

The ever-infected group was older, with lower level of education, from a smaller-size family, and more often – residing in provinces. The proportion of employed was lower among ever infected group and the proportion of retired/disabled was higher. Anti-hepatitis C antibodies were found among the ever-infected group more frequently. The ever-infected group more frequently reported some chronic health conditions – diabetes, chronic liver disease, obesity, and heart disease. However, some symptoms experienced during the last six months, including loss

For peer review only - http://bmjopen.bmj.com/site/about/guidelines.xhtml

of appetite and abdominal pain, were less frequently reported by the ever-infected group. The ever-infected group demonstrated poor knowledge on infectious diseases. The mean score of understanding health-related information was also lower among them compared to the never infected group. There were significant differences between the groups in the health behavioral domain as well. Both current and ever smokers were more prevalent among the ever infected. Higher proportion of them were ever imprisoned, and lower proportion used dental services at least once in 3-5 years. A similar negligible proportion of respondents (0.2%) in both groups reported having been vaccinated against hepatitis B.

The characteristics of those participants with and without chronic HBV infection were compared in Table 2.

Table 2 Distribution of selected variables by Hepatitis B surface antigen (HBsAg) status, Armenia, 2021

			HBsAg			
		N*	Positive (n=27)	Negative (n=3447)	*p-value*	Total (n=3474)
Socioeconomic variables:						
Age, mean (SD)		3473	44.7 (11.9)	45.8 (17.4)	*0.734*	45.8 (17.3)
Socio-economic status score, mean (SD)		2696	43.7 (24.3)	49.9 (35.3)	*0.471*	49.9 (35.2)
Family size, mean (SD)		3451	4.2 (1.3)	3.4 (1.8)	*0.595*	4.4 (1.8)
Sex:	Female, %	3473	40.7	54.7	*0.048*	54.5
	Male, %		65.4	45.3		45.5
Education:	Below secondary, %	3455	22.2	4.3	*<0.001*	4.5
	Secondary/vocational, %		44.4	53.3		53.3
	University/higher, %		33.3	42.3		42.3
Employment:	Employed/student, %	3439	48.1	56.4	*0.607*	56.4
	Unemployed/seasonal migrant, %		37.0	28.6		28.7
	Retired/disabled, %		14.8	15.0		15.0
Residence:	Yerevan city, %		29.6	36.3	*0.551*	36.3
	Provinces, %		70.4	63.7		63.7
Health status variables:						
Anti-Hepatitis C antibodies positive, %		3474	7.4	1.9	*0.097*	2.0
Hepatitis C PCR test positive, %		3474	3.7	0.7	*0.171*	0.7
Diabetes, %		3396	11.1	7.8	*0.464*	7.8
Chronic liver disease, %		3390	19.2	2.8	*0.001*	2.9
Obesity, %		3384	14.8	10.0	*0.343*	10.1
Heart disease, %		3389	14.8	14.8	*1.000*	14.8
Cancer, %		3395	0.0	1.5	*1.000*	1.5
Chronic kidney disease, %		3395	3.7	3.7	*1.000*	3.7
Asthma requiring medication, %		3391	3.7	2.2	*0.459*	2.2
Symptoms experienced during the last six months:						

For peer review only - http://bmjopen.bmj.com/site/about/guidelines.xhtml

	N*	HBsAg Positive (n=27)	HBsAg Negative (n=3447)	*p-value*	Total (n=3474)
Vomiting, %	3388	0.0	4.5	*0.632*	4.5
Fatigue, %	3392	40.7	45.5	*0.700*	45.4
Loss of appetite, %	3388	0.0	13.7	*0.042*	13.6
Joint ache, %	3394	48.1	33.8	*0.151*	33.9
Abdominal pain, %	3382	4.2	11.0	*0.508*	11.0
Fever, %	3395	11.1	24.3	*0.173*	24.2
Nose bleed, %	3387	0.0	4.4	*0.630*	4.4
Health literacy variables:					
Poor knowledge on infectious diseases, %	3474	37.0	25.4	*0.183*	25.5
Health information understanding score, mean (SD)	3005	48.7 (14.4)	54.2 (10.1)	*0.016*	54.1 (10.1)
Health behavioral variables:					
Current smoking, %	3390	44.4	21.2	*0.007*	21.4
Ever smoking, %	3394	53.8	34.6	*0.060*	34.7
Having tattoos, %	3379	19.2	10.3	*0.182*	10.4
Undergoing blood transfusion, %	3380	3.7	3.5	*0.615*	3.5
Ever having been imprisoned, %	3387	11.6	1.6	*0.010*	1.7
Visiting a dentist: Once in 3-5 years/more, %	3330	74.1	71.4	*0.834*	71.4
Less frequently/never, %		25.9	28.6		28.6
Vaccinated against Hepatitis B (self-reported), %	3398	0.0	0.2	*1.000*	0.2
Hepatitis B told by doctor, %	3398	22.2	0.4	*<0.001*	0.5

N, Number of valid responses; PCR, Polymerase chain reaction; SD, Standard deviation.
* Weighted estimates; *p-value:* two-sided.

Those chronically infected had lower education and higher composition of male. They reported chronic liver disease much more frequently. The proportion of those demonstrating poor knowledge on infectious diseases was higher among them and the mean score of understanding health-related information was lower. Smoking was more frequent among them, and they have been imprisoned much more frequently than those free from the virus.

Among those testing positive for anti-HBc, 12 (2.7%) reported that they have ever been told by a doctor that they were infected with hepatitis B. Six of these 12 persons were also positive for HBsAg, thus only 22.2% of those HBsAg positive were aware of their status (Tables 1, 2).

Findings of regression analysis

The fitted logistic regression model of factors independently associated with ever being infected with HBV included the following characteristics – age, education, Yerevan vs. province

For peer review only - http://bmjopen.bmj.com/site/about/guidelines.xhtml

residence, chronic liver disease, current smoking, imprisonment ever in life, and knowledge on infectious diseases (Table 3).

Table 3 Multivariable logistic regression model of determinants of HBV infection ever in life (positivity for anti-HBV core antibody) among adult population in Armenia, 2021 (valid N=3349)

Characteristics		aOR	95% CI	*p- value*
Age, years		1.04	1.04 - 1.05	< 0.001
Education:	Less than secondary	1.61	1.02 - 2.56	0.042
	Secondary/vocational	1.26	1.00 - 1.59	0.052
	University/higher	1.00	ref.	ref.
Living in Yerevan vs. provinces		0.74	0.59 - 0.93	0.011
Chronic liver disease		2.93	1.88 - 4.56	<0.001
Current smoking		1.60	1.25 - 2.06	<0.001
Ever imprisoned		2.53	1.41 - 4.56	0.002
Poor knowledge on infectious diseases		1.32	1.05 - 1.67	0.018

HBV, hepatitis B virus.
N, number of cases.
aOR, adjusted odds ratio.
CI, confidence interval.

When holding all the other variables constant, each year increase in age increased the likelihood of ever being infected with HBV by 4%. Compared to having university/higher education, having less than secondary education was associated with 1.61 times higher odds, and having secondary/vocational education with 1.26 times higher odds of ever being infected with HBV. Residing in Yerevan was protective – 0.74 times decreasing the odds of HBV infection ever in life. Suffering from chronic liver disease was associated with 2.93 times higher odds of HBV infection ever in life. Ever being imprisoned increased the odds of HBV infection ever in life 2.53 times. Current smoking was also a risk factor for ever having HBV infection, increasing its odds 1.60 times. Poor knowledge of infectious diseases was associated with 1.32 times higher odds of having HBV infection ever in life. The model had no collinearity issues with all Variance Inflation Factor (VIF) values being below 1.13.

For peer review only - http://bmjopen.bmj.com/site/about/guidelines.xhtml

Several factors associated with having HBV infection ever in life, including education, chronic liver disease, and current smoking, were also significant in the fitted model of determinants of chronic HBV infection (Table 4).

Table 4 Multivariable logistic regression model of determinants of chronic HBV infection (HBsAg positivity) among adult population of Armenia, 2021 (valid N=3349)

Characteristics		aOR	95% CI	*p- value*
Education:	Less than secondary	6.44	2.17 - 19.12	<0.001
	Secondary/vocational	1.01	0.42 - 2.45	0.984
	University/higher	1.00	ref.	ref.
Chronic liver disease		8.44	3.00 - 23.72	<0.001
Current smoking		2.56	1.16 - 5.65	0.019
Ever imprisoned		3.33	0.81 - 13.74	0.096

HBV, hepatitis B virus.
HBsAg, Hepatitis B surface antigen.
N, number of cases.
aOR, adjusted odds ratio.
CI, confidence interval.

Compared to secondary or higher education, less than secondary education increased the odds of having chronic HBV infection 6.4 times. Chronic liver disease was associated with 8.4 times higher odds of having chronic HBV infection. Current smoking increased the likelihood of chronic HBV infection 2.6 times. Ever having been imprisoned was also potentially associated with having chronic HBV infection, as the observed effect range between these variables was suggestive of a weak negative to a strong positive association (aOR 3.33; 95% CI 0.81 - 13.74). Again, no collinearity issues were identified in this model with all VIF values being below 1.06.

DISCUSSION

This is the first population-based seroprevalence study to estimate the nationwide prevalence of HBV infection in Armenia. The study demonstrated that the proportion of adult population ever infected with hepatitis B was considerably high in Armenia (14.1%; 95% CI 0.5%,1.1%). Yet, the prevalence of chronic HBV infection was low (0.8%; 95% CI 13.1%, 15.2%), and none of

For peer review only - http://bmjopen.bmj.com/site/about/guidelines.xhtml

the HBsAg-positive participants were positive for HDV RNA. The identified prevalence in Armenia was lower than in a study carried out in the neighboring Georgia in 2021: the prevalence of chronic hepatitis B among adults in Georgia was 2.7% (95% CI 2.3%, 3.4%) and the proportion of persons ever infected with hepatitis B was 21.7% (95% CI 20.4%, 23.2%).(20). Previous data for Armenia based on modeling studies showed a higher prevalence of chronic HBV infection. According to a modeling study from 2016, the HBsAg prevalence in Armenia was estimated at 1.9%,(21) and based on the Coalition for Global Hepatitis Elimination estimates, it was gradually decreasing from 3.4% in 1994/95.(22) Surveillance data confirm that there has been a continuous reduction of new cases – mainly explained by introduction of hepatitis B vaccination among children in 1999.(13, 15) This will likely contribute to further decrease in prevalence of chronic HBV infection in Armenia.(23) The prevalence in 2016 was probably an overestimation as it was relying on proxy data and not a measurement attributable to general population in Armenia. Data from screening of pregnant women in Armenia support the current finding of low prevalence. For the years 2017-2021, from over 97,000 pregnant women that underwent screening, 0.66% were HBsAg positive.(24)

In crude weighted comparisons, the prevalence of both HBsAg and anti-HBc were higher in males than in females, which is similar to other studies.(20, 25, 26) Generally, difference in the prevalence between men and women may be related to either behavioral factors like higher sexual risk behaviors, intravenous drug use and imprisonment in men, or to biological factors.(27) Based on studies, it has been discussed that females usually develop more intense innate, humoral and cellular responses to viral infections, including hepatitis virus, than males.(27, 28) After controlling for other variables, this association, however, disappeared in the model.

While for ever being infected with HBV, it was possible to detect a statistically significant increase in the prevalence with increasing age, there were no detectable differences with

For peer review only - http://bmjopen.bmj.com/site/about/guidelines.xhtml

increasing age in the prevalence of chronic hepatitis B. However, the analysis of HBsAg prevalence by different groups was limited by low number of positive cases and, correspondingly, lack of power with the given sample size. Descriptive results, however, show that there were no chronic hepatitis B cases among those 18-29 years of age. Individuals up to age of 22 have been born after the introduction of the birth dose and childhood hepatitis B vaccination in Armenia. This potentially leads to less cases in this age group. According to the reports submitted to WHO, the impact of hepatitis B vaccination on the control of mother to child transmission has been demonstrated in several countries of the WHO European Region with historically intermediate and high hepatitis B endemicity, like Republic of Moldova, Georgia, Kyrgyzstan, Uzbekistan, Tajikistan and Turkmenistan.(23, 29) We also did not find any HBsAg positive cases among those 70 years and older, while the percentage positive for anti-HBc was the highest in this group. On one hand, those with persistent chronic infection might have already had a lethal outcome at that age, on other hand, the sample size in this age group (n=274) might have been a limiting factor to detect prevalent cases. Furthermore, the HBsAg clearance rate with and without therapy might also influence this figure.(30) Several other factors associated with higher odds for current or past infection were identified in the multivariable regression models. For both ever being infected and chronic infection, odds were higher among people with less than secondary education and current smokers. Odds for ever being infected were higher among persons living in provinces (versus Yerevan), those demonstrating poorer knowledge on infectious diseases, and those ever imprisoned. Individuals with lower levels of education, smoking, and individuals who have been imprisoned might be more likely to engage in riskier behavior such as drug use and unprotected sexual activity leading to increased exposure to HBV.(31-33) Furthermore, lower education levels and socioeconomic disadvantages can result in reduced access to healthcare services and prevention.(34, 35) Higher odds for ever being infected with HBV in provinces compared to Yerevan could be as well related to historically

For peer review only - http://bmjopen.bmj.com/site/about/guidelines.xhtml

poorer access to health care or lower level of infection prevention and control measures in regional heathcare centers. Though, there is no documented evidence supporting this assumption. We could also demonstrate that poorer knowledge about infectious diseases was associated with higher odds for detection of anti-HBc, possibly explained by poorer knowledge about transmission routes, limited understanding of preventive measures, misconceptions and stigma, and poorer access to health related information.(33, 36, 37)

In 7.9% of chronic infections (n=2 in the weighted analysis), the viral load was above 10,000 IU/ml, indicating the need for treatment.(38) A viral load above 10,000 IU/ml is a strong risk predictor of HCC, independent of HBeAg status, ALT level and liver cirrhosis.(39-43) The results on viral load suggest that there is a tangible proportion of chronic hepatitis B patients in Armenia that are currently not receiving antiviral treatment or were not aware of their infection. As the testing and treatment costs in case of a chronic HBV infection are currently mainly related to out-of-pocket payments in Armenia, the detection of cases and sustaining them in care is hindered.(24).

Based on our prevalence findings (0.8%) and given the adult population size of Armenia (~2,500,000), the estimated number of people living with chronic HBV infection in Armenia could be around 20,000, and 1,600 of them (7.9% with a viral load above 10,000 IU/ml) could be at high risk of HCC and in need for immediate treatment. Yet, roughly, only one-fifth of chronically infected are aware of their infection. The WHO target for 2030 is 90% of people living with hepatitis B diagnosed, which calls for improved access to testing to reach the global and regional goals.(44) Hence, there is a need to revise testing and treatment strategies in Armenia in order to find and timely treat chronic hepatitis B cases.

Limitations: Due to limited length of the questionnaire we were not able to include all relevant questions to determine other factors associated with higher odds of being infected with HBV. Also, as the study was conducted during the COVID-19 pandemic, some of the reported

For peer review only - http://bmjopen.bmj.com/site/about/guidelines.xhtml

symptoms experienced within six months prior to the survey could be related to COVID disease rather than participant's general health status. Furthermore, as the sample size was calculated for the SARS-CoV-2 study needs, our possibilities for calculation of the prevalence of HBV infection for different groups were limited and we might lack power to detect differences among groups and when performing regression analyses. At the same time, the sample size was appropriate to measure nationwide robust estimates with narrow confidence intervals.

RECOMMENDATIONS

In order to diagnose persons living with HBV, testing should be scaled up, especially among the population groups that are likely more affected. Improvement of state-funded preventive and testing strategies and warranting the access to state-funded hepatitis B treatment is needed in order to find and timely treat cases to prevent sequel like cirrhosis and HCC, and decrease mortality. Furthermore, to reach elimination of hepatitis B, prevention of mother-to-child transmission should be maintained at high level.(45) Validation of reaching hepatitis B control targets with a study targeting younger children (from the vaccinated cohort) could be considered in Armenia.

Also, population groups at high risk for HBV infection due to behavioral or socio-economic reasons should be targeted with preventive measures including information campaigns, vaccination and access to needle-syringe programs. Actions to improve knowledge on hepatitis B transmission routes, prevention and testing are needed for all population groups.

CONCLUSIONS

This is the first nation-wide representative study in Armenia providing robust estimates of anti-HBc and HBsAg prevalence among general population. While the prevalence of chronic hepatitis B is rather low in Armenia, the proportion of persons ever been infected with HBV is

For peer review only - http://bmjopen.bmj.com/site/about/guidelines.xhtml

considerable. Age distribution of HBV infection ever in life suggests that there has been historically high incidence and that new infections possibly still occur among adults. Meanwhile, the undetectable prevalence among younger age groups suggests the positive impact of the hepatitis B vaccination.

Acknowledgements This work was done with partial support from WHO Regional Office for Europe and technical assistance from Robert Koch Institute. The authors alone are responsible for the views expressed in this article and they do not necessarily represent the views, decisions, or policies of the institutions with which they are affiliated.

Contributors AD, SS, LM, AM, NS, GG and VP conceptualized this study, AD performed the analysis. AD, SD, LA, and DM drafted the manuscript. All authors supported the interpretation of the study results and critically reviewed and revised the manuscript.

Competing interests None declared.

Funding The data collection was partially funded by WHO Regional Office for Europe (no grant number). No funding was received for developing this manuscript.

Data availability statement Data are available on reasonable request.

Ethics Approval Statement The study protocol was reviewed and approved by the Ethics Review Committee of the World Health Organization (protocol ID: CERC.0013C) and the Institutional Review Board of the American University of Armenia (protocol ID: AUA-2021-005), as conforming to the principles embodied in the Declaration of Helsinki.

For peer review only - http://bmjopen.bmj.com/site/about/guidelines.xhtml

REFERENCES

1. G. B. D. Hepatitis B Collaborators. Global, regional, and national burden of hepatitis B, 1990-2019: a systematic analysis for the Global Burden of Disease Study 2019. Lancet Gastroenterol Hepatol. 2022;7(9):796-829. doi: 10.1016/S2468-1253(22)00124-8

2. Purcell RH. The discovery of the hepatitis viruses. Gastroenterology. 1993;104(4):955-63. doi: 10.1016/0016-5085(93)90261-a

3. Tseng TC, Liu CJ, Yang HC, et al. High levels of hepatitis B surface antigen increase risk of hepatocellular carcinoma in patients with low HBV load. Gastroenterology. 2012;142(5):1140-9 e3; quiz e13-4. doi: 10.1053/j.gastro.2012.02.007

4. Zhao X, Shi X, Lv M, et al. Prevalence and factors associated with hepatitis B virus infection among household members: a cross-sectional study in Beijing. Hum Vaccin Immunother. 2021;17(6):1818-24. doi: 10.1080/21645515.2020.1847951

5. Karoney MJ, Some FF, Dip HIL, et al. Burden of Hepatitis B Infection among High Risk Populations in Western Kenya. J Infect Dis Epidemiol 2020;6:132. doi: 10.23937/2474-3658/1510132

6. Muljono DH, Wijayadi T, Sjahril R. Hepatitis B Virus Infection among Health Care Workers in Indonesia. Euroasian J Hepatogastroenterol. 2018;8(1):88-92. doi: 10.5005/jp-journals-10018-1269

7. Hou J, Liu Z, Gu F. Epidemiology and Prevention of Hepatitis B Virus Infection. Int J Med Sci. 2005;2(1):50-7. doi: 10.7150/ijms.2.50

8. Gnyawali B, Pusateri A, Nickerson A, et al. Epidemiologic and socioeconomic factors impacting hepatitis B virus and related hepatocellular carcinoma. World J Gastroenterol. 2022;28(29):3793-802. doi: 10.3748/wjg.v28.i29.37934.

For peer review only

For peer review only - http://bmjopen.bmj.com/site/about/guidelines.xhtml

9. Tosun S, Aygun O, Ozdemir HO, et al. The impact of economic and social factors on the prevalence of hepatitis B in Turkey. BMC Public Health. 2018;18(1):649. doi: 10.1186/s12889-018-5575-6

10. Pattyn J, Hendrickx G, Vorsters A, et al. Hepatitis B Vaccines. J Infect Dis. 2021;224(12 Suppl 2):S343-S51. doi: 10.1093/infdis/jiaa668

11. Ott JJ, Stevens GA, Groeger J, et al. Global epidemiology of hepatitis B virus infection: new estimates of age-specific HBsAg seroprevalence and endemicity. Vaccine. 2012;30(12):2212-9. doi: 10.1016/j.vaccine.2011.12.116

12. Andreasyan D, Bazarchyan A. "Health and Health Care" Yearbook. Yerevan: National Institute of Health Named after Academician S. Avdalbekyan; 2022. 299 p.

13. Demirchyan A, Mirzoyan L, Thompson ME. Synthesis of the existing data on Hepatitis B in Armenia. Yerevan: American University of Armenia, Center for Health Services Research; 2000. https://aua.am/chsr/PDF/2000/HepatitB_FINAL.pdf

14. Ghazinyan H, Mkhitaryan A, Melik-Andreasyan G, et al. Updating HBV status in Armenia. Optimal Management of Hepatitis B Virus Infection; 25–27 September Athens, Greece: Proceedings of EASL Special Conference; 2014. p. 159.

15. Melik-Andreasyan GG, Markosyan IE, Voskanyan LS, et al. Epidemiological Characteristics of Viral Hepatitis in Armenia (in Russian). В мире вирусных гепатитов. 2013;4:20-6.

16. Davlidova S, Haley-Johnson Z, Nyhan K, et al. Prevalence of HIV, HCV and HBV in Central Asia and the Caucasus: A systematic review. Int J Infect Dis. 2021;104:510-25. doi: 10.1016/j.ijid.2020.12.068

17. Roche Diagnostics. Elecsys® Anti-HBc II. Immunoassay for the qualitative determination of total antibodies against hepatitis B core antigen (HBcAg) [Available from: https://diagnostics.roche.com/global/en/products/params/elecsys-anti-hbc-ii.html].

For peer review only - http://bmjopen.bmj.com/site/about/guidelines.xhtml

18. Roche Diagnostics. Elecsys® HBsAg II. Immunoassay for the qualitative determination of hepatitis B surface antigen (HBsAg) [Available from: https://diagnostics.roche.com/global/en/products/params/elecsys-hbsag-ii.html].

19. Hosmer DW, Lemeshow S. Applied logistic regression. 2nd ed. New York: A Wiley-Interscience Publication; 2000: 92-116.

20. Khetsuriani N, Gamkrelidze A, Shadaker S, et al. Hepatitis B prevalence and the impact of vaccination in Georgia: results from a nationwide serosurvey International Liver Congress TM 2022, London22-26 June 2022 [Available from: https://www.postersessiononline.eu/173580348_eu/congresos/ILC2022/aula/-THU_283_ILC2022.pdf].

21. Polaris Observatory C. Global prevalence, treatment, and prevention of hepatitis B virus infection in 2016: a modelling study. Lancet Gastroenterol Hepatol. 2018;3(6):383-403. doi: 10.1016/S2468-1253(18)30056-6

22. Coalition for Global Hepatitis Elimination. Data Dashboard Armenia. [Available from: https://www.globalhep.org/country-progress/armenia] (accessed 27 July 2023).

23. Khetsuriani N, Tishkova F, Jabirov S, et al. Substantial decline in hepatitis B virus infections following vaccine introduction in Tajikistan. Vaccine. 2015;33(32):4019-24. doi: 10.1016/j.vaccine.2015.05.092

24. Robert Koch Institute. Report: Technical Assessment of Viral Hepatitis Response in Armenia. Berlin; 2023: 16-19.

25. Khan NU, Zalan A, Petruzziello A, et al. Determining the Actual Prevalence of Hepatitis B in Khyber Pakhtunkhwa-Pakistan: A Meta-Analysis. Open Virol J. 2018;12:33-41. doi: 10.2174/1874357901812010033

For peer review only - http://bmjopen.bmj.com/site/about/guidelines.xhtml

26. Wang H, Men P, Xiao Y, et al. Hepatitis B infection in the general population of China: a systematic review and meta-analysis. BMC Infect Dis. 2019;19(1):811. doi: 10.1186/s12879-019-4428-y

27. Brown R, Goulder P, Matthews PC. Sexual Dimorphism in Chronic Hepatitis B Virus (HBV) Infection: Evidence to Inform Elimination Efforts. Wellcome Open Res. 2022;7:32. doi: 10.12688/wellcomeopenres.17601.2

28. Ruggieri A, Gagliardi MC, Anticoli S. Sex-Dependent Outcome of Hepatitis B and C Viruses Infections: Synergy of Sex Hormones and Immune Responses? Front Immunol. 2018;9:2302. doi: 10.3389/fimmu.2018.02302

29. More countries reaching hepatitis B control targets brings the WHO European Region closer to eliminating viral hepatitis as a public health threat [press release]. https://www.who.int/europe/news/item/24-04-2023-more-countries-reaching-hepatitis-b-control-targets-brings-the-who-european-region-closer-to-eliminating-viral-hepatitis-as-a-public-health-threat2023 (accessed 25 April 2023).

30. Terrault NA, Wahed AS, Feld JJ, et al. Incidence and prediction of HBsAg seroclearance in a prospective multi-ethnic HBeAg-negative chronic hepatitis B cohort. Hepatology. 2022;75(3):709-23. doi: 10.1002/hep.32231

31. Galea S, Vlahov D. Social determinants and the health of drug users: socioeconomic status, homelessness, and incarceration. Public Health Rep. 2002;117 Suppl 1(Suppl 1):S135-45.

32. MacLachlan JH, Cowie BC. Hepatitis B virus epidemiology. Cold Spring Harb Perspect Med. 2015;5(5):a021410. doi: 10.1101/cshperspect.a021410

33. Corona R, Caprilli F, Giglio A, et al. Risk factors for hepatitis B virus infection among heterosexuals attending a sexually transmitted diseases clinic in Italy: role of genital ulcerative diseases. J Med Virol. 1996;48(3):262-6. doi: 10.1002/(SICI)1096-9071(199603)48:3<262::AID-JMV8>3.0.CO;2-6

For peer review only

For peer review only - http://bmjopen.bmj.com/site/about/guidelines.xhtml

34. McMaughan DJ, Oloruntoba O, Smith ML. Socioeconomic Status and Access to Healthcare: Interrelated Drivers for Healthy Aging. Front Public Health. 2020;8:231. doi: 10.3389/fpubh.2020.00231

35. Brodzinski A, Neumeyer-Gromen A, Dudareva S, et al. [Hepatitis B virus infection and vaccine-induced immunity: the role of sociodemographic determinants: Results of the study "German Health Interview and Examination Survey for Adults" (DEGS1, 2008-2011)]. Bundesgesundheitsblatt Gesundheitsforschung Gesundheitsschutz. 2022;65(2):159-69. doi: 10.1007/s00103-021-03473-z

36. Machmud PB, Glasauer S, Gottschick C, et al. Knowledge, Vaccination Status, and Reasons for Avoiding Vaccinations against Hepatitis B in Developing Countries: A Systematic Review. Vaccines (Basel). 2021;9(6). doi: 10.3390/vaccines9060625

37. Pereira LM, Martelli CM, Merchan-Hamann E, et al. Population-based multicentric survey of hepatitis B infection and risk factor differences among three regions in Brazil. Am J Trop Med Hyg. 2009;81(2):240-7.

38. European Association for the Study of the Liver. EASL 2017 Clinical Practice Guidelines on the management of hepatitis B virus infection. J Hepatol. 2017;67(2):370-98. doi: 10.1016/j.jhep.2017.03.021

39. Chen CJ, Yang HI, Su J, et al. Risk of hepatocellular carcinoma across a biological gradient of serum hepatitis B virus DNA level. JAMA. 2006;295(1):65-73. doi: 10.1001/jama.295.1.65

40. Liu CJ, Chen BF, Chen PJ, et al. Role of hepatitis B viral load and basal core promoter mutation in hepatocellular carcinoma in hepatitis B carriers. J Infect Dis. 2006;193(9):1258-65. doi: 10.1086/502978

41. Barcena Marugan R, Garcia Garzon S. DNA-guided hepatitis B treatment, viral load is essential, but not sufficient. World J Gastroenterol. 2009;15(4):423-30. doi: 10.3748/wjg.15.423

For peer review only - http://bmjopen.bmj.com/site/about/guidelines.xhtml

42. Mendy ME, Welzel T, Lesi OA, et al. Hepatitis B viral load and risk for liver cirrhosis and hepatocellular carcinoma in The Gambia, West Africa. J Viral Hepat. 2010;17(2):115-22. doi: 10.1111/j.1365-2893.2009.01168.x

43. Kim GA, Han S, Choi GH, et al. Moderate levels of serum hepatitis B virus DNA are associated with the highest risk of hepatocellular carcinoma in chronic hepatitis B patients. Aliment Pharmacol Ther. 2020;51(11):1169-79. doi: 10.1111/apt.15725

44. World Health Organization. Global Hepatitis Report. Geneva: Licence: CC BY-NC-SA 3.0 IGO.; 2017. https://apps.who.int/iris/bitstream/handle/10665/255016/9789241565455-eng.pdf?sequence=1&isAllowed=y

45. Polaris Observatory, C., Global prevalence, cascade of care, and prophylaxis coverage of hepatitis B in 2022: a modelling study. Lancet Gastroenterol Hepatol, 2023. Doi: 10.1016/S2468-1253(23)00197-8

Figure Legend:

Figure 1 Weighted prevalence estimates of HBV core antibody and HBV surface antigen by 10-year age groups among the study participants, Armenia, 2021.

For peer review only

For peer review only - http://bmjopen.bmj.com/site/about/guidelines.xhtml

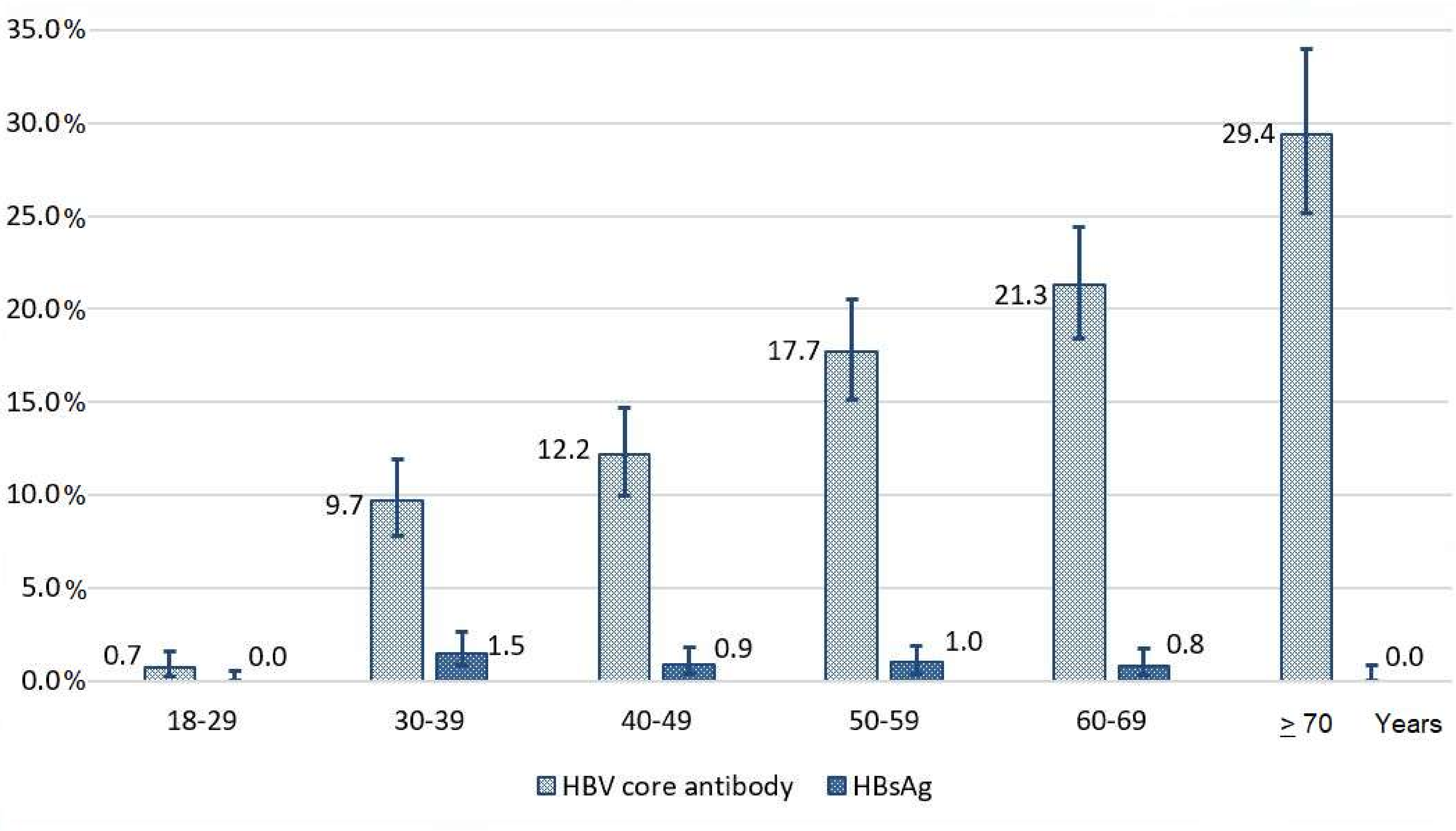
35.0%
30.0%
25.0%
20.0%
15.0%
10.0%
5.0%
0.0%
0.7
0.0
9.7
1.5
12.2
0.9
17.7
1.0
21.3
0.8
29.4
0.0
18-29
30-39
40-49
50-59
60-69
≥ 70
Years
HBV core antibody
HBsAg

Knowledge, Attitude, and Practices Questionnaire on Infectious Diseases, Chronic Hepatitis B and C Virus Infections and Associated Risk Factors

Interviewer

_ _ _ _ _ _ _ _

Participant ID

_ _/_ _/_ _ _/

Blood sampling for Hepatitis

Do not read out loud. Mark as it is mentioned in Excel file.

() Yes → *Show Section 17*

() No → *Skip Section 17*

Date

dd/mm/yy

______ / ______ / ___________

Start of the interview

___:___ (hour:min)

1. Socio-demographic characteristics

The following questions are about your demographic characteristics

1.1 Your nationality

( ) Armenian

( ) Russian

( ) Yazidi

( ) Other, please specify ______________________________

( ) [prefer not to answer]

1.2 Place of residence

( ) City

( ) Village

( ) [prefer not to answer]

For peer review only - http://bmjopen.bmj.com/site/about/guidelines.xhtml

1.3 Your gender

Do not read out loud

( ) Male

( ) Female

1.4 How old are you in the number of years completed? (How old were you on your last birthday)?

dd/mm/yy

_____ / _______ / ____

( ) [prefer not to answer]

1.5 Your highest level of education

Read out all of the options

( ) Incomplete secondary (less than 10 years)
( ) Secondary (10-12 years: high school)
( ) Vocational (12-13 years)
( ) University
( ) Post-graduate
( ) [prefer not to answer]

1.6 How many members are in your family, including yourself?

________________ members

( ) [prefer not to answer]

1.7 Are you currently employed?

Read out all of the options

() Yes, please specify what is your occupation?_________________

() No, I am not employed
() No, I am retired
() No, I am a student
() I work from home/ have my own business
() I am a seasonal worker in foreign countries
() I am a farmer
() Other (specify) ______________________

( ) [prefer not to answer]

For peer review only - http://bmjopen.bmj.com/site/about/guidelines.xhtml

1.8 On average, how much money does your family spend monthly? (Choose one option)

( ) Less than 100,000 AMD
( ) From 101,000 to 200,000 AMD
( ) From 201,000 to 300,000 AMD
( ) From 301,000 to 400,000 AMD
( ) Above 401,000 AMD
( ) Don't know/ Refusal

2. The following questions refer to health and infectious disease literacy.

2.1 How often do you need to have someone help you when you read instructions, pamphlets, or other written material from your doctor or pharmacy? (R*ead the response options, except [prefer not to answer] option)*

1. Never
2. Rarely
3. Sometimes
4. Often
5. Always
6. [prefer not to answer]

On a scale from 1 to 7, how easy or difficult would you say it is to…:

Note to interviewers: Repeat the full question as needed

2.2…find the information you need related to infectious diseases (for example, hepatitis, measles, influenza, COVID-19, HIV, etc.)?

Very difficult [1] [2] [3] [4] [5] [6] [7] Very easy

( ) [prefer not to answer]

2.3…understand information about what to do if you think you have an infectious disease (for example, measles, influenza, COVID-19, HIV, etc.)?

Very difficult [1] [2] [3] [4] [5] [6] [7] Very easy

( ) [prefer not to answer]

2.4…judge if the information about infectious diseases in the media is reliable?

Very difficult [1] [2] [3] [4] [5] [6] [7] Very easy

( ) [prefer not to answer]

For peer review only - http://bmjopen.bmj.com/site/about/guidelines.xhtml

For peer review only

2.5 …understand restrictions and recommendations of authorities regarding infectious diseases?

Very difficult [1] [2] [3] [4] [5] [6] [7] Very easy

( ) [prefer not to answer]

2.6 …follow the recommendations on how to protect yourself from infectious diseases?

Very difficult [1] [2] [3] [4] [5] [6] [7] Very easy

( ) [prefer not to answer]

2.7 …understand recommendations about when to stay at home from work/school, and when not to?

Very difficult [1] [2] [3] [4] [5] [6] [7] Very easy

( ) [prefer not to answer]

2.8 …follow recommendations about when to stay at home from work/school, and when not to?

Very difficult [1] [2] [3] [4] [5] [6] [7] Very easy

( ) [prefer not to answer]

2.9 …understand recommendations about when to engage in social activities, and when not to?

Very difficult [1] [2] [3] [4] [5] [6] [7] Very easy

( ) [prefer not to answer]

2.10…follow recommendations about when to engage in social activities, and when not to?

Very difficult [1] [2] [3] [4] [5] [6] [7] Very easy

( ) [prefer not to answer]

3. The following questions are about infectious disease-related knowledge and behaviors

3.1 What is the most effective measure for preventing measles? *(Do not read, check all that apply)*

1. Vaccination
2. Using medical masks
3. Avoiding contact with those who have measles
4. Other (specify)__________________________
5. Don’t know
6. [prefer not to answer]

For peer review only

3.2 Please indicate whether you agree or disagree with the following statement:

- Antibiotics are effective in treating influenza.
  1. Agree
  2. Disagree
  3. Don't know
  4. [prefer not to answer]

3.3 Please indicate whether you agree or disagree with the following statement:

- Influenza is the same as common cold.
  1. Agree
  2. Disagree
  3. Don't know
  4. [prefer not to answer]

3.4 Please indicate whether you agree or disagree with the following statement:

- Influenza is caused by a virus.
  1. Agree
  2. Disagree
  3. Don't know
  4. [prefer not to answer]

3.5 Do you usually cover your mouth when coughing or sneezing in public places?

1. Yes
2. No
3. [prefer not to answer]

3.6 Do you usually wash your hands before eating?

1. Yes
2. No
3. [prefer not to answer]

4. The following questions are about the symptoms you might have experienced in the past.

4.1 Have you had fever (≥ 37) within the **last six months**?

( ) Yes→ Date (mm/yy) and the highest temperature, (C°):

____

( ) No

( ) Do not remember

( ) [prefer not to answer]

For peer review only - http://bmjopen.bmj.com/site/about/guidelines.xhtml

4.2 Have you had any respiratory symptoms (sore throat, cough, runny nose, shortness of breath, etc.) in the last 3 months?

( ) If yes, specify the date of the last episode → mm/yy: ________________

( ) No → Move to section 12.4

( ) [prefer not to answer] → Move to section 12.4

4.3 Please indicate, if you have had any of the below-mentioned symptoms in the last 6 months?

Respiratory symptoms

	**Yes**	**No**	**[prefer not to answer]**
4.3.a. Sore throat	( )	( )	( )
4.3.b.Cough	( )	( )	( )
4.3.c. Runny nose	( )	( )	( )
4.3.d. Shortness of breath	( )	( )	( )

4.4. Other symptoms

	**Yes**	**No**	**[prefer not to answer]**
4.4.a. Muscle ache	( )	( )	( )
4.4.b. Joint ache	( )	( )	( )
4.4.c. Chills	( )	( )	( )
4.4.d. Rash	( )	( )	( )
4.4.e. Chest pain	( )	( )	( )
4.4.f Vomiting	( )	( )	( )
4.4.g Headache	( )	( )	( )
4.4.h Nausea	( )	( )	( )

For peer review only - http://bmjopen.bmj.com/site/about/guidelines.xhtml

	Yes	No	[prefer not to answer]
4.4.i Loss of taste	( )	( )	( )
4.4.g Loss of smell	( )	( )	( )
4.4.k. Loss of appetite	( )	( )	( )
4.4.l Abdominal pain	( )	( )	( )
4.4.m Diarrhea	( )	( )	( )
4.4.n Conjunctivitis	( )	( )	( )
4.4.o Nose bleed	( )	( )	( )
4.4.p Fatigue	( )	( )	( )
4.4.q Seizures	( )	( )	( )
4.4.r Altered consciousness	( )	( )	( )

For peer review only

4.5.a Other neurological signs

( ) Yes (specify): ____

( ) no

( ) [prefer not to answer]

4.5.b Other symptoms

( ) Yes (specify): ____

( ) No

( ) [prefer not to answer]

For peer review only - http://bmjopen.bmj.com/site/about/guidelines.xhtml

5. The following questions are about accompanying diseases and conditions.

Please indicate if you have had any of the below mentioned diseases/conditions.

5.1. Pregnancy
(Do not ask this question to males)
( ) Yes (specify the trimester: first, second, third, unknown):
____

( ) No
( ) [prefer not to answer]

*Read all of the options, marking **yes** or **no** in front of each. Do not read [prefer not to answer] option.*

	Yes	No	Don't know/Refuse to answer
5.2. Obesity	( )	( )	
5.3. Cancer	( )	( )	
5.4. Diabetes	( )	( )	
5.5. HIV/ other immune deficiency	( )	( )	
5.6. Heart disease	( )	( )	
5.7. Asthma (requiring medication)	( )	( )	
5.8. Chronic lung disease (non-asthma)	( )	( )	
5.9. Chronic liver disease	( )	( )	
5.10. Chronic hematological disorder	( )	( )	
5.11. Chronic kidney disease	( )	( )	
5.12. Chronic neurological impairment/disease	( )	( )	
5.13. Organ or bone marrow recipient	( )	( )	

For peer review only - http://bmjopen.bmj.com/site/about/guidelines.xhtml

5.14. Other accompanying disease(s)

( ) Yes (specify) ____

( ) No

( ) [prefer not to answer]

6. Have you smoked more than 100 cigarettes during your life?

( ) Yes

( ) No

( ) [prefer not to answer]

6.a How would you describe your current smoking status?

Read out all of the options

( ) I smoke everyday – go to question 16.b

( ) I smoke less than everyday

( ) I quit smoking less than a year ago

( ) I quit smoking more than a year ago

( ) [prefer not to answer]

6.b How many cigarettes do you smoke per day? ___________

7. The following questions are about factors that might be associated with Hepatitis C and B infection in the population

7.1 How often do you visit the dentist to have your teeth cleaned, tooth extracted, braces put on, or root canals?

( ) Twice per year

( ) Once a year

( ) Once every 2 years

( ) Once every 3-5 years

( ) It has been more than 5 years

( ) Never

( ) Don’t know/remember/[prefer not to answer]

For peer review only

7.2. Have you received any blood transfusions in your lifetime?

( ) Yes, before 199X
( ) Yes, in or after 199X
( ) No
( ) [prefer not to answer]

7.3 Do you have any tattoos?

( ) Yes
( ) No
( ) [prefer not to answer]

7.4 Have you ever been told by a doctor or other health worker that you had hepatitis B or C? *(multiple answers possible)*

( ) Yes hepatitis B
( ) Yes hepatitis C
( ) Yes, but don't know the type
( ) Neither hepatitis B nor hepatitis C
( ) I am vaccinated against hepatitis B
( ) Don't know/remember/refuse to answer

7.5 Have you ever been in prison (translation: long-term detainment) or jail (translation: short-term detainment)?

( ) Yes
( ) No
( ) [prefer not to answer]

End of interview

___:___ (hour:min)

Notes

____

Thank you!

For peer review only

STROBE Statement—Checklist of items that should be included in reports of ***cross-sectional studies***

	Item No	Recommendation	Page No
**Title and abstract**	1	(*a*) Indicate the study's design with a commonly used term in the title or the abstract	1
		(*b*) Provide in the abstract an informative and balanced summary of what was done and what was found	1
**Introduction**			
Background/rationale	2	Explain the scientific background and rationale for the investigation being reported	3-4
Objectives	3	State specific objectives, including any prespecified hypotheses	5
**Methods**			
Study design	4	Present key elements of study design early in the paper	5-6
Setting	5	Describe the setting, locations, and relevant dates, including periods of recruitment, exposure, follow-up, and data collection	5-6
Participants	6	(*a*) Give the eligibility criteria, and the sources and methods of selection of participants	6
Variables	7	Clearly define all outcomes, exposures, predictors, potential confounders, and effect modifiers. Give diagnostic criteria, if applicable	7-8
Data sources/ measurement	8*	For each variable of interest, give sources of data and details of methods of assessment (measurement). Describe comparability of assessment methods if there is more than one group	*7-8*
Bias	9	Describe any efforts to address potential sources of bias	6-7
Study size	10	Explain how the study size was arrived at	6
Quantitative variables	11	Explain how quantitative variables were handled in the analyses. If applicable, describe which groupings were chosen and why	9
Statistical methods	12	(*a*) Describe all statistical methods, including those used to control for confounding	9-10
		(*b*) Describe any methods used to examine subgroups and interactions	NA
		(*c*) Explain how missing data were addressed	10
		(*d*) If applicable, describe analytical methods taking account of sampling strategy	9
		(*e*) Describe any sensitivity analyses	NA
**Results**			
Participants	13*	(a) Report numbers of individuals at each stage of study—eg numbers potentially eligible, examined for eligibility, confirmed eligible, included in the study, completing follow-up, and analysed	10
		(b) Give reasons for non-participation at each stage	NA
		(c) Consider use of a flow diagram	NA
Descriptive data	14*	(a) Give characteristics of study participants (eg demographic, clinical, social) and information on exposures and potential confounders	11-14
		(b) Indicate number of participants with missing data for each variable of interest	11-14
Outcome data	15*	Report numbers of outcome events or summary measures	10-11

For peer review only - http://bmjopen.bmj.com/site/about/guidelines.xhtml

Main results	16	(*a*) Give unadjusted estimates and, if applicable, confounder-adjusted estimates and their precision (eg, 95% confidence interval). Make clear which confounders were adjusted for and why they were included	10
		(*b*) Report category boundaries when continuous variables were categorized	8
		(*c*) If relevant, consider translating estimates of relative risk into absolute risk for a meaningful time period	NA
Other analyses	17	Report other analyses done—eg analyses of subgroups and interactions, and sensitivity analyses	NA
**Discussion**			
Key results	18	Summarise key results with reference to study objectives	17
Limitations	19	Discuss limitations of the study, taking into account sources of potential bias or imprecision. Discuss both direction and magnitude of any potential bias	20
Interpretation	20	Give a cautious overall interpretation of results considering objectives, limitations, multiplicity of analyses, results from similar studies, and other relevant evidence	20-21
Generalisability	21	Discuss the generalisability (external validity) of the study results	18
**Other information**			
Funding	22	Give the source of funding and the role of the funders for the present study and, if applicable, for the original study on which the present article is based	21

*Give information separately for exposed and unexposed groups.

Note: An Explanation and Elaboration article discusses each checklist item and gives methodological background and published examples of transparent reporting. The STROBE checklist is best used in conjunction with this article (freely available on the Web sites of PLoS Medicine at http://www.plosmedicine.org/, Annals of Internal Medicine at http://www.annals.org/, and Epidemiology at http://www.epidem.com/). Information on the STROBE Initiative is available at www.strobe-statement.org.

For peer review only

For peer review only - http://bmjopen.bmj.com/site/about/guidelines.xhtml

BMJ Open

Prevalence of hepatitis B virus infection among general population of Armenia in 2021 and factors associated with it: a cross-sectional study

Journal:	*BMJ Open*
Manuscript ID	bmjopen-2023-080281.R3
Article Type:	Original research
Date Submitted by the Author:	10-Jan-2024
Complete List of Authors:	Demirchyan, Anahit; American University of Armenia, Turpanjian College of Health Sciences<br>Dudareva, S.; Robert Koch Institut, Department for Infectious Disease Epidemiology<br>Sahakyan, Serine; American University of Armenia, Turpanjian College of Health Sciences<br>Aslanyan, Lusine; American University of Armenia, Turpanjian College of Health Sciences<br>Muradyan, Diana; American University of Armenia, Turpanjian College of Health Sciences<br>Musheghyan, Lusine; American University of Armenia, Turpanjian College of Health Sciences<br>Mozalevskis, Antons; World Health Organization, Global HIV, Hepatitis and Sexually Transmitted Infections Programmes<br>Sargsyants, Narina; National Institute of Health named after academician Suren Avdalbekyan<br>Ghukasyan, Gayane; World Health Organization Country Office in Armenia<br>Petrosyan, V; American University of Armenia, Turpanjian College of Health Sciences
**Primary Subject Heading**:	Epidemiology
Secondary Subject Heading:	Infectious diseases
Keywords:	Epidemiology < INFECTIOUS DISEASES, Infection control < INFECTIOUS DISEASES, Public health < INFECTIOUS DISEASES

SCHOLARONE™
Manuscripts

I, the Submitting Author has the right to grant and does grant on behalf of all authors of the Work (as defined in the below author licence), an exclusive licence and/or a non-exclusive licence for contributions from authors who are: i) UK Crown employees; ii) where BMJ has agreed a CC-BY licence shall apply, and/or iii) in accordance with the terms applicable for US Federal Government officers or employees acting as part of their official duties; on a worldwide, perpetual, irrevocable, royalty-free basis to BMJ Publishing Group Ltd ("BMJ") its licensees and where the relevant Journal is co-owned by BMJ to the co-owners of the Journal, to publish the Work in this journal and any other BMJ products and to exploit all rights, as set out in our licence.

The Submitting Author accepts and understands that any supply made under these terms is made by BMJ to the Submitting Author unless you are acting as an employee on behalf of your employer or a postgraduate student of an affiliated institution which is paying any applicable article publishing charge ("APC") for Open Access articles. Where the Submitting Author wishes to make the Work available on an Open Access basis (and intends to pay the relevant APC), the terms of reuse of such Open Access shall be governed by a Creative Commons licence – details of these licences and which Creative Commons licence will apply to this Work are set out in our licence referred to above.

Other than as permitted in any relevant BMJ Author's Self Archiving Policies, I confirm this Work has not been accepted for publication elsewhere, is not being considered for publication elsewhere and does not duplicate material already published. I confirm all authors consent to publication of this Work and authorise the granting of this licence.

For peer review only

Prevalence of hepatitis B virus infection among general population of Armenia in 2021 and factors associated with it: a cross-sectional study

Demirchyan, Anahit[1*]; Dudareva, Sandra[2]; Sahakyan, Serine[1]; Aslanyan, Lusine[1]; Muradyan, Diana[1]; Musheghyan, Lusine[1]; Mozalevskis, Antons[3]; Sargsyants, Narina[4]; Ghukasyan, Gayane[5]; Petrosyan, Varduhi[1]

[1] Turpanjian College of Health Sciences, American University of Armenia, Yerevan, Armenia

[2] Department for Infectious Disease Epidemiology, Robert Koch Institute, Berlin, Germany

[3] Global HIV, Hepatitis and Sexually Transmitted Infections Programmes, World Health Organization, Geneva, Switzerland

[4] National Institute of Health, Ministry of Health, Republic of Armenia, Yerevan, Armenia

[5] World Health Organization Country Office in Armenia, Yerevan, Armenia

[*] Corresponding author: Demirchyan, Anahit. Address: 40 Marshal Baghramyan ave., Yerevan 0019, Armenia. e-mail: ademirch@aua.am, telephone: +374 60 612562, Fax: +374 60 612512

Keywords: Hepatitis B virus infection; seroprevalence; Armenia; general adult population.

Word count: 4,389

For peer review only

For peer review only - http://bmjopen.bmj.com/site/about/guidelines.xhtml

Abstract

Objectives This study sought to determine the prevalence and associated factors of Hepatitis B virus (HBV) infection ever in life and chronic HBV infection in Armenia.

Design A population-based cross-sectional seroprevalence study combined with a phone survey of tested individuals.

Setting All administrative units of Armenia including ten provinces and capital city Yerevan.

Participants The study frame was the general adult population of Armenia aged ≥18 years.

Primary and secondary outcome measures The participants were tested for anti-HBV core antibodies (anti-HBc) and HBV surface antigen (HBsAg) using third-generation enzyme immunoassays. In case of HBsAg positivity, HBV DNA and hepatitis D virus (HDV) RNA PCR tests were performed. Risk factors of HBV infection ever in life (anti-HBc positivity) and chronic HBV infection (HBsAg positivity) were identified through fitting logistic regression models.

Results The seroprevalence study included 3838 individuals 18 years and older. Of them, 90.7% (3476 individuals) responded to the phone survey. The prevalence of anti-HBc positivity was 14.1% (95% CI 13.1%-15.2%) and HBsAg positivity 0.8% (95% CI 0.5%-1.1%). The viral load was over 10,000 IU/ml for 7.9% of HBsAg-positive individuals. None of the participants was positive for HDV. Risk factors for HBsAg positivity included less than secondary education (aOR=6.44; 95% CI 2.2-19.1), current smoking (aOR=2.56; 95% CI 1.2-5.6), and chronic liver disease (aOR=8.44; 95% CI 3.0-23.7). In addition to these, risk factors for anti-HBc positivity included age (aOR=1.04; 95% CI 1.04-1.05), imprisonment ever in life (aOR=2.53; 95% CI 1.41-4.56), and poor knowledge on infectious diseases (aOR=1.32; 95% CI 1.05-1.67), while living in Yerevan (vs. provinces) was protective (aOR=0.74; 95% CI 0.59-0.93).

Conclusion This study provided robust estimates of HBV markers among general population of Armenia. Its findings delineated the need to revise HBV testing and treatment strategies considering higher risk population groups, and improve population knowledge on HBV prevention.

For peer review only - http://bmjopen.bmj.com/site/about/guidelines.xhtml

STRENGTHS AND LIMITATIONS OF THIS STUDY

- ⇒ Rigorous sampling methodology applied among general adult population of Armenia insured the generalisability of the study findings.
- ⇒ Large sample size allowed to obtain robust prevalence estimates with narrow confidence intervals for HBV infection ever in life and chronic HBV infection.
- ⇒ Items on some important behavioral risk factors including injection drug use and risky sexual behaviors were not included in the questionnaire because of their sensitive nature.
- ⇒ The study methodology did not allow targeting specific population groups at high risk for HBV infection.

For peer review only

For peer review only - http://bmjopen.bmj.com/site/about/guidelines.xhtml

INTRODUCTION

Despite the availability of effective vaccination against hepatitis B virus (HBV), it continues to be a global threat to public health, particularly in low- and middle-income countries (LMIC).(1) According to World Health Organization (WHO) 2019 data, 296 million people in the globe live with chronic hepatitis B, and 1.5 million new infections occur annually.(1) More than a million fatalities globally are attributed to HBV, which is the primary cause of morbidity and mortality from chronic hepatitis, cirrhosis, and hepatocellular carcinoma (HCC), in addition to the acute infection itself.(2, 3)

The commonly known risk factors for HBV are being born to an infected mother, sharing injection syringes and needles, men having sex with men (MSM), female sex workers, living with HIV, and having occupational exposure to blood and blood products.(4-7) Additionally, studies have shown that sociodemographic factors including older age, male sex, low socio-economic status, poor housing, and low education could be risk factors for chronic HBV infection.(8, 9)

There has been a decline in the prevalence of chronic HBV infection in many parts of the world since a reliable vaccine became widely accessible more than 20 years ago.(10) Improved medical practices, such as screening of blood and blood products, applying safe injection techniques, and implementing infection control policies further contributed to this decline.(11)

A noticeable decrease in the incidence of acute viral hepatitis infections was observed in Armenia over the last three decades. The incidence of acute HBV infection steadily decreased from 22.30 in 1990 to 0.17 in 2021 per 100,000 population.(12) One of the explanations of this decrease is the high (over 96%) coverage of hepatitis B newborn and infant vaccination introduced in Armenia in 1999.(13)

Concerning the prevalence of chronic HBV infection, no nationally representative seroprevalence studies of HBV markers were conducted among general population of Armenia

For peer review only

before 2021. Based on synthesis of available screening data on blood donors, pregnant women, healthcare providers, and patients, the best guess was that chronic HBV infection was around 2% among general population of Armenia in 2000.(13) In 2013, based on retrospective testing of frozen blood samples of over 10,000 healthy individuals aged from 1 to 60 years, the median prevalence of HBV surface antigen (HBsAg) was 1.9-2.5%.(14, 15) In the meantime, the prevalence was reported as 1.4% among blood donors, 1.5% among pregnant women, 4.3% among prisoners, 6.6% among hemodialysis patients, 7.3% among healthcare workers, 8.8% among people who inject drugs, and 16.0% among patients with sexually transmitted infections.(15)

A recent review study that explored the groups of population at higher risk for chronic HBV infection in the countries of Caucasus and Central Asia, stated that chronic HBV infection was higher (3.7%) among prisoners in Armenia.(16) Concerning other vulnerable groups, the estimates varied across different small-scale studies from 0% to 1.6% for MSM and from 0.3% to 0.9% for migrants (mainly seasonal workers returning from Russia).(16)

For the purpose of assessing national disease prevention and control initiatives including immunization programs, knowledge of the region- and age-specific prevalence of hepatitis B infection is essential. The study presented in this paper was the first attempt to identify the seroprevalence of HBV infection among general adult population of Armenia. The study aimed to determine the prevalence of HBV infection ever in life and chronic HBV infection in Armenia and identify the risk factors of each of these outcomes among the general adult population.

METHODS

Study design

This study was a cross-sectional population-based sero-epidemiological study conducted throughout Armenia in May-September, 2021. The study included laboratory testing of blood

For peer review only - http://bmjopen.bmj.com/site/about/guidelines.xhtml

serum samples of the participants for biomarkers of hepatitis B, hepatitis C, and SARS-CoV-2 viruses, which was shortly followed with a telephone survey among them. The study was primarily designed to measure the biomarkers for SARS-CoV-2 and the hepatitis markers were added to it. The current paper reflects the analytical approaches and findings on HBV infection.

Sampling and sample size

The study frame was the general adult population of Armenia aged 18 years and over. We used a proportionate-to-the-population-size cluster sampling design, which included all 11 administrative units of Armenia (the ten provinces and Yerevan city). The study participants were recruited from primary healthcare (PHC) facilities, as the coverage of population with PHC is quite high in Armenia (about 97%). We selected one-third of the polyclinics in each administrative unit using random sampling – proportionate to the served population size. Simple random sampling was then applied in the national e-health operator "Armed" database to select the study participants from the lists of 18 years old and older individuals served by each chosen polyclinic.

The formula for estimating a proportion ($n = N*X / (X + N – 1)$, where $X = Z_{\alpha/2}^2 *p*(1-p) / (MOE)^2$) of the population was used to calculate the sample size for this study. When assuming 30% prevalence of anti-SARS-CoV-2 antibodies, 1.45% margin of error (MOE), type 1 error of 0.05, and confidence level of 95%, the calculated sample size was 3832. Assuming expected HBsAg prevalence of 2% and HBV core antibody (Anti-HBc) prevalence of 15%, this sample size allowed calculation of HBsAg prevalence estimate with a MOE of 0.44% and Anti-HBc prevalence estimate with a MOE of 1.13%.

Data collection

The "Armed" e-health system provided the list of selected unique ID numbers to the respective polyclinic's personnel trained for contacting the study participants. The personnel contacted each

For peer review only - http://bmjopen.bmj.com/site/about/guidelines.xhtml

potential participant by phone, following the script for the first contact, introduced the study and invited her/him to the polyclinic to take part in the study. When the participants came to the PHC facility, the research team obtained a written informed consent and collected blood samples. The participant recruitment in each polyclinic continued until the needed sample size was reached. Blood sampling was done by a trained nurse. Telephone interviews were carried out by trained interviewers within two weeks after the blood sampling. The structured questionnaire was administered using electronic tablets where Alchemer online tool was installed.

The study protocol was reviewed and approved by the Ethics Review Committee of the World Health Organization (protocol ID: CERC.0013C) and the Institutional Review Board of the American University of Armenia (protocol ID: AUA-2021-005), as conforming to the principles embodied in the Declaration of Helsinki. If the test result was positive, a trained hepatologist communicated the result to the individual and provided counseling. Negative results were sent to the participants via SMS messages.

Serum tests and definitions

Via venipuncture, 5-10 ml venous blood was collected from each participant and centrifuged immediately for the serum extraction. The serums were kept at 2-8°C for seven days at most before being tested at the laboratory of the National Center for Infectious Diseases of the Republic of Armenia. This laboratory was chosen because of having the needed technical and human resources to perform third generation serological and molecular testing with the required quality for all three infections included in the study. The testing procedure for HBV included the following steps: (1) all samples were tested for total anti-HBc antibodies using third-generation enzyme immunoassay (Elecsys® Anti-HBc II-Roche Diagnostics (17)); (2) if anti-HBc antibodies were detected, HBsAg test was performed (using Elecsys HBsAg-Roche Diagnostics (18)), followed by a confirmatory test if the result was questionable (within 0.9-1.0 COI); (3) if HBsAg test was positive, HBV DNA test was performed to test for the presence of HBV DNA

For peer review only - http://bmjopen.bmj.com/site/about/guidelines.xhtml

and the viral load (using Amplisens HBV Monitor FL (detection limit 150 IU/ml)); (4) all samples positive for HBsAg were also tested for hepatitis D virus (HDV) RNA (PCR test using Amplisens HDV-FL (detection limit 100 copies/ml)). All the applied tests were WHO pre-qualified. The presence of antibodies against HBV core antigen (anti-HBc antibodies) was viewed as serological evidence of HBV infection ever in life, while the presence of HBV surface antigen (HBsAg[1]) served as serological evidence of chronic HBV infection.

Survey instrument and study variables

The two outcome variables of this study were anti-HBc antibody status and HBsAg status measuring HBV infection ever in life and chronic HBV infection, respectively. The survey tool was a structured questionnaire which included items related to this study grouped in socioeconomic, health status, health literacy, and health behavioral domains. The sociodemographic domain included three continuous (age, family size, and socioeconomic status (SES) score) and four categorical (sex, education, employment, and residence) variables. The health status domain included only categorical (dichotomous) variables: hepatitis C exposure and chronic infection, several other self-reported chronic conditions (diabetes, chronic liver disease, obesity, heart disease, cancer, chronic kidney disease, and asthma requiring medication), as well as symptoms experienced during the six months prior to the survey (vomiting, fatigue, loss of appetite, joint ache, abdominal pain, fever, and nose bleeding). The health literacy domain included a communicable diseases knowledge variable measured by four questions and dichotomized at its lower third, and a score measuring the difficulty of understanding health-related information generated from responses to ten items. The health behavioral domain included the following categorical variables: ever and current regular smoking, having tattoos, ever having been imprisoned, ever receiving blood transfusion, frequency of visiting a dentist,

[1] The WHO surveillance case definition for chronic HBV infection assumes the presence of biomarkers of the infection combined with the absence of signs and symptoms of acute hepatitis. We applied this definition as coming across a recent infection in a cross-sectional population study is unlikely.

For peer review only - http://bmjopen.bmj.com/site/about/guidelines.xhtml

and self-reported hepatitis B vaccination status. A variable on ever having been told by a doctor of being infected with HBV was also included in the analysis. The questionnaire is provided as a supplementary file.

Patient and public involvement statement

The survey questionnaire was pretested among eligible people and refined based on their feedback. This was the only involvement of public in this research.

Data analysis

We used IBM SPSS Statistics for Windows, version 21 (IBM Corp., Armonk, N.Y., USA) for data management and analysis. All the analysis was weighted by sex and 5-year age groups to closely mimic age and sex distribution of the population of Armenia, as age and sex distribution in the study sample differed from that in the general population due to differences in response rates of different age and sex groups. To estimate the prevalence of HBV infection ever in life and chronic HBV infection, proportions and 95% confidence intervals were calculated for positivity to anti-HBc and HBsAg, respectively. To descriptively analyze independent variables, frequencies and proportions were calculated for categorical variables, and means and standard deviations for continuous variables in the total sample and in each of the outcome groups differentiated based on positivity/negativity to anti-HBc and positivity/negativity to HBsAg. Then univariate logistic regression analysis was done. The linearity of the association of continuous variables with each outcome variable was checked on the logit scale before entering them into logistic regression analysis.(19) The final step was fitting logistic regression models to identify factors independently associated to each of the outcomes. A series of multivariable logistic regression analyses were performed with inclusion of all those variables associated with the outcome at the $p < 0.25$ level in the univariate analysis.(19) The variables were entered into logistic regression analysis in different combinations using e*nter* method. The variables not

For peer review only - http://bmjopen.bmj.com/site/about/guidelines.xhtml

related to the outcome ($p \geq 0.10$) when controlling for the remaining variables in the model were excluded from the model, if their removal did not change the associations between the remaining variables and the outcome. The decision on keeping variables with a p-value between 0.05 and 0.10 in the model was made based on their potential relevance for interpretation of the data. The final models were checked for model fit and collinearity. Cases with any missing values were excluded from the fitted models.

RESULTS

Trained personnel from the selected polyclinics contacted a total of 9009 eligible individuals throughout the country to invite them for blood testing. Of them, 3831 (42.5%) individuals agreed and underwent blood testing. Of those tested, 3476 individuals (90.7%) participated in the phone survey. The prevalence numbers of HBV markers were generated from the sample that was tested (n=3831), but the variables needed for the rest of the analysis (descriptive comparisons and logistic regression) were available only for those 3476 individuals who participated in the phone survey and answered the survey questionnaire. Of them, 54.5% were women and 45.5% men. The mean age of women was 46.8 years (95% CI 46.0; 47.6), and the mean age of men 44.5 years (95% CI 43.6; 45.3). Of men, 43.9%, and of women, 41.0% had university or higher education, while 6.3% of men and 3.0% of women had lower than secondary education. Over one-third of the participants (36.3%) were from Yerevan and 63.7% from the provinces of Armenia. Further descriptive findings and comparisons between the groups with and without HBV markers are provided in Tables 1 and 2 and discussed later in the Results.

Prevalence of past and present HBV infection

The weighted percentage of those positive for anti-HBc antibodies indicating past or present HBV infection was 14.1% (95% CI 13.1%-15.2%). This percentage significantly increased with age (Figure 1) and was slightly higher among men than women (15.0% versus 12.2%, p=0.017).

For peer review only - http://bmjopen.bmj.com/site/about/guidelines.xhtml

The weighted percentage of those carrying HBsAg – the prevalence of chronic HBV infection – was 0.8% (95% CI 0.5%-1.1%). This estimate was the highest in the age group of 30-39 years old (1.5%, 95% CI 0.8%-2.6%) (Figure 1) and was higher among men compared to women (1.1% versus 0.5%, p=0.048). The measurement of the viral load among those positive for HBsAg showed that for 61.9% of them, HBV DNA was below 150 IU/ml, for 30.2% it was between 150 IU/ml and 2,000 IU/ml, and for 7.9% (n=2) – over 2,000 IU/ml (in the weighted analysis). For both these cases, the viral load was 11,000 IU/ml. For those HBV DNA-positive individuals with measurable (≥150 IU/ml) viral load, the median value of HBV DNA was 750 IU/ml and the interquartile range – 3869.6 IU/ml. None of the HBsAg-positive samples were positive for HDV RNA.

Results of descriptive comparisons

Table 1 demonstrates the results of weighted crude comparisons of the study variables between the groups infected with HBV ever in life and those never infected with HBV.

Table 1 Distribution of selected variables by participants' anti-HBV core antibody status, Armenia, 2021

			Anti-HBV core antibody			
		N*	Positive (n=469)	Negative (n=3005)	*p-value*	Total (n=3474)
Socioeconomic variables:						
Age, mean (SD)		3473	57.1 (15.7)	44.0 (16.9)	*<0.001*	45.8 (17.3)
Socio-economic status score, mean (SD)		2696	46.8 (34.8)	50.3 (35.3)	*0.075*	49.9 (35.2)
Family size, mean (SD)		3451	4.2 (2.1)	4.4 (1.7)	*0.004*	4.4 (1.8)
Sex:	Female, %	3473	49.4	55.3	*0.017*	54.5
	Male, %		50.6	44.7		45.5
Education:	Below secondary, %	3456	7.7	4.0	*<0.001*	4.5
	Secondary/vocational, %		61.5	52.0		53.3
	University/higher, %		30.8	44.0		42.3
Employment:	Employed/student, %	3438	41.8	58.6	*<0.001*	56.4
	Unemployed/seasonal migrant, %		27.2	28.9		28.7
	Retired/disabled, %		30.9	12.5		15.0
Residence:	Yerevan city, %		28.6	37.4	*<0.001*	36.3
	Provinces, %		71.4	62.6		63.7
Health status variables:						
Anti-Hepatitis C antibodies positive, %		3474	3.6	1.7	*0.011*	2.0

For peer review only - http://bmjopen.bmj.com/site/about/guidelines.xhtml

	N*	Anti-HBV core antibody Positive (n=469)	Negative (n=3005)	*p-value*	Total (n=3474)
Hepatitis C PCR test positive, %	3474	1.5	0.6	*0.034*	0.7
Diabetes, %	3395	12.4	7.1	*<0.001*	7.8
Chronic liver disease, %	3391	8.4	2.1	*<0.001*	2.9
Obesity, %	3383	13.8	9.5	*0.007*	10.1
Heart disease, %	3390	29.0	12.6	*<0.001*	14.8
Cancer, %	3394	2.0	1.5	*0.408*	1.5
Chronic kidney disease, %	3395	4.6	3.6	*0.286*	3.7
Asthma requiring medication, %	3391	2.0	2.3	*0.864*	2.2
Symptoms experienced during the last six months:					
Vomiting, %	3387	3.3	4.6	*0.268*	4.5
Fatigue, %	3391	41.8	46.0	*0.104*	45.4
Loss of appetite, %	3388	8.9	14.4	*0.001*	13.6
Joint ache, %	3393	37.6	33.4	*0.078*	33.9
Abdominal pain, %	3382	9.4	11.2	*0.257*	11.0
Fever, %	3395	16.5	25.4	*<0.001*	24.2
Nose bleed, %	3387	2.9	4.6	*0.108*	4.4
Health literacy variables:					
Poor knowledge on infectious diseases, %	3474	32.8	24.3	*<0.001*	25.5
Health information understanding score, mean (SD)	3005	52.2 (11.5)	54.4 (9.9)	*<0.001*	54.1 (10.1)
Health behavioral variables:					
Current smoking, %	3389	25.7	20.7	*0.016*	21.4
Ever smoking, %	3395	39.5	34.0	*0.026*	34.7
Having tattoos, %	3380	13.0	10.0	*0.055*	10.4
Undergoing blood transfusion, %	3380	4.0	3.4	*0.487*	3.5
Ever having been imprisoned, %	3387	4.9	1.2	*<0.001*	1.7
Visiting a dentist: Once in 3-5 years/more, %	3329	52.5	74.3	*<0.001*	71.4
Less frequently/never, %		47.5	25.7		28.6
Vaccinated against Hepatitis B (self-reported), %	3397	0.2	0.2	*1.000*	0.2
Hepatitis B told by doctor, %	3397	2.7	0.2	*<0.001*	0.5

N, Number of valid responses; HBV, Hepatitis B virus; PCR, Polymerase chain reaction; SD, Standard deviation.
* Weighted estimates; *p-value:* two-sided.

For peer review only

The ever-infected group was older, with lower level of education, from a smaller-size family, and more often – residing in provinces. The proportion of employed was lower among ever infected group and the proportion of retired/disabled was higher. Anti-hepatitis C antibodies were found among the ever-infected group more frequently. The ever-infected group more frequently reported some chronic health conditions – diabetes, chronic liver disease, obesity, and heart disease. However, some symptoms experienced during the last six months, including loss

For peer review only - http://bmjopen.bmj.com/site/about/guidelines.xhtml

of appetite and abdominal pain, were less frequently reported by the ever-infected group. The ever-infected group demonstrated poor knowledge on infectious diseases. The mean score of understanding health-related information was also lower among them compared to the never infected group. There were significant differences between the groups in the health behavioral domain as well. Both current and ever smokers were more prevalent among the ever infected. Higher proportion of them were ever imprisoned, and lower proportion used dental services at least once in 3-5 years. A similar negligible proportion of respondents (0.2%) in both groups reported having been vaccinated against hepatitis B.

The characteristics of those participants with and without chronic HBV infection were compared in Table 2.

Table 2 Distribution of selected variables by Hepatitis B surface antigen (HBsAg) status, Armenia, 2021

			HBsAg			
		N*	Positive (n=27)	Negative (n=3447)	*p-value*	Total (n=3474)
Socioeconomic variables:						
Age, mean (SD)		3473	44.7 (11.9)	45.8 (17.4)	*0.734*	45.8 (17.3)
Socio-economic status score, mean (SD)		2696	43.7 (24.3)	49.9 (35.3)	*0.471*	49.9 (35.2)
Family size, mean (SD)		3451	4.2 (1.3)	3.4 (1.8)	*0.595*	4.4 (1.8)
Sex:	Female, %	3473	40.7	54.7	*0.048*	54.5
	Male, %		65.4	45.3		45.5
Education:	Below secondary, %	3455	22.2	4.3	*<0.001*	4.5
	Secondary/vocational, %		44.4	53.3		53.3
	University/higher, %		33.3	42.3		42.3
Employment:	Employed/student, %	3439	48.1	56.4	*0.607*	56.4
	Unemployed/seasonal migrant, %		37.0	28.6		28.7
	Retired/disabled, %		14.8	15.0		15.0
Residence:	Yerevan city, %		29.6	36.3	*0.551*	36.3
	Provinces, %		70.4	63.7		63.7
Health status variables:						
Anti-Hepatitis C antibodies positive, %		3474	7.4	1.9	*0.097*	2.0
Hepatitis C PCR test positive, %		3474	3.7	0.7	*0.171*	0.7
Diabetes, %		3396	11.1	7.8	*0.464*	7.8
Chronic liver disease, %		3390	19.2	2.8	*0.001*	2.9
Obesity, %		3384	14.8	10.0	*0.343*	10.1
Heart disease, %		3389	14.8	14.8	*1.000*	14.8
Cancer, %		3395	0.0	1.5	*1.000*	1.5
Chronic kidney disease, %		3395	3.7	3.7	*1.000*	3.7
Asthma requiring medication, %		3391	3.7	2.2	*0.459*	2.2
Symptoms experienced during the last six months:						

For peer review only - http://bmjopen.bmj.com/site/about/guidelines.xhtml

	N*	HBsAg Positive (n=27)	HBsAg Negative (n=3447)	*p-value*	Total (n=3474)
Vomiting, %	3388	0.0	4.5	*0.632*	4.5
Fatigue, %	3392	40.7	45.5	*0.700*	45.4
Loss of appetite, %	3388	0.0	13.7	*0.042*	13.6
Joint ache, %	3394	48.1	33.8	*0.151*	33.9
Abdominal pain, %	3382	4.2	11.0	*0.508*	11.0
Fever, %	3395	11.1	24.3	*0.173*	24.2
Nose bleed, %	3387	0.0	4.4	*0.630*	4.4
Health literacy variables:					
Poor knowledge on infectious diseases, %	3474	37.0	25.4	*0.183*	25.5
Health information understanding score, mean (SD)	3005	48.7 (14.4)	54.2 (10.1)	*0.016*	54.1 (10.1)
Health behavioral variables:					
Current smoking, %	3390	44.4	21.2	*0.007*	21.4
Ever smoking, %	3394	53.8	34.6	*0.060*	34.7
Having tattoos, %	3379	19.2	10.3	*0.182*	10.4
Undergoing blood transfusion, %	3380	3.7	3.5	*0.615*	3.5
Ever having been imprisoned, %	3387	11.6	1.6	*0.010*	1.7
Visiting a dentist: Once in 3-5 years/more, %	3330	74.1	71.4	*0.834*	71.4
Less frequently/never, %		25.9	28.6		28.6
Vaccinated against Hepatitis B (self-reported), %	3398	0.0	0.2	*1.000*	0.2
Hepatitis B told by doctor, %	3398	22.2	0.4	*<0.001*	0.5

N, Number of valid responses; PCR, Polymerase chain reaction; SD, Standard deviation.
* Weighted estimates; *p-value:* two-sided.

Those chronically infected had lower education and higher composition of male. They reported chronic liver disease much more frequently. The proportion of those demonstrating poor knowledge on infectious diseases was higher among them and the mean score of understanding health-related information was lower. Smoking was more frequent among them, and they have been imprisoned much more frequently than those free from the virus.

Among those testing positive for anti-HBc, 12 (2.7%) reported that they have ever been told by a doctor that they were infected with hepatitis B. Six of these 12 persons were also positive for HBsAg, thus only 22.2% of those HBsAg positive were aware of their status (Tables 1, 2).

Findings of regression analysis

The fitted logistic regression model of factors independently associated with ever being infected with HBV included the following characteristics – age, education, Yerevan vs. province

For peer review only - http://bmjopen.bmj.com/site/about/guidelines.xhtml

residence, chronic liver disease, current smoking, imprisonment ever in life, and knowledge on infectious diseases (Table 3).

Table 3 Multivariable logistic regression model of determinants of HBV infection ever in life (positivity for anti-HBV core antibody) among adult population in Armenia, 2021 (valid N=3349)

Characteristics		aOR	95% CI	*p- value*
Age, years		1.04	1.04 - 1.05	< 0.001
Education:	Less than secondary	1.61	1.02 - 2.56	0.042
	Secondary/vocational	1.26	1.00 - 1.59	0.052
	University/higher	1.00	ref.	ref.
Living in Yerevan vs. provinces		0.74	0.59 - 0.93	0.011
Chronic liver disease		2.93	1.88 - 4.56	<0.001
Current smoking		1.60	1.25 - 2.06	<0.001
Ever imprisoned		2.53	1.41 - 4.56	0.002
Poor knowledge on infectious diseases		1.32	1.05 - 1.67	0.018

HBV, hepatitis B virus.
N, number of cases.
aOR, adjusted odds ratio.
CI, confidence interval.

When holding all the other variables constant, each year increase in age increased the likelihood of ever being infected with HBV by 4%. Compared to having university/higher education, having less than secondary education was associated with 1.61 times higher odds, and having secondary/vocational education with 1.26 times higher odds of ever being infected with HBV. Residing in Yerevan was protective – 0.74 times decreasing the odds of HBV infection ever in life. Suffering from chronic liver disease was associated with 2.93 times higher odds of HBV infection ever in life. Ever being imprisoned increased the odds of HBV infection ever in life 2.53 times. Current smoking was also a risk factor for ever having HBV infection, increasing its odds 1.60 times. Poor knowledge of infectious diseases was associated with 1.32 times higher odds of having HBV infection ever in life. The model had no collinearity issues with all Variance Inflation Factor (VIF) values being below 1.13.

For peer review only - http://bmjopen.bmj.com/site/about/guidelines.xhtml

Several factors associated with having HBV infection ever in life, including education, chronic liver disease, and current smoking, were also significant in the fitted model of determinants of chronic HBV infection (Table 4).

Table 4 Multivariable logistic regression model of determinants of chronic HBV infection (HBsAg positivity) among adult population of Armenia, 2021 (valid N=3349)

Characteristics		aOR	95% CI	*p- value*
Education:	Less than secondary	6.44	2.17 - 19.12	<0.001
	Secondary/vocational	1.01	0.42 - 2.45	0.984
	University/higher	1.00	ref.	ref.
Chronic liver disease		8.44	3.00 - 23.72	<0.001
Current smoking		2.56	1.16 - 5.65	0.019
Ever imprisoned		3.33	0.81 - 13.74	0.096

HBV, hepatitis B virus.
HBsAg, Hepatitis B surface antigen.
N, number of cases.
aOR, adjusted odds ratio.
CI, confidence interval.

Compared to secondary or higher education, less than secondary education increased the odds of having chronic HBV infection 6.4 times. Chronic liver disease was associated with 8.4 times higher odds of having chronic HBV infection. Current smoking increased the likelihood of chronic HBV infection 2.6 times. Ever having been imprisoned was also potentially associated with having chronic HBV infection, as the observed effect range between these variables was suggestive of a weak negative to a strong positive association (aOR 3.33; 95% CI 0.81 - 13.74). Again, no collinearity issues were identified in this model with all VIF values being below 1.06.

DISCUSSION

This is the first population-based seroprevalence study to estimate the nationwide prevalence of HBV infection in Armenia. The study demonstrated that the proportion of adult population ever infected with hepatitis B was considerably high in Armenia (14.1%; 95% CI 0.5%,1.1%). Yet, the prevalence of chronic HBV infection was low (0.8%; 95% CI 13.1%, 15.2%), and none of

For peer review only - http://bmjopen.bmj.com/site/about/guidelines.xhtml

the HBsAg-positive participants were positive for HDV RNA. The identified prevalence in Armenia was lower than in a study carried out in the neighboring Georgia in 2021: the prevalence of chronic hepatitis B among adults in Georgia was 2.7% (95% CI 2.3%, 3.4%) and the proportion of persons ever infected with hepatitis B was 21.7% (95% CI 20.4%, 23.2%).(20). Previous data for Armenia based on modeling studies showed a higher prevalence of chronic HBV infection. According to a modeling study from 2016, the HBsAg prevalence in Armenia was estimated at 1.9%,(21) and based on the Coalition for Global Hepatitis Elimination estimates, it was gradually decreasing from 3.4% in 1994/95.(22) Surveillance data confirm that there has been a continuous reduction of new cases – mainly explained by introduction of hepatitis B vaccination among children in 1999.(13, 15) This will likely contribute to further decrease in prevalence of chronic HBV infection in Armenia.(23) The prevalence in 2016 was probably an overestimation as it was relying on proxy data and not a measurement attributable to general population in Armenia. Data from screening of pregnant women in Armenia support the current finding of low prevalence. For the years 2017-2021, from over 97,000 pregnant women that underwent screening, 0.66% were HBsAg positive.(24)

In crude weighted comparisons, the prevalence of both HBsAg and anti-HBc were higher in males than in females, which is similar to other studies.(20, 25, 26) Generally, difference in the prevalence between men and women may be related to either behavioral factors like higher sexual risk behaviors, intravenous drug use and imprisonment in men, or to biological factors.(27) Based on studies, it has been discussed that females usually develop more intense innate, humoral and cellular responses to viral infections, including hepatitis virus, than males.(27, 28) After controlling for other variables, this association, however, disappeared in the model.

While for ever being infected with HBV, it was possible to detect a statistically significant increase in the prevalence with increasing age, there were no detectable differences with

For peer review only - http://bmjopen.bmj.com/site/about/guidelines.xhtml

increasing age in the prevalence of chronic hepatitis B. However, the analysis of HBsAg prevalence by different groups was limited by low number of positive cases and, correspondingly, lack of power with the given sample size. Descriptive results, however, show that there were no chronic hepatitis B cases among those 18-29 years of age. Individuals up to age of 22 have been born after the introduction of the birth dose and childhood hepatitis B vaccination in Armenia. This potentially leads to less cases in this age group. According to the reports submitted to WHO, the impact of hepatitis B vaccination on the control of mother to child transmission has been demonstrated in several countries of the WHO European Region with historically intermediate and high hepatitis B endemicity, like Republic of Moldova, Georgia, Kyrgyzstan, Uzbekistan, Tajikistan and Turkmenistan.(23, 29) We also did not find any HBsAg positive cases among those 70 years and older, while the percentage positive for anti-HBc was the highest in this group. On one hand, those with persistent chronic infection might have already had a lethal outcome at that age, on other hand, the sample size in this age group (n=274) might have been a limiting factor to detect prevalent cases. Furthermore, the HBsAg clearance rate with and without therapy might also influence this figure.(30) Several other factors associated with higher odds for current or past infection were identified in the multivariable regression models. For both ever being infected and chronic infection, odds were higher among people with less than secondary education and current smokers. Odds for ever being infected were higher among persons living in provinces (versus Yerevan), those demonstrating poorer knowledge on infectious diseases, and those ever imprisoned. Individuals with lower levels of education, smoking, and individuals who have been imprisoned might be more likely to engage in riskier behavior such as drug use and unprotected sexual activity leading to increased exposure to HBV.(31-33) Furthermore, lower education levels and socioeconomic disadvantages can result in reduced access to healthcare services and prevention.(34, 35) Higher odds for ever being infected with HBV in provinces compared to Yerevan could be as well related to historically

For peer review only - http://bmjopen.bmj.com/site/about/guidelines.xhtml

poorer access to health care or lower level of infection prevention and control measures in regional heathcare centers. Though, there is no documented evidence supporting this assumption. We could also demonstrate that poorer knowledge about infectious diseases was associated with higher odds for detection of anti-HBc, possibly explained by poorer knowledge about transmission routes, limited understanding of preventive measures, misconceptions and stigma, and poorer access to health related information.(33, 36, 37)

In 7.9% of chronic infections (n=2 in the weighted analysis), the viral load was above 10,000 IU/ml, indicating the need for treatment.(38) A viral load above 10,000 IU/ml is a strong risk predictor of HCC, independent of HBeAg status, ALT level and liver cirrhosis.(39-43) The results on viral load suggest that there is a tangible proportion of chronic hepatitis B patients in Armenia that are currently not receiving antiviral treatment or were not aware of their infection. As the testing and treatment costs in case of a chronic HBV infection are currently mainly related to out-of-pocket payments in Armenia, the detection of cases and sustaining them in care is hindered.(24).

Based on our prevalence findings (0.8%) and given the adult population size of Armenia (~2,500,000), the estimated number of people living with chronic HBV infection in Armenia could be around 20,000, and 1,600 of them (7.9% with a viral load above 10,000 IU/ml) could be at high risk of HCC and in need for immediate treatment. Yet, roughly, only one-fifth of chronically infected are aware of their infection. The WHO target for 2030 is 90% of people living with hepatitis B diagnosed, which calls for improved access to testing to reach the global and regional goals.(44) Hence, there is a need to revise testing and treatment strategies in Armenia in order to find and timely treat chronic hepatitis B cases.

Limitations: Due to limited length of the questionnaire we were not able to include all relevant questions to determine other factors associated with higher odds of being infected with HBV. Also, as the study was conducted during the COVID-19 pandemic, some of the reported

For peer review only - http://bmjopen.bmj.com/site/about/guidelines.xhtml

symptoms experienced within six months prior to the survey could be related to COVID disease rather than participant's general health status. Furthermore, as the sample size was calculated for the SARS-CoV-2 study needs, our possibilities for calculation of the prevalence of HBV infection for different groups were limited and we might lack power to detect differences among groups and when performing regression analyses. At the same time, the sample size was appropriate to measure nationwide robust estimates with narrow confidence intervals.

RECOMMENDATIONS

In order to diagnose persons living with HBV, testing should be scaled up, especially among the population groups that are likely more affected. Improvement of state-funded preventive and testing strategies and warranting the access to state-funded hepatitis B treatment is needed in order to find and timely treat cases to prevent sequel like cirrhosis and HCC, and decrease mortality. Furthermore, to reach elimination of hepatitis B, prevention of mother-to-child transmission should be maintained at high level.(45) Validation of reaching hepatitis B control targets with a study targeting younger children (from the vaccinated cohort) could be considered in Armenia.

Also, population groups at high risk for HBV infection due to behavioral or socio-economic reasons should be targeted with preventive measures including information campaigns, vaccination and access to needle-syringe programs. Actions to improve knowledge on hepatitis B transmission routes, prevention and testing are needed for all population groups.

CONCLUSIONS

This is the first nation-wide representative study in Armenia providing robust estimates of anti-HBc and HBsAg prevalence among general population. While the prevalence of chronic hepatitis B is rather low in Armenia, the proportion of persons ever been infected with HBV is

For peer review only - http://bmjopen.bmj.com/site/about/guidelines.xhtml

considerable. Age distribution of HBV infection ever in life suggests that there has been historically high incidence and that new infections possibly still occur among adults. Meanwhile, the undetectable prevalence among younger age groups suggests the positive impact of the hepatitis B vaccination.

Acknowledgements This work was done with partial support from WHO Regional Office for Europe and technical assistance from Robert Koch Institute. The authors alone are responsible for the views expressed in this article and they do not necessarily represent the views, decisions, or policies of the institutions with which they are affiliated.

"This study/research was partially funded by the World Health Organization (WHO). The results of the research and the underlying data belong the Ministry of Health of the Republic of Armenia. The authors have been given permission to publish this article.

The authors alone are responsible for the views expressed in this article and they do not necessarily represent the views, decisions or policies of the institutions with which they are affiliated".

Contributors AD, SS, LM, AM, NS, GG and VP conceptualized this study, AD performed the analysis. AD, SD, LA, and DM drafted the manuscript. All authors supported the interpretation of the study results and critically reviewed and revised the manuscript.

Competing interests None declared.

Funding The data collection was partially funded by WHO Regional Office for Europe (no grant number). No funding was received for developing this manuscript.

Data availability statement Data are available on reasonable request.

For peer review only - http://bmjopen.bmj.com/site/about/guidelines.xhtml

Ethics Approval Statement The study protocol was reviewed and approved by the Ethics Review Committee of the World Health Organization (protocol ID: CERC.0013C) and the Institutional Review Board of the American University of Armenia (protocol ID: AUA-2021-005), as conforming to the principles embodied in the Declaration of Helsinki.

For peer review only

For peer review only - http://bmjopen.bmj.com/site/about/guidelines.xhtml

REFERENCES

1. G. B. D. Hepatitis B Collaborators. Global, regional, and national burden of hepatitis B, 1990-2019: a systematic analysis for the Global Burden of Disease Study 2019. Lancet Gastroenterol Hepatol. 2022;7(9):796-829. doi: 10.1016/S2468-1253(22)00124-8

2. Purcell RH. The discovery of the hepatitis viruses. Gastroenterology. 1993;104(4):955-63. doi: 10.1016/0016-5085(93)90261-a

3. Tseng TC, Liu CJ, Yang HC, et al. High levels of hepatitis B surface antigen increase risk of hepatocellular carcinoma in patients with low HBV load. Gastroenterology. 2012;142(5):1140-9 e3; quiz e13-4. doi: 10.1053/j.gastro.2012.02.007

4. Zhao X, Shi X, Lv M, et al. Prevalence and factors associated with hepatitis B virus infection among household members: a cross-sectional study in Beijing. Hum Vaccin Immunother. 2021;17(6):1818-24. doi: 10.1080/21645515.2020.1847951

5. Karoney MJ, Some FF, Dip HIL, et al. Burden of Hepatitis B Infection among High Risk Populations in Western Kenya. J Infect Dis Epidemiol 2020;6:132. doi: 10.23937/2474-3658/1510132

6. Muljono DH, Wijayadi T, Sjahril R. Hepatitis B Virus Infection among Health Care Workers in Indonesia. Euroasian J Hepatogastroenterol. 2018;8(1):88-92. doi: 10.5005/jp-journals-10018-1269

7. Hou J, Liu Z, Gu F. Epidemiology and Prevention of Hepatitis B Virus Infection. Int J Med Sci. 2005;2(1):50-7. doi: 10.7150/ijms.2.50

8. Gnyawali B, Pusateri A, Nickerson A, et al. Epidemiologic and socioeconomic factors impacting hepatitis B virus and related hepatocellular carcinoma. World J Gastroenterol. 2022;28(29):3793-802. doi: 10.3748/wjg.v28.i29.37934.

For peer review only - http://bmjopen.bmj.com/site/about/guidelines.xhtml

9. Tosun S, Aygun O, Ozdemir HO, et al. The impact of economic and social factors on the prevalence of hepatitis B in Turkey. BMC Public Health. 2018;18(1):649. doi: 10.1186/s12889-018-5575-6

10. Pattyn J, Hendrickx G, Vorsters A, et al. Hepatitis B Vaccines. J Infect Dis. 2021;224(12 Suppl 2):S343-S51. doi: 10.1093/infdis/jiaa668

11. Ott JJ, Stevens GA, Groeger J, et al. Global epidemiology of hepatitis B virus infection: new estimates of age-specific HBsAg seroprevalence and endemicity. Vaccine. 2012;30(12):2212-9. doi: 10.1016/j.vaccine.2011.12.116

12. Andreasyan D, Bazarchyan A. "Health and Health Care" Yearbook. Yerevan: National Institute of Health Named after Academician S. Avdalbekyan; 2022. 299 p.

13. Demirchyan A, Mirzoyan L, Thompson ME. Synthesis of the existing data on Hepatitis B in Armenia. Yerevan: American University of Armenia, Center for Health Services Research; 2000. https://aua.am/chsr/PDF/2000/HepatitB_FINAL.pdf

14. Ghazinyan H, Mkhitaryan A, Melik-Andreasyan G, et al. Updating HBV status in Armenia. Optimal Management of Hepatitis B Virus Infection; 25–27 September Athens, Greece: Proceedings of EASL Special Conference; 2014. p. 159.

15. Melik-Andreasyan GG, Markosyan IE, Voskanyan LS, et al. Epidemiological Characteristics of Viral Hepatitis in Armenia (in Russian). В мире вирусных гепатитов. 2013;4:20-6.

16. Davlidova S, Haley-Johnson Z, Nyhan K, et al. Prevalence of HIV, HCV and HBV in Central Asia and the Caucasus: A systematic review. Int J Infect Dis. 2021;104:510-25. doi: 10.1016/j.ijid.2020.12.068

17. Roche Diagnostics. Elecsys® Anti-HBc II. Immunoassay for the qualitative determination of total antibodies against hepatitis B core antigen (HBcAg) [Available from: https://diagnostics.roche.com/global/en/products/params/elecsys-anti-hbc-ii.html].

For peer review only - http://bmjopen.bmj.com/site/about/guidelines.xhtml

18. Roche Diagnostics. Elecsys® HBsAg II. Immunoassay for the qualitative determination of hepatitis B surface antigen (HBsAg) [Available from: https://diagnostics.roche.com/global/en/products/params/elecsys-hbsag-ii.html].

19. Hosmer DW, Lemeshow S. Applied logistic regression. 2nd ed. New York: A Wiley-Interscience Publication; 2000: 92-116.

20. Khetsuriani N, Gamkrelidze A, Shadaker S, et al. Hepatitis B prevalence and the impact of vaccination in Georgia: results from a nationwide serosurvey International Liver Congress TM 2022, London22-26 June 2022 [Available from: https://www.postersessiononline.eu/173580348_eu/congresos/ILC2022/aula/-THU_283_ILC2022.pdf].

21. Polaris Observatory C. Global prevalence, treatment, and prevention of hepatitis B virus infection in 2016: a modelling study. Lancet Gastroenterol Hepatol. 2018;3(6):383-403. doi: 10.1016/S2468-1253(18)30056-6

22. Coalition for Global Hepatitis Elimination. Data Dashboard Armenia. [Available from: https://www.globalhep.org/country-progress/armenia] (accessed 27 July 2023).

23. Khetsuriani N, Tishkova F, Jabirov S, et al. Substantial decline in hepatitis B virus infections following vaccine introduction in Tajikistan. Vaccine. 2015;33(32):4019-24. doi: 10.1016/j.vaccine.2015.05.092

24. Robert Koch Institute. Report: Technical Assessment of Viral Hepatitis Response in Armenia. Berlin; 2023: 16-19.

25. Khan NU, Zalan A, Petruzziello A, et al. Determining the Actual Prevalence of Hepatitis B in Khyber Pakhtunkhwa-Pakistan: A Meta-Analysis. Open Virol J. 2018;12:33-41. doi: 10.2174/1874357901812010033

For peer review only

26. Wang H, Men P, Xiao Y, et al. Hepatitis B infection in the general population of China: a systematic review and meta-analysis. BMC Infect Dis. 2019;19(1):811. doi: 10.1186/s12879-019-4428-y

27. Brown R, Goulder P, Matthews PC. Sexual Dimorphism in Chronic Hepatitis B Virus (HBV) Infection: Evidence to Inform Elimination Efforts. Wellcome Open Res. 2022;7:32. doi: 10.12688/wellcomeopenres.17601.2

28. Ruggieri A, Gagliardi MC, Anticoli S. Sex-Dependent Outcome of Hepatitis B and C Viruses Infections: Synergy of Sex Hormones and Immune Responses? Front Immunol. 2018;9:2302. doi: 10.3389/fimmu.2018.02302

29. More countries reaching hepatitis B control targets brings the WHO European Region closer to eliminating viral hepatitis as a public health threat [press release]. https://www.who.int/europe/news/item/24-04-2023-more-countries-reaching-hepatitis-b-control-targets-brings-the-who-european-region-closer-to-eliminating-viral-hepatitis-as-a-public-health-threat2023 (accessed 25 April 2023).

30. Terrault NA, Wahed AS, Feld JJ, et al. Incidence and prediction of HBsAg seroclearance in a prospective multi-ethnic HBeAg-negative chronic hepatitis B cohort. Hepatology. 2022;75(3):709-23. doi: 10.1002/hep.32231

31. Galea S, Vlahov D. Social determinants and the health of drug users: socioeconomic status, homelessness, and incarceration. Public Health Rep. 2002;117 Suppl 1(Suppl 1):S135-45.

32. MacLachlan JH, Cowie BC. Hepatitis B virus epidemiology. Cold Spring Harb Perspect Med. 2015;5(5):a021410. doi: 10.1101/cshperspect.a021410

33. Corona R, Caprilli F, Giglio A, et al. Risk factors for hepatitis B virus infection among heterosexuals attending a sexually transmitted diseases clinic in Italy: role of genital ulcerative diseases. J Med Virol. 1996;48(3):262-6. doi: 10.1002/(SICI)1096-9071(199603)48:3<262::AID-JMV8>3.0.CO;2-6

For peer review only

34. McMaughan DJ, Oloruntoba O, Smith ML. Socioeconomic Status and Access to Healthcare: Interrelated Drivers for Healthy Aging. Front Public Health. 2020;8:231. doi: 10.3389/fpubh.2020.00231

35. Brodzinski A, Neumeyer-Gromen A, Dudareva S, et al. [Hepatitis B virus infection and vaccine-induced immunity: the role of sociodemographic determinants: Results of the study "German Health Interview and Examination Survey for Adults" (DEGS1, 2008-2011)]. Bundesgesundheitsblatt Gesundheitsforschung Gesundheitsschutz. 2022;65(2):159-69. doi: 10.1007/s00103-021-03473-z

36. Machmud PB, Glasauer S, Gottschick C, et al. Knowledge, Vaccination Status, and Reasons for Avoiding Vaccinations against Hepatitis B in Developing Countries: A Systematic Review. Vaccines (Basel). 2021;9(6). doi: 10.3390/vaccines9060625

37. Pereira LM, Martelli CM, Merchan-Hamann E, et al. Population-based multicentric survey of hepatitis B infection and risk factor differences among three regions in Brazil. Am J Trop Med Hyg. 2009;81(2):240-7.

38. European Association for the Study of the Liver. EASL 2017 Clinical Practice Guidelines on the management of hepatitis B virus infection. J Hepatol. 2017;67(2):370-98. doi: 10.1016/j.jhep.2017.03.021

39. Chen CJ, Yang HI, Su J, et al. Risk of hepatocellular carcinoma across a biological gradient of serum hepatitis B virus DNA level. JAMA. 2006;295(1):65-73. doi: 10.1001/jama.295.1.65

40. Liu CJ, Chen BF, Chen PJ, et al. Role of hepatitis B viral load and basal core promoter mutation in hepatocellular carcinoma in hepatitis B carriers. J Infect Dis. 2006;193(9):1258-65. doi: 10.1086/502978

41. Barcena Marugan R, Garcia Garzon S. DNA-guided hepatitis B treatment, viral load is essential, but not sufficient. World J Gastroenterol. 2009;15(4):423-30. doi: 10.3748/wjg.15.423

For peer review only - http://bmjopen.bmj.com/site/about/guidelines.xhtml

42. Mendy ME, Welzel T, Lesi OA, et al. Hepatitis B viral load and risk for liver cirrhosis and hepatocellular carcinoma in The Gambia, West Africa. J Viral Hepat. 2010;17(2):115-22. doi: 10.1111/j.1365-2893.2009.01168.x

43. Kim GA, Han S, Choi GH, et al. Moderate levels of serum hepatitis B virus DNA are associated with the highest risk of hepatocellular carcinoma in chronic hepatitis B patients. Aliment Pharmacol Ther. 2020;51(11):1169-79. doi: 10.1111/apt.15725

44. World Health Organization. Global Hepatitis Report. Geneva: Licence: CC BY-NC-SA 3.0 IGO.; 2017. https://apps.who.int/iris/bitstream/handle/10665/255016/9789241565455-eng.pdf?sequence=1&isAllowed=y

45. Polaris Observatory, C., Global prevalence, cascade of care, and prophylaxis coverage of hepatitis B in 2022: a modelling study. Lancet Gastroenterol Hepatol, 2023. Doi: 10.1016/S2468-1253(23)00197-8

Figure Legend:

Figure 1 Weighted prevalence estimates of HBV core antibody and HBV surface antigen by 10-year age groups among the study participants, Armenia, 2021.

For peer review only

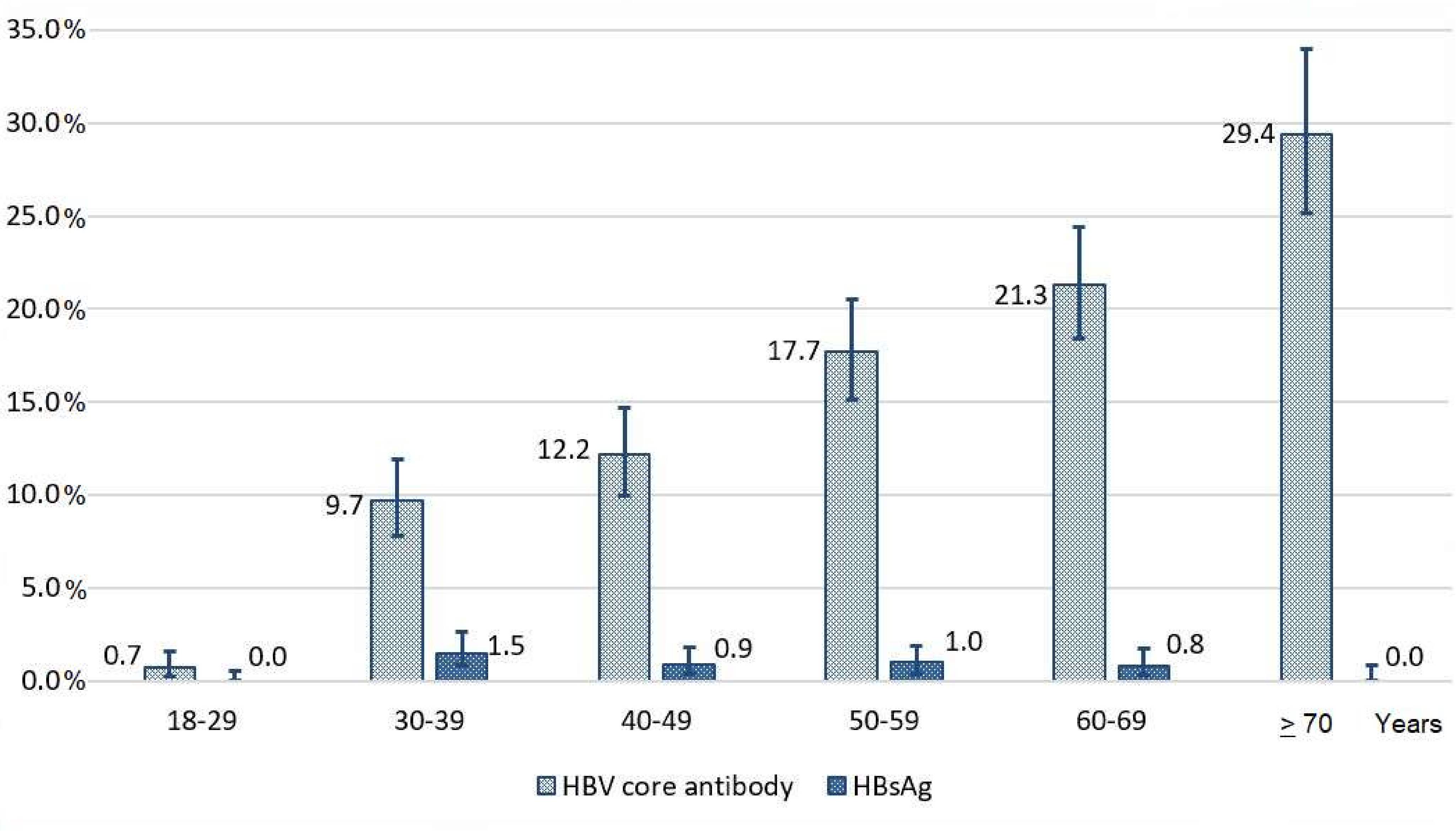
35.0%
30.0%
25.0%
20.0%
15.0%
10.0%
5.0%
0.0%
0.7
0.0
9.7
1.5
12.2
0.9
17.7
1.0
21.3
0.8
29.4
0.0
18-29
30-39
40-49
50-59
60-69
≥ 70
Years
HBV core antibody
HBsAg

Knowledge, Attitude, and Practices Questionnaire on Infectious Diseases, Chronic Hepatitis B and C Virus Infections and Associated Risk Factors

Interviewer

_ _ _ _ _ _ _

Participant ID

_ _/_ _/_ _ _/

Blood sampling for Hepatitis

Do not read out loud. Mark as it is mentioned in Excel file.

() Yes → *Show Section 17*

() No → *Skip Section 17*

Date

dd/mm/yy

______ / ______ / ____________

Start of the interview

___:___ (hour:min)

1. Socio-demographic characteristics

The following questions are about your demographic characteristics

1.1 Your nationality

( ) Armenian

( ) Russian

( ) Yazidi

( ) Other, please specify ________________________________

( ) [prefer not to answer]

1.2 Place of residence

( ) City

( ) Village

( ) [prefer not to answer]

For peer review only - http://bmjopen.bmj.com/site/about/guidelines.xhtml

1.3 Your gender

Do not read out loud

( ) Male

( ) Female

1.4 How old are you in the number of years completed? (How old were you on your last birthday)?

dd/mm/yy

_____ / _______ / ____

( ) [prefer not to answer]

1.5 Your highest level of education

Read out all of the options

( ) Incomplete secondary (less than 10 years)
( ) Secondary (10-12 years: high school)
( ) Vocational (12-13 years)
( ) University
( ) Post-graduate
( ) [prefer not to answer]

1.6 How many members are in your family, including yourself?

________________ members

( ) [prefer not to answer]

1.7 Are you currently employed?

Read out all of the options

() Yes, please specify what is your occupation?_________________

() No, I am not employed
() No, I am retired
() No, I am a student
() I work from home/ have my own business
() I am a seasonal worker in foreign countries
() I am a farmer
() Other (specify) ______________________

( ) [prefer not to answer]

For peer review only - http://bmjopen.bmj.com/site/about/guidelines.xhtml

For peer review only

1.8 On average, how much money does your family spend monthly? (Choose one option)

( ) Less than 100,000 AMD
( ) From 101,000 to 200,000 AMD
( ) From 201,000 to 300,000 AMD
( ) From 301,000 to 400,000 AMD
( ) Above 401,000 AMD
( ) Don't know/ Refusal

2. The following questions refer to health and infectious disease literacy.

2.1 How often do you need to have someone help you when you read instructions, pamphlets, or other written material from your doctor or pharmacy? (R*ead the response options, except [prefer not to answer] option)*

1. Never
2. Rarely
3. Sometimes
4. Often
5. Always
6. [prefer not to answer]

On a scale from 1 to 7, how easy or difficult would you say it is to…:

Note to interviewers: Repeat the full question as needed

2.2…find the information you need related to infectious diseases (for example, hepatitis, measles, influenza, COVID-19, HIV, etc.)?

Very difficult [1] [2] [3] [4] [5] [6] [7] Very easy

( ) [prefer not to answer]

2.3…understand information about what to do if you think you have an infectious disease (for example, measles, influenza, COVID-19, HIV, etc.)?

Very difficult [1] [2] [3] [4] [5] [6] [7] Very easy

( ) [prefer not to answer]

2.4…judge if the information about infectious diseases in the media is reliable?

Very difficult [1] [2] [3] [4] [5] [6] [7] Very easy

( ) [prefer not to answer]

For peer review only - http://bmjopen.bmj.com/site/about/guidelines.xhtml

2.5 …understand restrictions and recommendations of authorities regarding infectious diseases?

Very difficult [1] [2] [3] [4] [5] [6] [7] Very easy

( ) [prefer not to answer]

2.6 …follow the recommendations on how to protect yourself from infectious diseases?

Very difficult [1] [2] [3] [4] [5] [6] [7] Very easy

( ) [prefer not to answer]

2.7 …understand recommendations about when to stay at home from work/school, and when not to?

Very difficult [1] [2] [3] [4] [5] [6] [7] Very easy

( ) [prefer not to answer]

2.8 …follow recommendations about when to stay at home from work/school, and when not to?

Very difficult [1] [2] [3] [4] [5] [6] [7] Very easy

( ) [prefer not to answer]

2.9 …understand recommendations about when to engage in social activities, and when not to?

Very difficult [1] [2] [3] [4] [5] [6] [7] Very easy

( ) [prefer not to answer]

2.10…follow recommendations about when to engage in social activities, and when not to?

Very difficult [1] [2] [3] [4] [5] [6] [7] Very easy

( ) [prefer not to answer]

3. The following questions are about infectious disease-related knowledge and behaviors

3.1 What is the most effective measure for preventing measles? *(Do not read, check all that apply)*

1. Vaccination
2. Using medical masks
3. Avoiding contact with those who have measles
4. Other (specify)__________________________
5. Don’t know
6. [prefer not to answer]

For peer review only - http://bmjopen.bmj.com/site/about/guidelines.xhtml

3.2 Please indicate whether you agree or disagree with the following statement:

- Antibiotics are effective in treating influenza.
  1. Agree
  2. Disagree
  3. Don't know
  4. [prefer not to answer]

3.3 Please indicate whether you agree or disagree with the following statement:

- Influenza is the same as common cold.
  1. Agree
  2. Disagree
  3. Don't know
  4. [prefer not to answer]

3.4 Please indicate whether you agree or disagree with the following statement:

- Influenza is caused by a virus.
  1. Agree
  2. Disagree
  3. Don't know
  4. [prefer not to answer]

3.5 Do you usually cover your mouth when coughing or sneezing in public places?

1. Yes
2. No
3. [prefer not to answer]

3.6 Do you usually wash your hands before eating?

1. Yes
2. No
3. [prefer not to answer]

For peer review only

4. The following questions are about the symptoms you might have experienced in the past.

4.1 Have you had fever (≥ 37) within the **last six months**?

( ) Yes→ Date (mm/yy) and the highest temperature, (C°):

____

( ) No

( ) Do not remember

( ) [prefer not to answer]

For peer review only - http://bmjopen.bmj.com/site/about/guidelines.xhtml

4.2 Have you had any respiratory symptoms (sore throat, cough, runny nose, shortness of breath, etc.) in the last 3 months?

( ) If yes, specify the date of the last episode → mm/yy: ______________________________

( ) No → Move to section 12.4

( ) [prefer not to answer] → Move to section 12.4

4.3 Please indicate, if you have had any of the below-mentioned symptoms in the last 6 months?

Respiratory symptoms

	**Yes**	**No**	**[prefer not to answer]**
4.3.a. Sore throat	( )	( )	( )
4.3.b.Cough	( )	( )	( )
4.3.c. Runny nose	( )	( )	( )
4.3.d. Shortness of breath	( )	( )	( )

4.4. Other symptoms

	**Yes**	**No**	**[prefer not to answer]**
4.4.a. Muscle ache	( )	( )	( )
4.4.b. Joint ache	( )	( )	( )
4.4.c. Chills	( )	( )	( )
4.4.d. Rash	( )	( )	( )
4.4.e. Chest pain	( )	( )	( )
4.4.f Vomiting	( )	( )	( )
4.4.g Headache	( )	( )	( )
4.4.h Nausea	( )	( )	( )

For peer review only

For peer review only - http://bmjopen.bmj.com/site/about/guidelines.xhtml

	Yes	No	[prefer not to answer]
4.4.i Loss of taste	( )	( )	( )
4.4.g Loss of smell	( )	( )	( )
4.4.k. Loss of appetite	( )	( )	( )
4.4.l Abdominal pain	( )	( )	( )
4.4.m Diarrhea	( )	( )	( )
4.4.n Conjunctivitis	( )	( )	( )
4.4.o Nose bleed	( )	( )	( )
4.4.p Fatigue	( )	( )	( )
4.4.q Seizures	( )	( )	( )
4.4.r Altered consciousness	( )	( )	( )

4.5.a Other neurological signs

( ) Yes (specify): ____

( ) no

( ) [prefer not to answer]

4.5.b Other symptoms

( ) Yes (specify): ____

( ) No

( ) [prefer not to answer]

For peer review only

5. The following questions are about accompanying diseases and conditions.

Please indicate if you have had any of the below mentioned diseases/conditions.

5.1. Pregnancy

(Do not ask this question to males)

( ) Yes (specify the trimester: first, second, third, unknown):

____

( ) No

( ) [prefer not to answer]

*Read all of the options, marking **yes** or **no** in front of each. Do not read [prefer not to answer] option.*

	**Yes**	**No**	**Don't know/Refuse to answer**
5.2. Obesity	( )	( )	
5.3. Cancer	( )	( )	
5.4. Diabetes	( )	( )	
5.5. HIV/ other immune deficiency	( )	( )	
5.6. Heart disease	( )	( )	
5.7. Asthma (requiring medication)	( )	( )	
5.8. Chronic lung disease (non-asthma)	( )	( )	
5.9. Chronic liver disease	( )	( )	
5.10. Chronic hematological disorder	( )	( )	
5.11. Chronic kidney disease	( )	( )	
5.12. Chronic neurological impairment/disease	( )	( )	
5.13. Organ or bone marrow recipient	( )	( )	

For peer review only - http://bmjopen.bmj.com/site/about/guidelines.xhtml

5.14. Other accompanying disease(s)

( ) Yes (specify) ____

( ) No

( ) [prefer not to answer]

6. Have you smoked more than 100 cigarettes during your life?

( ) Yes

( ) No

( ) [prefer not to answer]

6.a How would you describe your current smoking status?

Read out all of the options

( ) I smoke everyday – go to question 16.b

( ) I smoke less than everyday

( ) I quit smoking less than a year ago

( ) I quit smoking more than a year ago

( ) [prefer not to answer]

6.b How many cigarettes do you smoke per day? ___________

7. The following questions are about factors that might be associated with Hepatitis C and B infection in the population

7.1 How often do you visit the dentist to have your teeth cleaned, tooth extracted, braces put on, or root canals?

( ) Twice per year

( ) Once a year

( ) Once every 2 years

( ) Once every 3-5 years

( ) It has been more than 5 years

( ) Never

( ) Don't know/remember/[prefer not to answer]

For peer review only

For peer review only - http://bmjopen.bmj.com/site/about/guidelines.xhtml

7.2. Have you received any blood transfusions in your lifetime?

( ) Yes, before 199X
( ) Yes, in or after 199X
( ) No
( ) [prefer not to answer]

7.3 Do you have any tattoos?

( ) Yes
( ) No
( ) [prefer not to answer]

7.4 Have you ever been told by a doctor or other health worker that you had hepatitis B or C? *(multiple answers possible)*

( ) Yes hepatitis B
( ) Yes hepatitis C
( ) Yes, but don't know the type
( ) Neither hepatitis B nor hepatitis C
( ) I am vaccinated against hepatitis B
( ) Don't know/remember/refuse to answer

7.5 Have you ever been in prison (translation: long-term detainment) or jail (translation: short-term detainment)?

( ) Yes
( ) No
( ) [prefer not to answer]

End of interview

___:___ (hour:min)

Notes

____

Thank you!

For peer review only - http://bmjopen.bmj.com/site/about/guidelines.xhtml

For peer review only

STROBE Statement—Checklist of items that should be included in reports of ***cross-sectional studies***

	Item No	Recommendation	Page No
**Title and abstract**	1	(*a*) Indicate the study's design with a commonly used term in the title or the abstract	1
		(*b*) Provide in the abstract an informative and balanced summary of what was done and what was found	1
**Introduction**			
Background/rationale	2	Explain the scientific background and rationale for the investigation being reported	3-4
Objectives	3	State specific objectives, including any prespecified hypotheses	5
**Methods**			
Study design	4	Present key elements of study design early in the paper	5-6
Setting	5	Describe the setting, locations, and relevant dates, including periods of recruitment, exposure, follow-up, and data collection	5-6
Participants	6	(*a*) Give the eligibility criteria, and the sources and methods of selection of participants	6
Variables	7	Clearly define all outcomes, exposures, predictors, potential confounders, and effect modifiers. Give diagnostic criteria, if applicable	7-8
Data sources/ measurement	8*	For each variable of interest, give sources of data and details of methods of assessment (measurement). Describe comparability of assessment methods if there is more than one group	*7-8*
Bias	9	Describe any efforts to address potential sources of bias	6-7
Study size	10	Explain how the study size was arrived at	6
Quantitative variables	11	Explain how quantitative variables were handled in the analyses. If applicable, describe which groupings were chosen and why	9
Statistical methods	12	(*a*) Describe all statistical methods, including those used to control for confounding	9-10
		(*b*) Describe any methods used to examine subgroups and interactions	NA
		(*c*) Explain how missing data were addressed	10
		(*d*) If applicable, describe analytical methods taking account of sampling strategy	9
		(*e*) Describe any sensitivity analyses	NA
**Results**			
Participants	13*	(a) Report numbers of individuals at each stage of study—eg numbers potentially eligible, examined for eligibility, confirmed eligible, included in the study, completing follow-up, and analysed	10
		(b) Give reasons for non-participation at each stage	NA
		(c) Consider use of a flow diagram	NA
Descriptive data	14*	(a) Give characteristics of study participants (eg demographic, clinical, social) and information on exposures and potential confounders	11-14
		(b) Indicate number of participants with missing data for each variable of interest	11-14
Outcome data	15*	Report numbers of outcome events or summary measures	10-11

For peer review only - http://bmjopen.bmj.com/site/about/guidelines.xhtml

Main results	16	(*a*) Give unadjusted estimates and, if applicable, confounder-adjusted estimates and their precision (eg, 95% confidence interval). Make clear which confounders were adjusted for and why they were included	10
		(*b*) Report category boundaries when continuous variables were categorized	8
		(*c*) If relevant, consider translating estimates of relative risk into absolute risk for a meaningful time period	NA
Other analyses	17	Report other analyses done—eg analyses of subgroups and interactions, and sensitivity analyses	NA
**Discussion**			
Key results	18	Summarise key results with reference to study objectives	17
Limitations	19	Discuss limitations of the study, taking into account sources of potential bias or imprecision. Discuss both direction and magnitude of any potential bias	20
Interpretation	20	Give a cautious overall interpretation of results considering objectives, limitations, multiplicity of analyses, results from similar studies, and other relevant evidence	20-21
Generalisability	21	Discuss the generalisability (external validity) of the study results	18
**Other information**			
Funding	22	Give the source of funding and the role of the funders for the present study and, if applicable, for the original study on which the present article is based	21

*Give information separately for exposed and unexposed groups.

Note: An Explanation and Elaboration article discusses each checklist item and gives methodological background and published examples of transparent reporting. The STROBE checklist is best used in conjunction with this article (freely available on the Web sites of PLoS Medicine at http://www.plosmedicine.org/, Annals of Internal Medicine at http://www.annals.org/, and Epidemiology at http://www.epidem.com/). Information on the STROBE Initiative is available at www.strobe-statement.org.

For peer review only
